# Protease-Activated Receptor-2 as a Proteolytic Rheostat in Colorectal and Pancreatic Cancer: From Mechanism to Biomarker-Guided Therapy

**DOI:** 10.3390/ijms27177526

**Published:** 2026-08-22

**Authors:** Hodasadat Tabatabaei Yeganeh, Malak Sellat, Zayd Anis, Reine Chiri, Rajashree Patnaik, Shloka Gambhir, Yajnavalka Banerjee

**Affiliations:** College of Medicine, Mohammed Bin Rashid University of Medicine and Health Sciences, Dubai Health, Dubai 505055, United Arab Emirates; 202500907@students.mbru.ac.ae (H.T.Y.); 202500977@students.mbru.ac.ae (M.S.); 202500875@students.mbru.ac.ae (Z.A.); 202501143@students.mbru.ac.ae (R.C.); shloka.gambhir@gmail.com (S.G.)

**Keywords:** PAR-2, *F2RL1*, protease-activated receptor, colorectal cancer, pancreatic ductal adenocarcinoma, tissue factor, FXa, ERK, NF-κB, β-catenin, tumour microenvironment, drug repurposing, statins, nutraceuticals, biomarkers

## Abstract

Protease-activated receptor-2 (PAR-2; encoded by *F2RL1*) is emerging as a context-dependent driver of gastrointestinal cancer. Activated by irreversible N-terminal cleavage, its signalling output is not fixed but is calibrated by the identity and source of the activating protease, the receptor cleavage state, cellular context and biased coupling to G-protein αq (Gαq), G-protein α12/13 (Gα12/13) and β-arrestin. PAR-2 is best understood not as a simple inflammatory receptor but as a proteolytic rheostat that converts diverse coagulation, inflammatory, microbial and stromal protease inputs into distinct oncogenic programmes. Colorectal cancer and pancreatic ductal adenocarcinoma provide complementary models: in colorectal cancer, PAR-2 links mucosal inflammation and coagulation to proliferation, metastatic competence and resistance to epidermal growth factor receptor (EGFR)-targeted therapy, whereas in pancreatic cancer, it is embedded in a tissue-factor-rich desmoplastic microenvironment that promotes invasion, immune exclusion and chemoresistance. Therapeutic strategies suggested by this framework include direct and biased PAR-2 modulators, upstream protease and factor Xa (FXa) inhibition, statin repurposing and activated-fragment biomarkers such as the PAR-2 activation neoepitope (PRO-PAR2). These strategies must be applied under biomarker guidance, since PAR-2 blockade may benefit inflammation-dominant tumours yet prove counterproductive where PAR-2 sustains antitumour immunity.

## 1. Introduction

Gastrointestinal malignancies remain among the most lethal human cancers, and colorectal cancer (CRC) and pancreatic ductal adenocarcinoma (PDAC) together account for a substantial proportion of cancer-related mortality worldwide. Despite advances in cytotoxic, targeted and immune-based therapy, durable responses remain the exception in advanced disease, in large part because both tumours evolve within biochemically hostile microenvironments in which proteolysis, inflammation and coagulation are continuously active. A unifying theme to emerge over the past two decades is that extracellular protease activity is not merely a bystander of tissue injury but an instructive signal that is transduced into intracellular programmes through a dedicated family of cell-surface sensors, the protease-activated receptors (PARs). Among these, protease-activated receptor-2 (PAR-2), encoded by the *F2RL1* gene, has emerged as the principal receptor linking the protease-rich ecology of the gastrointestinal tract to oncogenic, inflammatory, stromal and immune signalling. This review introduces the protease–PAR-2 axis as an integrative framework for understanding gastrointestinal carcinogenesis, sets out the molecular logic of PAR-2 activation, and then develops the central argument that PAR-2 behaves not as a binary inflammatory switch but as a context-dependent proteolytic rheostat whose output is dictated by the identity and source of the activating protease, the cellular and oncogenic context, and the temporal pattern of receptor engagement. The sections that follow are organised to build this framework systematically, from receptor architecture and biased agonism through disease-specific biology in CRC and PDAC to therapeutic targeting, biomarker strategy and precision-oncology stratification.

### 1.1. Gastrointestinal Cancers as Protease-Rich Diseases

Colorectal cancer (CRC) and pancreatic ductal adenocarcinoma (PDAC) are united by a deceptively simple biological feature: both evolve in tissue compartments that are unusually rich in proteolytic activity. CRC develops at the interface between an inflamed colonic mucosa, a complex microbial community whose members secrete diverse proteases, and a stromal niche populated by mast cells, neutrophils, fibroblasts and endothelial cells. PDAC is embedded in a fibroinflammatory desmoplastic stroma in which the constitutive protease biology of the pancreas is amplified by tumour-cell tissue factor (TF) expression, coagulation activation, neutrophil serine protease release, mast-cell tryptase secretion and the proteolytic remodelling activities of cancer-associated fibroblasts (CAFs) [1,2,3,4,5,6,7]. Within this proteolytic milieu, the four mammalian protease-activated receptors (PARs) function as the principal sensors that convert extracellular protease activity into intracellular signalling, with PAR-2 (encoded by *F2RL1*) emerging as the dominant receptor in inflamed and protease-rich epithelia [7,8,9,10,11].

Two recent observations sharpen the rationale for a focused mechanistic synthesis. First, a 2025 Cell Host and Microbe study identified *Bacteroides fragilis* serine protease 1 (Bfp1) as a microbiome-derived activator of host PAR-2 that disrupts the intestinal barrier, sensitises nociceptors, and triggers colonic inflammation [12], providing evidence that microbial proteolysis can directly engage the host PAR-2 axis under conditions relevant to inflammation-driven CRC. Second, multi-omics analyses of ulcerative colitis cohorts have linked *Bacteroides vulgatus* protease activity to disease severity [6], and a 2025 cryo-EM structural study of PAR-2 in complex with miniGs/q and miniG13 has provided an atomic-level account of tethered-ligand engagement and biased agonism [13], together establishing PAR-2 as both a tractable structural target and a node positioned at the host–microbe interface most relevant to colorectal carcinogenesis.

### 1.2. PAR-2 as an Inflammatory Receptor and as a Cancer Signalling Hub

**Figure 1:**
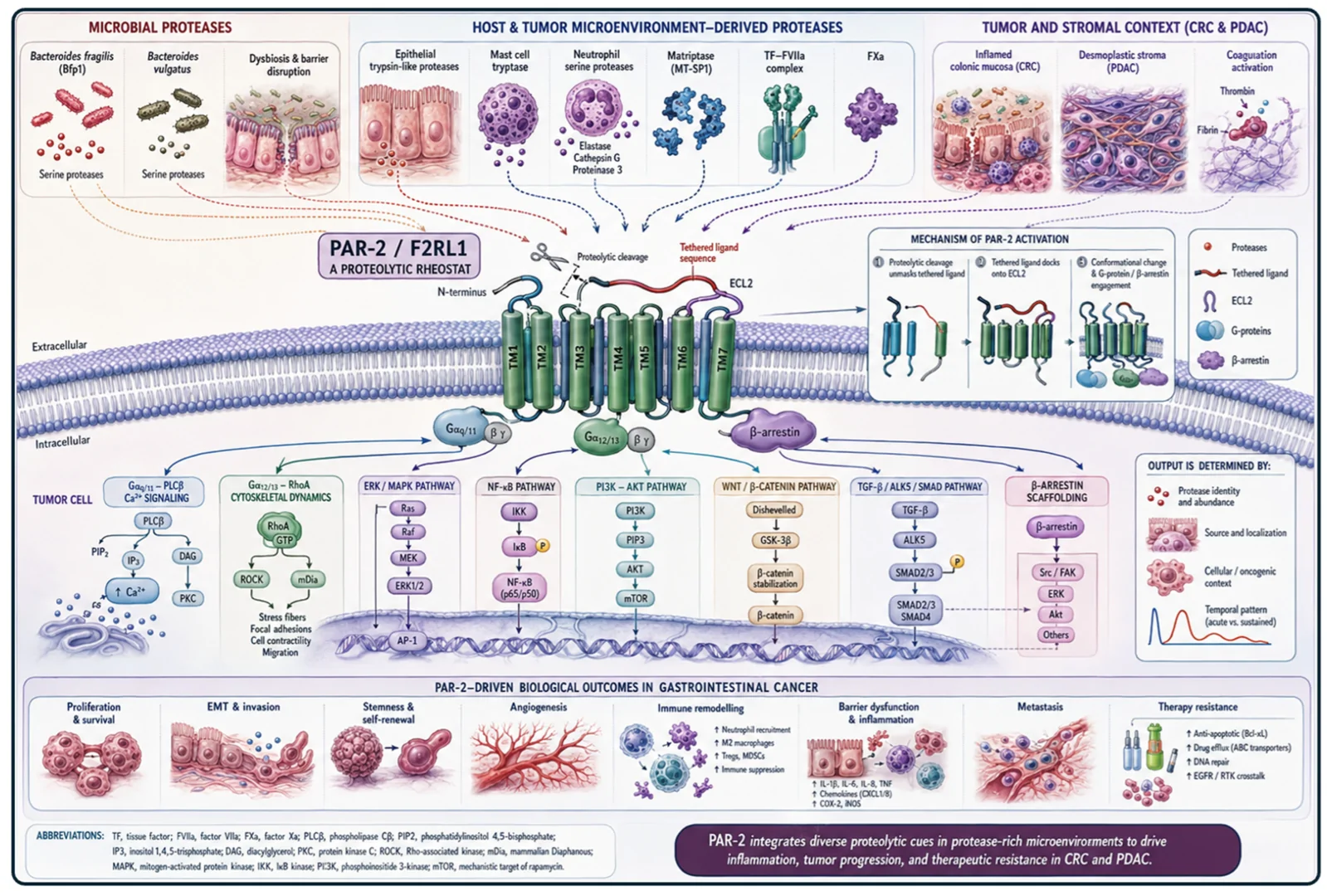
PAR-2/*F2RL1* as a proteolytic signalling rheostat in gastrointestinal cancer. The figure summarises the concept that protease-activated receptor-2 (PAR-2/*F2RL1*) integrates diverse proteolytic cues from microbial, epithelial, inflammatory, stromal and coagulation-derived sources into context-dependent oncogenic signalling programmes in colorectal cancer (CRC) and pancreatic ductal adenocarcinoma (PDAC). PAR-2 is depicted as a seven-transmembrane G-protein-coupled receptor embedded in the tumour-cell plasma membrane. Proteolytic cleavage of the extracellular N-terminus exposes the tethered ligand sequence, which engages extracellular loop 2 (ECL2) and the transmembrane bundle, triggering receptor conformational change, G-protein coupling and β-arrestin recruitment. The upper panels show major classes of PAR-2-regulating proteases in the gastrointestinal tumour microenvironment. Microbial proteases derived from dysbiotic organisms, including *Bacteroides fragilis* and *Bacteroides vulgatus*, are shown alongside host and tumour microenvironment-derived proteases, including epithelial trypsin-like proteases, mast-cell tryptase, neutrophil elastase, cathepsin G, proteinase 3, matriptase, the tissue factor–FVIIa complex and factor Xa (FXa). These protease inputs arise in distinct biological contexts: the inflamed colonic mucosa of CRC, the desmoplastic stroma of PDAC, and the coagulation-activated tumour microenvironment. The central panel illustrates PAR-2 activation by proteolytic cleavage and tethered-ligand engagement. Following cleavage of the N-terminal domain, the exposed tethered ligand binds intramolecularly to ECL2 and the transmembrane core, promoting coupling to Gαq/11, Gα12/13, Gβγ subunits and β-arrestin. The inset illustrates the stepwise mechanism of PAR-2 activation: N-terminal cleavage, tethered-ligand docking, and conformational rearrangement enabling G-protein or β-arrestin engagement. The figure emphasises that PAR-2 output is determined not only by receptor abundance but also by protease identity and abundance, protease source and localisation, cellular and oncogenic context, and the temporal pattern of receptor activation. The lower signalling panels show the principal downstream pathways activated by PAR-2 in gastrointestinal cancer. These include Gαq/11–PLCβ–IP3/DAG–Ca^2+^ signalling, Gα12/13–RhoA–ROCK cytoskeletal remodelling, ERK/MAPK, NF-κB, PI3K–AKT, Wnt/β-catenin, TGF-β/ALK5/SMAD and β-arrestin-dependent scaffolding. Together, these pathways regulate calcium mobilisation, focal adhesion dynamics, inflammatory transcription, survival signalling, β-catenin stabilisation, epithelial plasticity, and compartmentalised endosomal signalling. The bottom panel integrates these pathway outputs into biological consequences relevant to CRC and PDAC, including proliferation and survival, EMT and invasion, cancer stem-cell self-renewal, angiogenesis, immune remodelling, barrier dysfunction and inflammation, metastasis and therapy resistance. Overall, the figure frames PAR-2 as a proteolytic rheostat rather than a simple inflammatory ON/OFF receptor: distinct protease landscapes in CRC and PDAC generate biased PAR-2 signalling outputs that shape tumour progression, stromal remodelling, immune contexture and therapeutic response. Arrows indicate the direction of protease input, receptor activation, downstream signalling and biological effects; colours distinguish protease classes, pathway modules and outcome categories. Abbreviations: *F2RL1*, *F2R* like trypsin receptor 1; FVIIa, activated factor VII; Gα, G-protein alpha subunit; Gβγ, G-protein beta–gamma subunits; PLCβ, phospholipase C beta; IP3, inositol 1,4,5-trisphosphate; DAG, diacylglycerol; ROCK, Rho-associated protein kinase; ERK, extracellular signal-regulated kinase; MAPK, mitogen-activated protein kinase; NF-κB, nuclear factor kappa B; PI3K, phosphoinositide 3-kinase; AKT, protein kinase B; TGF-β, transforming growth factor beta; ALK5, activin receptor-like kinase 5; SMAD, small mothers against decapentaplegic; EMT, epithelial–mesenchymal transition. [Note: Created in BioRender. Banerjee, Y. (2026) https://BioRender.com/owamu02, accessed on 30 July 2026].

PAR-2 belongs to the seven-transmembrane G-protein-coupled receptor (GPCR) superfamily but, unlike most GPCRs, is irreversibly activated by N-terminal proteolytic cleavage that exposes a tethered peptide ligand which docks onto extracellular loop 2 (ECL2) and rearranges the transmembrane core to engage Gαq, Gα12/13 and β-arrestins (Figure 1) [9,14,15,16]. Trypsin, mast-cell tryptase, kallikreins, matriptase, the tissue factor (TF)–activated factor VII (FVIIa) complex, and factor Xa (FXa) are the principal physiological activators (Figure 1) [1,16,17,18,19], and the receptor signals through phospholipase C (PLC)–inositol 1,4,5-trisphosphate (IP3)-driven Ca^2+^ mobilisation, the rapidly accelerated fibrosarcoma (RAF)–mitogen-activated protein kinase kinase (MEK)–extracellular signal-regulated kinase (ERK) mitogen-activated protein kinase (MAPK) cascade, phosphoinositide 3-kinase (PI3K)–protein kinase B (AKT), nuclear factor κB (NF-κB), and the Rho guanosine triphosphatase (GTPase) axis [14,20,21]. The conceptual leap of the present review is to argue that, in CRC and PDAC, PAR-2 should not be treated as an inflammatory ‘ON/OFF’ switch but as a proteolytic rheostat whose output is determined by the identity, abundance, localisation and source of the activating protease; by the cellular and oncogenic context in which the receptor is engaged; and by the temporal pattern (acute vs. sustained) of activation.

### 1.3. Why Focus on Colorectal and Pancreatic Cancer?

**Figure 2:**
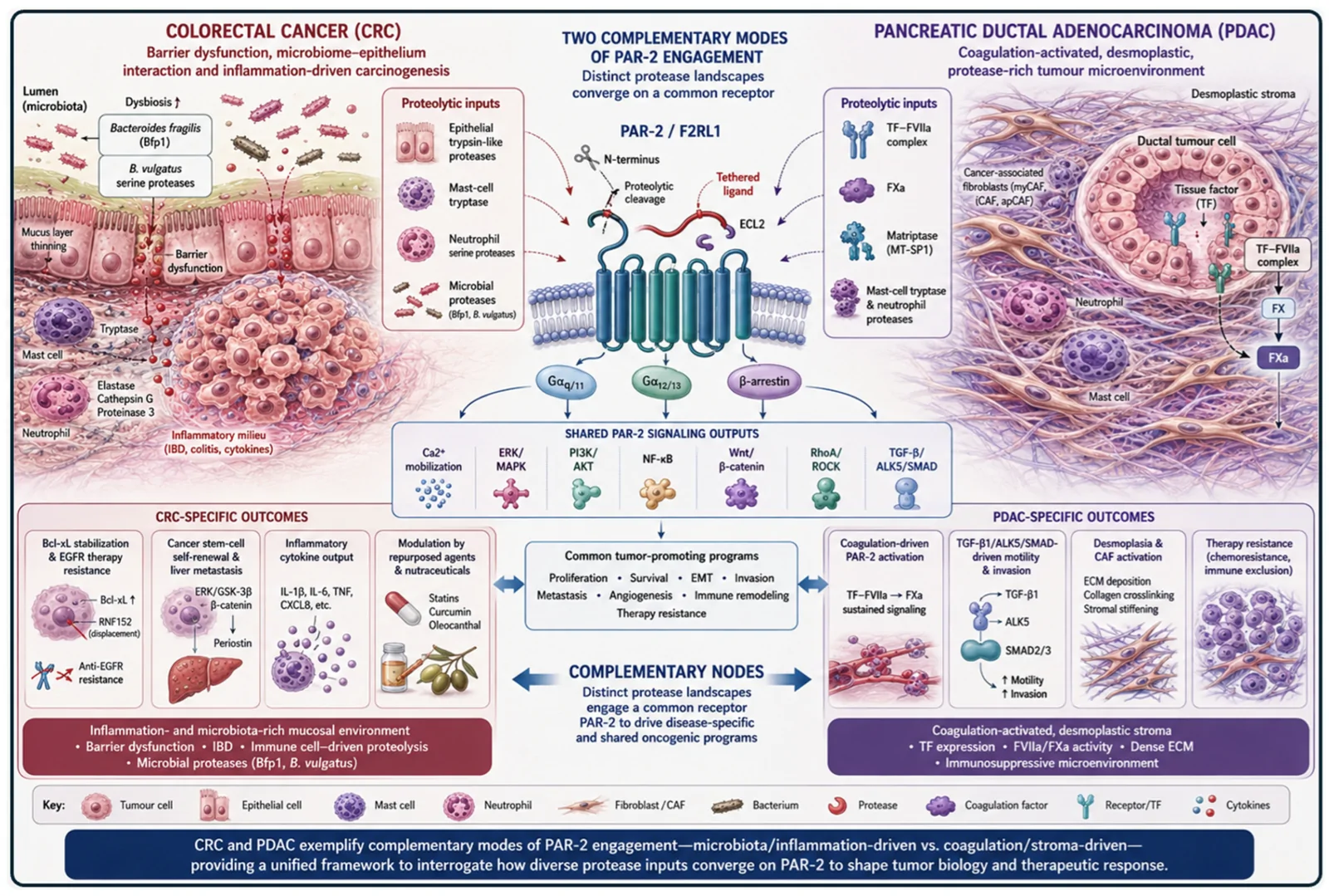
Colorectal cancer and pancreatic ductal adenocarcinoma as complementary modes of PAR-2 engagement in gastrointestinal cancer. The figure illustrates how colorectal cancer (CRC) and pancreatic ductal adenocarcinoma (PDAC) represent biologically complementary settings in which distinct protease landscapes converge on a common receptor, protease-activated receptor-2 (PAR-2/*F2RL1*), to generate shared and disease-specific oncogenic outputs. CRC is depicted as an inflammation- and microbiota-rich mucosal disease, whereas PDAC is shown as a coagulation-activated, desmoplastic and stroma-rich malignancy. In CRC, PAR-2 is positioned at the interface between the intestinal lumen, dysbiotic microbiota, barrier dysfunction and the inflamed tumour microenvironment. Microbial proteases derived from organisms such as *Bacteroides fragilis* and *Bacteroides vulgatus*, epithelial trypsin-like proteases, mast-cell tryptase and neutrophil serine proteases are shown as dominant inputs that cleave or modulate PAR-2. These proteolytic signals promote mucosal inflammation, calcium signalling, NF-κB activation, cytokine output, Wnt/β-catenin amplification, cancer stem-cell self-renewal, metastatic competence and resistance to EGFR-targeted therapy, including mechanisms involving Bcl-xL stabilisation and disruption of RNF152-mediated ubiquitination. In PDAC, PAR-2 is embedded within a dense desmoplastic tumour microenvironment enriched in cancer-associated fibroblasts, immune cells, tissue factor expression and coagulation proteases. Tumour-cell tissue factor promotes formation of the TF–FVIIa complex and local generation of FXa, creating a sustained coagulation-linked PAR-2 activation loop. Additional inputs include matriptase, mast-cell tryptase, neutrophil proteases and stromal proteases. These signals converge on PAR-2 to support TGF-β1/ALK5/SMAD-driven motility and invasion, CAF activation, extracellular-matrix deposition, angiogenesis, immune exclusion and therapy resistance. The central schematic depicts PAR-2 as a seven-transmembrane GPCR activated by proteolytic cleavage of its extracellular N-terminus, exposing a tethered ligand that engages the receptor and promotes coupling to Gαq/11, Gα12/13 and β-arrestin. Despite their different proteolytic ecologies, CRC and PDAC share downstream PAR-2 signalling outputs, including calcium mobilisation, ERK/MAPK, PI3K–AKT, NF-κB, Wnt/β-catenin, RhoA/ROCK and TGF-β/ALK5/SMAD pathways. These shared pathways converge on common tumour-promoting programmes such as proliferation, survival, epithelial–mesenchymal transition, invasion, metastasis, angiogenesis, immune remodelling and therapeutic resistance. (Note: Overall, the figure frames CRC and PDAC as complementary nodes within the protease–PAR-2 framework: CRC exemplifies microbiota- and inflammation-driven PAR-2 activation at a disrupted mucosal barrier, whereas PDAC exemplifies coagulation-, tissue factor- and stroma-driven PAR-2 activation within a desmoplastic tumour niche. Comparing these two diseases provides a unified mechanistic framework for understanding how distinct protease inputs engage the same receptor to produce both shared and disease-specific cancer phenotypes). Arrows show the direction from protease inputs through PAR-2 signalling to shared and disease-specific outcomes; colours distinguish CRC, PDAC and shared pathway modules. [Note: Created in BioRender. Banerjee, Y. (2026) https://BioRender.com/sgbusfw, accessed on 30 July 2026].

CRC and PDAC capture two complementary modes of PAR-2 engagement that together test the breadth of the protease-PAR-2 framework (Figure 2). CRC offers a setting in which barrier dysfunction, microbiome–epithelium interactions, inflammatory bowel disease (IBD)-associated carcinogenesis, mast-cell tryptase activity and PAR-2-driven inflammatory signalling can be dissected with accessible cellular models; recent CRC studies have linked PAR-2 to Bcl-xL stabilisation and EGFR-targeted therapy resistance [22], to ERK/GSK-3β/β-catenin-driven cancer stem-cell self-renewal and liver metastasis through periostin [23], and to inflammatory cytokine output that can be modulated by repurposed statins [24], curcumin [25], and the olive-derived phenolic oleocanthal [26]. PDAC, by contrast, exemplifies a coagulation-activated, desmoplastic, protease-rich microenvironment in which TF expression by ductal tumour cells [1,2,3] sustains TF–FVIIa–FXa-driven PAR-2 signalling and in which PAR-2 is required for TGF-β1–ALK5–SMAD-driven motility and invasion [27,28,29,30]. Comparing these two diseases under one mechanistic framework, therefore, allows interrogation of how distinct protease landscapes engage a common receptor.

## 2. Molecular Architecture and Activation Biology of PAR-2

### 2.1. The F2RL1 Gene, Receptor Topology and Tethered-Ligand Activation

**Figure 3:**
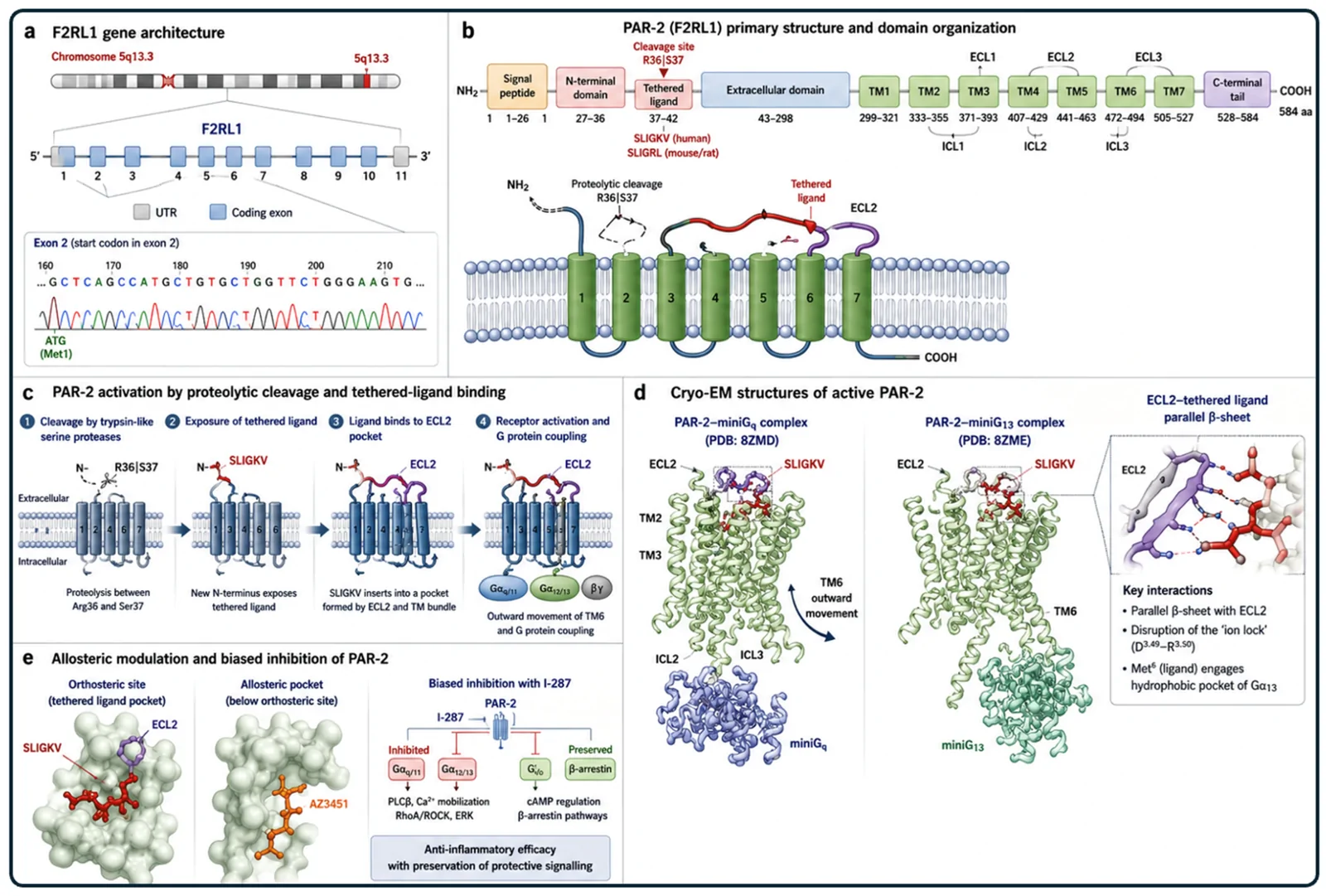
Molecular architecture, tethered-ligand activation and structural pharmacology of PAR-2/*F2RL1*. (**a**) Schematic representation of the human *F2RL1* locus on chromosome 5q13.3, showing exon organisation and the coding region that gives rise to protease-activated receptor-2. (**b**) Linear and membrane-topology representations of PAR-2, a class-A seven-transmembrane GPCR. The extracellular N-terminus contains the canonical trypsin-like protease cleavage site between Arg36 and Ser37, which exposes the human tethered ligand SLIGKV. The receptor contains seven transmembrane helices, three extracellular loops, three intracellular loops and a cytoplasmic C-terminal tail. The tethered ligand engages a binding pocket formed by ECL2 and the transmembrane bundle. (**c**) Stepwise model of PAR-2 activation. Trypsin-like serine proteases cleave the N-terminus at Arg36–Ser37, generating a new N-terminus that functions as an intramolecular tethered ligand. Ligand insertion into the ECL2/transmembrane pocket promotes rearrangement of the receptor core, including outward displacement of TM6, enabling coupling to Gαq/11, Gα12/13, Gβγ and β-arrestin-dependent signalling modules. (**d**) Structural basis of active PAR-2 signalling derived from recent cryo-EM structures of PAR-2 in complex with engineered G-protein mimetics. The PAR-2–miniG13 complex is represented by PDB: 8ZME, and the companion PAR-2–miniGs/q structure is reported as PDB: 8ZMD. These structures show that the tethered ligand forms a parallel β-sheet interaction with ECL2, promotes disruption of an inhibitory ion-lock interaction, and enables G-protein engagement at the intracellular receptor interface. (**e**) Pharmacological modulation of PAR-2. Orthosteric ligand engagement occurs at the tethered-ligand pocket, whereas small-molecule negative allosteric modulators such as AZ3451 exploit a pocket below the orthosteric site. Biased inhibitors such as I-287 illustrate the feasibility of selectively suppressing PAR-2-dependent Gαq/11 and Gα12/13 outputs while sparing Gi/o- and β-arrestin-associated signalling. Together, these structural and pharmacological insights support the concept of PAR-2 as a ligand-, protease-, and context-dependent signalling rheostat rather than a simple inflammatory ON/OFF receptor. Arrows indicate the direction of receptor activation and structural transitions; colours distinguish gene/receptor domains, interacting components and pharmacological sites. [Note: Created in BioRender. Banerjee, Y. (2026) https://BioRender.com/lq0font, accessed on 30 July 2026].

PAR-2 is encoded by *F2RL1* on chromosome 5q13 and is widely expressed across epithelial, endothelial, smooth-muscle, and immune compartments [13,29,31,32]. Activation occurs through cleavage of the extracellular N-terminus by trypsin-like serine proteases between Arg-36 and Ser-37, exposing the tethered ligand SLIGKV (human) or SLIGRL (rodent), which inserts into a pocket flanked by ECL2 and the transmembrane bundle (Figure 3) [31]. Cryo-EM structures of active PAR-2 in complex with miniGs/q and miniG13 have shown that the tethered ligand forms a parallel β-sheet with ECL2 that triggers an outward shift of TM6 and the disruption of an inhibitory ‘ion lock’, with hydrophobic engagement of the C-terminal methionine of Gα13 dictating coupling selectivity [31] (Figure 3). An earlier crystallographic study had defined an allosteric pocket below the orthosteric site that can be exploited by the negative allosteric modulator AZ3451 and related ligands [33], and the small-molecule I-287 has demonstrated that selective inhibition of PAR-2 Gαq and Gα12/13 signalling, while leaving Gi/o and β-arrestin pathways intact, is sufficient to blunt PAR-2-driven inflammatory responses in vivo [14].

### 2.2. Biased Agonism and Protease-Specific Signalling Outputs

A central feature of PAR-2 biology is that not all activation events are equivalent. Different proteases cleave PAR-2 at canonical or non-canonical sites and produce distinct ‘biased’ signalling outputs (Table 1). Trypsin and mast-cell tryptase generate the canonical SLIGKV/SLIGRL tethered ligand and engage the full Gαq/Gα12/13/β-arrestin repertoire [5,9], whereas neutrophil elastase and cathepsin G can cleave PAR-2 at sites that disable canonical signalling but preserve biased β-arrestin-dependent MAPK output [34]. Synthetic peptide agonists (SLIGKV-NH2, 2-furoyl-LIGRLO) recapitulate canonical signalling but should not be assumed to fully model the biology of physiological proteases, an important methodological caveat in interpreting cellular studies [21,35]. Coagulation system activation through TF–FVIIa, with subsequent local generation of FXa, can engage PAR-2 either directly or through the ternary TF–FVIIa–FXa complex [16], and the oncogenic outputs of TF–FVIIa-driven PAR-2 signalling have been documented in breast and pancreatic cancer, where they include AKT-driven GSK-3β inactivation, β-catenin stabilisation and IL-8/VEGF-driven angiogenesis [36,37].

### 2.3. PAR-2 as a Membrane-Localised Proteolytic Rheostat

Synthesising these observations, we propose that PAR-2 is best conceptualised as a membrane-localised proteolytic rheostat that does not merely report the presence or absence of protease activity but encodes the identity, source, intensity, and duration of proteolysis. Different cellular sources, including tumour epithelial cells, coagulation, mast cells, neutrophils, microbiome, and CAFs (cancer-associated fibroblasts), generate qualitatively distinct ‘protease codes’ that engage PAR-2 with characteristic downstream consequences (Table 1; Figure 3). Recognising this distinction is critical because it implies that quantification of *F2RL1* mRNA, or even total PAR-2 protein, captures only one dimension of the receptor’s biology; activation status, ligand source, and downstream pathway engagement must also be resolved.

## 3. PAR-2 Signalling Networks Relevant to Cancer Biology

### 3.1. Gq–Phospholipase C–Calcium Signalling

**Figure 4:**
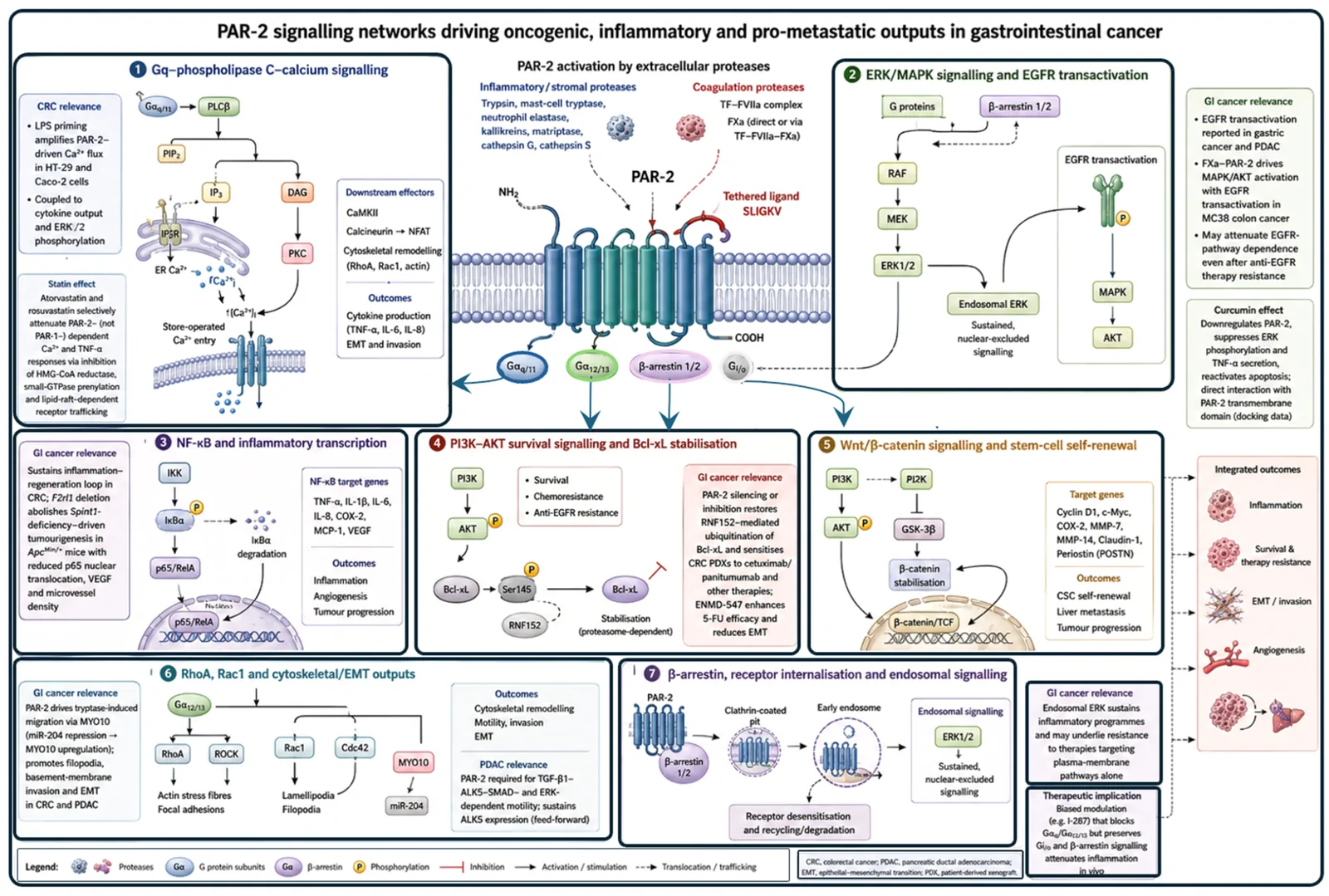
PAR-2 signalling networks driving oncogenic, inflammatory and pro-metastatic outputs in gastrointestinal cancer. The figure summarises the major intracellular signalling programmes activated downstream of protease-activated receptor-2 (PAR-2/*F2RL1*) in colorectal cancer and pancreatic ductal adenocarcinoma. Extracellular proteases derived from inflammatory, stromal, epithelial, microbial and coagulation-associated sources cleave the extracellular N-terminus of PAR-2, exposing the tethered ligand SLIGKV and enabling receptor coupling to Gαq/11, Gα12/13, Gi/o and β-arrestin-dependent signalling modules. The central schematic depicts PAR-2 as a seven-transmembrane GPCR embedded in the tumour-cell plasma membrane, with pathway-specific branches radiating from the receptor to illustrate how proteolytic activation is converted into calcium, MAPK, inflammatory, survival, Wnt, cytoskeletal and endosomal signalling outputs. (1) Gαq–phospholipase C–calcium signalling. PAR-2 coupling to Gαq/11 activates PLCβ, leading to hydrolysis of PIP2 into IP3 and diacylglycerol (DAG). IP3 promotes calcium release from the endoplasmic reticulum through IP3 receptors, whereas store depletion triggers store-operated calcium entry. This calcium signal supports CaMKII activation, calcineurin–NFAT engagement, cytoskeletal remodelling and inflammatory cytokine production. In CRC models, LPS priming amplifies PAR-2-dependent calcium fluxes in HT-29 and Caco-2 cells, linking protease sensing to ERK phosphorylation and cytokine output. Statins are shown as selective attenuators of PAR-2-, but not PAR-1-, dependent calcium and TNF-α responses, consistent with altered small-GTPase prenylation and membrane-receptor trafficking. (2) ERK/MAPK signalling and EGFR transactivation. PAR-2 activates the RAF–MEK–ERK1/2 cascade through both G-protein-dependent and β-arrestin-dependent mechanisms. β-arrestin scaffolding supports a sustained, endosomal pool of ERK activity that is spatially distinct from acute plasma-membrane MAPK signalling. PAR-2 also promotes EGFR transactivation, thereby linking extracellular protease activity to receptor tyrosine kinase signalling, MAPK amplification and AKT activation. This axis is relevant to gastric cancer, PDAC and CRC models, including FXa–PAR-2-mediated MAPK/AKT activation with EGFR transactivation. Curcumin is indicated as a PAR-2-modulating compound that suppresses ERK phosphorylation, TNF-α secretion and inflammatory survival signalling. (3) NF-κB and inflammatory transcription. PAR-2 activation engages the IKK–IκBα–p65/RelA pathway, leading to IκBα phosphorylation and degradation, nuclear translocation of p65/RelA, and induction of inflammatory and angiogenic genes, including TNF-α, IL-1β, IL-6, IL-8, COX-2, MCP-1 and VEGF. This module is particularly important in CRC, where chronic PAR-2-dependent inflammatory transcription may sustain an inflammation–regeneration loop that promotes adenoma-to-carcinoma progression. The figure also highlights genetic evidence from intestinal tumorigenesis models in which *F2rl1* deletion attenuates p65 nuclear translocation, VEGF expression and microvessel density. (4) PI3K–AKT survival signalling and Bcl-xL stabilisation. PAR-2-driven PI3K–AKT signalling promotes survival, chemoresistance and resistance to targeted therapy. In CRC, PAR-2 activation stabilises the anti-apoptotic protein Bcl-xL through phosphorylation at Ser145, which prevents RNF152-mediated ubiquitination and proteasomal degradation. This mechanism links PAR-2 signalling directly to apoptotic resistance and provides a rationale for combining PAR-2 inhibition with EGFR-targeted therapies or cytotoxic regimens. (5) Wnt/β-catenin signalling and stem-cell self-renewal. PAR-2 amplifies β-catenin-dependent transcription through PI3K–AKT-mediated inhibition of GSK-3β and through ERK/GSK-3β-dependent pathways. Stabilised β-catenin translocates to the nucleus and cooperates with TCF/LEF to induce target genes involved in proliferation, invasion and stemness, including cyclin D1, c-Myc, COX-2, MMP-7, MMP-14, claudin-1 and periostin. This pathway supports CRC cancer stem-cell self-renewal, liver metastasis and tumour progression. (6) Gα12/13–RhoA/Rac1 signalling regulates cytoskeletal remodelling and motility. (7) β-arrestin-associated receptor internalisation supports compartmentalised endosomal ERK signalling. Arrows indicate activation or signal flow, blunt-ended lines indicate inhibition, dashed arrows indicate translocation or trafficking, and colours distinguish pathway modules and cellular compartments. [Note: Created in BioRender. Banerjee, Y. (2026) https://BioRender.com/6djymfp, accessed on 30 July 2026].

PAR-2 activation engages Gαq, leading to phospholipase C-β-mediated cleavage of phosphatidylinositol-4,5-bisphosphate, generation of IP3 and diacylglycerol, IP3-receptor-mediated calcium release from the endoplasmic reticulum and store-operated calcium entry (Figure 4) [38,39]. In CRC cell lines, lipopolysaccharide (LPS) priming amplifies trypsin- and tryptase-driven PAR-2-dependent calcium fluxes, and we and others have shown that this calcium signal is closely coupled to inflammatory cytokine output and ERK1/2 phosphorylation in HT-29 and Caco-2 cells [24,25,26]. Calcium-dependent transcriptional and migratory programmes, including CaMKII activation, calcineurin/NFAT engagement and cytoskeletal remodelling, provide a mechanistic basis for the link between PAR-2-driven calcium flux and EMT/invasion [40,41,42]. Crucially, the statin pharmacology described in our recent work demonstrated that atorvastatin and rosuvastatin selectively attenuated PAR-2-, but not PAR-1-, dependent calcium and TNF-α responses in inflamed CRC cell lines [24,43], consistent with HMG-CoA reductase-driven control of small-GTPase prenylation and lipid-raft-dependent receptor trafficking [44].

### 3.2. ERK/MAPK Signalling and EGFR Transactivation

PAR-2 activation drives RAF–MEK–ERK1/2 phosphorylation through both G-protein-dependent and β-arrestin-dependent mechanisms; the latter sustains a pool of activated ERK that is restricted to endosomes and decoupled from nuclear translocation (Figure 4) [20,45]. In *KRAS*- or *BRAF*-mutant CRC, PAR-2 may not initiate but rather amplify, sustain or spatially redirect MAPK signalling, integrating extracellular protease activity into the dominant oncogenic output [46,47]. EGFR transactivation has been documented in gastric cancer and PDAC models in which PAR-2 promotes proliferation and migration through transactivation of EGFR [19,48], and recent work on MC38 colon cancer showed that FXa engagement of PAR-2 drives MAPK and AKT activation accompanied by EGFR transactivation [49]. This mechanism suggests that PAR-2 inhibition might attenuate EGFR-pathway dependence even in tumours that have escaped first-line anti-EGFR therapy through downstream rewiring. We have shown that curcumin selectively downregulates PAR-2, suppresses ERK phosphorylation and TNF-α secretion, and reactivates apoptotic signalling in inflammation-driven CRC cells, with computational docking suggesting direct interaction of curcumin with the transmembrane domain of PAR-2 [25].

### 3.3. NF-κB and Inflammatory Transcription

PAR-2 activation engages the IKK–IκBα–p65/RelA axis, with consequent nuclear translocation of p65 and induction of TNF-α, IL-1β, IL-6, IL-8, COX-2, MCP-1 and VEGF (Figure 4) [9,50]. This NF-κB-centred output is particularly relevant in CRC, where it sustains an inflammation–regeneration loop that lowers the threshold for adenoma-to-carcinoma progression. In *Apc*^Min/+^ mice, *F2rl1* deletion abolished the accelerated tumour formation caused by intestinal *Spint1* (HAI-1) deficiency and was associated with reduced p65 nuclear translocation, lower VEGF expression and reduced microvessel density [50,51], providing genetic evidence that PAR-2-dependent NF-κB signalling is causally linked to intestinal carcinogenesis in vivo. In our experimental work, oleocanthal selectively reduced PAR-2-driven inflammatory output in CRC cell lines through suppression of NF-κB- and Wnt/β-catenin-coupled programmes [26,52].

### 3.4. PI3K–AKT Survival Signalling and Bcl-xL Stabilisation

PAR-2-driven AKT activation contributes to anti-apoptotic and pro-survival programmes that become especially relevant in the context of cytotoxic and targeted therapies (Figure 4). The most translationally compelling evidence in CRC was provided by Li and colleagues, who showed that PAR-2 activation phosphorylates Bcl-xL at Ser-145, displacing the E3 ubiquitin ligase RNF152 from Bcl-xL and stabilising the anti-apoptotic protein in a proteasome-dependent manner; PAR-2 silencing or chemical inhibition restored RNF152-mediated Bcl-xL ubiquitination and sensitised CRC patient-derived xenografts to multiple EGFR-targeted therapies [22]. This finding establishes a direct mechanistic link between protease–PAR-2 signalling and the apoptotic competence of CRC cells, and frames PAR-2 antagonism as a rational sensitiser to cetuximab/panitumumab. Independent work has shown that PAR-2 inhibition with ENMD-547 enhanced 5-FU efficacy and reduced EMT signalling in CRC models [53], further supporting a survival-pathway role for the receptor.

### 3.5. Wnt/β-Catenin Signalling and Stem-Cell Self-Renewal

Although *APC* loss is the canonical initiator of Wnt activation in CRC, PAR-2 functions as a non-canonical amplifier of β-catenin signalling (Figure 4). Roy et al. demonstrated in MDA-MB-231 breast cancer cells that FVIIa-driven PAR-2 activation engages PI3K–AKT-driven GSK-3β inactivation and β-catenin stabilisation with downstream upregulation of cyclin D1, c-Myc, COX-2, MMP-7, MMP-14 and claudin-1 [36]. In CRC, Ma et al. showed that PAR-2 controls cancer stem-cell symmetric/asymmetric division through ERK–GSK-3β–β-catenin signalling, with periostin emerging as a direct β-catenin target gene that mediates self-renewal; PAR-2 knockdown attenuated liver metastasis in xenograft models, and PAR-2 expression correlated with β-catenin and periostin in metastatic CRC primary sites [23]. Bar-Shavit et al. have further shown that PAR-2 acts cooperatively with PAR-4 in colon cancer to drive β-catenin stabilisation, with the β-catenin–p53 axis serving as the integrative oncogenic node [54,55], and that the cell-surface E3 ubiquitin ligase RNF43 normally constrains PAR-2 turnover but is suppressed in colon adenocarcinomas, releasing PAR-2-driven Wnt signalling [56].

### 3.6. RhoA, Rac1 and Cytoskeletal/EMT Outputs

Gα12/13 coupling drives PAR-2-dependent RhoA–ROCK and Rac1–Cdc42 activation, which shape filopodia, lamellipodia, and basement-membrane invasion (Figure 4) [33,57]. In CRC, PAR-2 has been shown to drive tryptase-induced cell migration through MYO10, an actin-based motor protein, with the regulatory loop involving repression of miR-204 and de-repression of MYO10 expression [58]. MYO10 itself promotes filopodia formation and contributes to the invasive phenotype, supporting a model in which PAR-2 controls a cytoskeletal programme that translates extracellular protease activity into tumour-cell motility. In PDAC, PAR-2 is required for TGF-β1-induced cell motility through ALK5-dependent SMAD signalling and through ERK reactivation; PAR-2 sustains the expression of the TGF-β type I receptor ALK5 itself, providing a feed-forward mechanism that amplifies TGF-β-driven invasion [27,29,30].

### 3.7. β-Arrestin Recruitment, Receptor Internalisation and Endosomal Signalling

β-arrestins recruited to activated PAR-2 not only mediate receptor desensitisation and internalisation but also scaffold ERK/MAPK signalling at endosomes (Figure 4) [20]. This compartmentalised signalling produces a sustained ERK pool that is functionally distinct from acute plasma-membrane MAPK output and is implicated in PAR-2-driven inflammatory programmes [14,20]. Therapeutically, this has direct consequences: orthosteric antagonists, negative allosteric modulators and biased ligands will modulate different subsets of PAR-2 outputs, and the I-287 modulator which blocks Gαq/Gα12/13 without affecting Gi/o or β-arrestin output is sufficient to attenuate inflammation in vivo [14], suggesting that complete pathway blockade may not be required or even desirable for clinical activity.

## 4. PAR-2 in Colorectal Cancer

### 4.1. Expression Patterns in Normal Mucosa, Adenoma and Carcinoma

**Figure 5:**
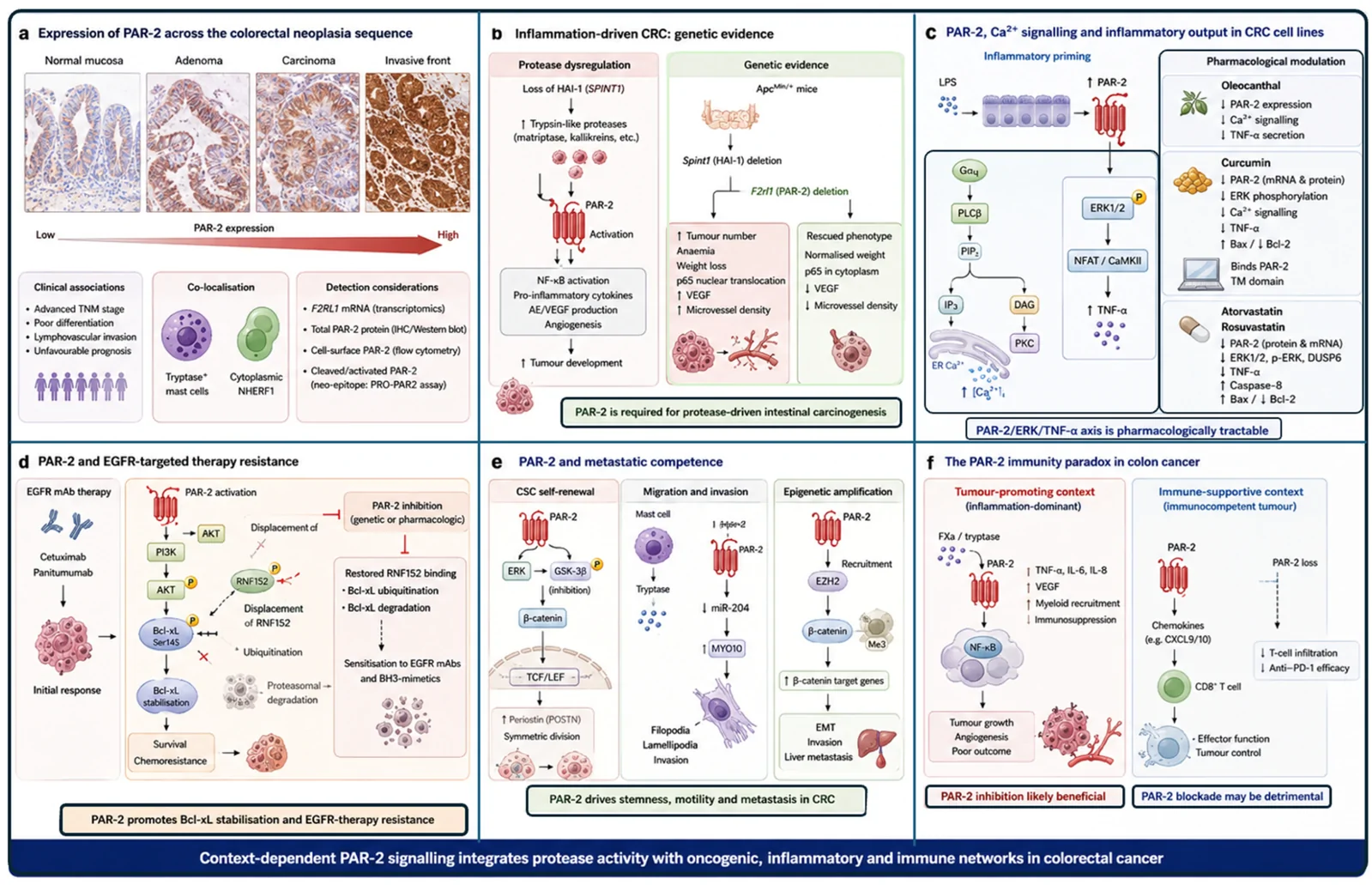
PAR-2 in colorectal cancer: expression, mechanisms and therapeutic opportunities. The figure summarises the context-dependent roles of protease-activated receptor-2 (PAR-2/*F2RL1*) in colorectal cancer (CRC), integrating expression patterns across the colorectal neoplasia sequence, inflammation-driven tumour initiation, calcium-dependent inflammatory signalling, treatment resistance, metastatic competence and immune-context-dependent effects. Together, these mechanisms frame PAR-2 as a protease-sensitive signalling node that connects mucosal inflammation, tumour-cell survival, epithelial plasticity, stromal remodelling and therapeutic response. (**a**) Expression of PAR-2 across the colorectal neoplasia sequence. PAR-2 is detectable in normal colonic epithelium and becomes progressively enriched in adenomas, carcinomas and invasive tumour fronts. Increased PAR-2 immunoreactivity is associated with adverse clinicopathological features, including advanced TNM stage, poor differentiation, lymphovascular invasion and unfavourable prognosis [5]. The panel also highlights co-localisation with tryptase-positive mast cells and cytoplasmic NHERF1, suggesting that PAR-2 expression should be interpreted within a broader protease-rich and scaffold-dependent tumour microenvironment. Because *F2RL1* mRNA abundance does not necessarily reflect activated cell-surface PAR-2, the figure emphasises the need to integrate transcriptomics, total PAR-2 immunohistochemistry, cell-surface receptor detection and neo-epitope-based assays of cleaved PAR-2. (**b**) Inflammation-driven CRC and genetic evidence for PAR-2-dependent intestinal tumorigenesis. Loss of the endogenous serine-protease inhibitor HAI-1/*SPINT1* deregulates trypsin-like protease activity, including matriptase- and kallikrein-associated proteolysis, leading to PAR-2 activation, NF-κB signalling, pro-inflammatory cytokine production, VEGF induction, angiogenesis and accelerated intestinal tumour formation. In *Apc*^Min/+^ models, co-deletion of *F2rl1*/PAR-2 rescues the tumour-promoting phenotype associated with *SPINT1* deficiency, reducing tumour burden, p65 nuclear translocation, VEGF expression and microvessel density. This provides direct genetic evidence that PAR-2 is required for protease-driven intestinal carcinogenesis [50,51]. (**c**) PAR-2, calcium signalling and inflammatory output in CRC cell lines. In inflammatory CRC models, LPS priming enhances PAR-2 expression and sensitises HT-29 and Caco-2 cells to PAR-2-dependent calcium flux, ERK1/2 phosphorylation and TNF-α secretion. PAR-2 engagement activates the Gαq–PLCβ–PIP2–IP3/DAG axis, promoting endoplasmic-reticulum calcium release, protein kinase C activation and calcium-dependent inflammatory signalling through NFAT/CaMKII-linked programmes. Pharmacological modulation by oleocanthal, curcumin, atorvastatin and rosuvastatin attenuates PAR-2 expression and/or downstream ERK, calcium and TNF-α outputs, supporting the therapeutic tractability of the PAR-2/ERK/TNF-α axis in inflammation-driven CRC [24,25,26]. (**d**) PAR-2 and EGFR-targeted therapy resistance through the Bcl-xL/RNF152 axis. PAR-2 activation promotes survival signalling through PI3K–AKT-dependent stabilisation of the anti-apoptotic protein Bcl-xL. Phosphorylation of Bcl-xL at Ser145 disrupts its interaction with the E3 ubiquitin ligase RNF152, thereby preventing ubiquitination and proteasomal degradation. This mechanism contributes to resistance to EGFR-targeted monoclonal antibodies such as cetuximab and panitumumab. Genetic or pharmacological PAR-2 inhibition restores RNF152-mediated Bcl-xL turnover and sensitises CRC models to EGFR-targeted therapy, with additional evidence supporting sensitisation to 5-fluorouracil through reduction of EMT-associated signalling [22,53]. (**e**) PAR-2 and metastatic competence in CRC. PAR-2 promotes metastatic behaviour through three interconnected mechanisms: enhancement of cancer stem-cell self-renewal, cytoskeletal remodelling and epigenetic reinforcement of β-catenin signalling. PAR-2 activates the ERK/GSK-3β/β-catenin axis, increasing β-catenin/TCF-dependent transcription and induction of pro-metastatic targets such as periostin. In parallel, mast-cell tryptase activates PAR-2 to drive migration through repression of miR-204 and de-repression of MYO10, supporting filopodia, lamellipodia and invasive motility. PAR-2 also facilitates β-catenin transcriptional output through recruitment of epigenetic regulators such as EZH2, linking receptor activation to EMT, invasion and liver metastatic competence [23,54,55,57,58]. (**f**) The PAR-2 immunity paradox in colon cancer. PAR-2 signalling has context-dependent effects on tumour immunity. In inflammation-dominant tumours, FXa-, tryptase- or other protease-driven PAR-2 activation may enhance NF-κB-dependent cytokine production, angiogenesis, myeloid recruitment, immunosuppression and tumour growth, making PAR-2 inhibition potentially beneficial. However, in selected immunocompetent CRC settings, tumour-intrinsic PAR-2 may sustain chemokine production, CD8^+^ T-cell infiltration and antitumour effector function; in such contexts, PAR-2 loss may reduce immune visibility and impair anti-PD-1 efficacy. This duality indicates that PAR-2-directed therapy should not be developed as universal receptor blockade, but as biomarker-guided modulation based on protease context, immune phenotype and tumour inflammatory state [49,54]. Arrows indicate activation or progression, blunt-ended lines indicate inhibition, and colours distinguish the six mechanistic panels and their pathway components. [Note: Created in BioRender. Banerjee, Y. (2026) https://BioRender.com/2tioxb6, accessed on 30 July 2026].

PAR-2 is detectable in normal colonic epithelium and is upregulated in adenomas and carcinomas, with the highest expression typically observed at the invasive front (Figure 5). In a 115-patient CRC cohort, Malfettone et al. showed that PAR-2 immunoreactivity correlated with advanced TNM stage, poor differentiation and lymphovascular invasion, and that it co-localised with high tryptase-positive mast-cell density and with cytoplasmic NHERF1, a combined immunophenotype associated with unfavourable prognosis [5]. An important caveat is that *F2RL1* mRNA does not equate to activated PAR-2 protein at the cell surface; receptor turnover, internalisation, and the proportion of total PAR-2 that has been cleaved by physiological proteases all influence functional output. We therefore recommend that future expression studies combine *F2RL1* transcriptomics, total PAR-2 immunohistochemistry, cell-surface flow cytometry and, where feasible, neo-epitope-specific detection of cleaved PAR-2 fragments such as the PRO-PAR2 assay developed by Kalogera et al. [59].

### 4.2. PAR-2 and Inflammation-Driven Colorectal Cancer

The mechanistic linkage between PAR-2 and inflammation-driven CRC is anchored by genetic evidence from the matriptase/HAI-1 axis (Figure 5). *Spint1* (HAI-1) deletion in the *Apc*^Min/+^ intestine accelerated tumour formation through deregulated trypsin-like protease activity, NF-κB activation and pro-inflammatory cytokine production [51], and Kawaguchi et al. subsequently showed that this acceleration was abolished by *F2rl1* (PAR-2) co-deletion, with rescued tumour numbers, normalised anaemia and weight, and reduced p65 nuclear translocation, VEGF expression and microvessel density [50]. This represents some of the most direct in vivo genetic evidence that PAR-2 is required for protease-driven intestinal carcinogenesis, and that matriptase, kallikreins and other PAR-2 activating proteases are the relevant upstream drivers when their endogenous inhibitors fail (Table 1).

### 4.3. PAR-2, Calcium Signalling and Inflammatory Cytokine Output in CRC Cell Lines

Our recent work has dissected this biology in HT-29 and Caco-2 cells primed by LPS to mimic an inflammatory microenvironment. We have shown that LPS exposure upregulates PAR-2 expression and sensitises cells to PAR-2-dependent calcium and TNF-α responses (Figure 5); that selective phenolic agents (oleocanthal) reduce PAR-2 expression with concomitant attenuation of calcium signalling and TNF-α secretion [26]; that curcumin downregulates PAR-2 at transcript and protein levels, suppresses ERK phosphorylation and calcium signalling, inhibits TNF-α secretion and reverses the anti-apoptotic Bcl-2/Bax balance, with computational docking identifying a high-affinity binding interaction between curcumin and the PAR-2 transmembrane domain [25]; and that atorvastatin and rosuvastatin selectively attenuate PAR-2 (but not PAR-1) expression at protein and transcript levels with parallel suppression of total ERK1/2, p-ERK, DUSP6 and TNF-α and engagement of dual extrinsic (caspase-8) and intrinsic apoptotic programmes [24]. Together, these studies establish HT-29/Caco-2 as informative cellular platforms for dissecting the PAR-2/ERK/TNF-α axis in inflammation-driven CRC and provide proof of concept that the axis is pharmacologically tractable through repurposed and nutraceutical agents.

### 4.4. PAR-2 and EGFR-Targeted Therapy Resistance: The Bcl-xL/RNF152 Axis

EGFR-targeted antibody therapy (cetuximab, panitumumab) is approved for *KRAS*/RAS wild-type metastatic CRC but is limited by acquired resistance. Li et al. showed that PAR-2 expression positively correlated with Bcl-xL in CRC, that PAR-2 activation phosphorylated Bcl-xL at Ser-145 and stabilised it through disruption of its interaction with RNF152, and, crucially, that PAR-2 silencing or chemical inhibition sensitised CRC patient-derived xenografts to multiple EGFR-targeted therapies in vivo (Figure 5) [22]. The implication is that the protease–PAR-2 axis is a context-dependent driver of EGFR-therapy resistance acting downstream of receptor occupancy, and that PAR-2 antagonism could be combined with anti-EGFR therapy or with BH3-mimetics targeting Bcl-xL to deepen response. Independent evidence that PAR-2 inhibition with ENMD-547 sensitises CRC cells to 5-FU and reduces EMT signalling [53] extends this principle to standard cytotoxic chemotherapy.

### 4.5. PAR-2 and Metastatic Competence in CRC

Beyond proliferation and survival, PAR-2 contributes to metastatic competence through stem-cell self-renewal and cytoskeletal remodelling. Ma et al. established that PAR-2 expression was higher in cancer stem-cell (CSC) populations than in non-CSCs, that PAR-2 inhibition switched CSC division mode from symmetric to asymmetric through the ERK/GSK-3β/β-catenin axis, and that periostin acted as a direct β-catenin target driving CSC self-renewal; PAR-2 knockdown reduced liver metastasis in xenografts, and PAR-2 expression correlated with β-catenin and periostin in metastatic CRC [23]. Mast-cell tryptase activation of PAR-2 in turn drives MYO10-dependent migration through repression of miR-204 in CRC cell lines [58], and PAR-2 is required for the recruitment of EZH2 to β-catenin and the methylation-driven stabilisation of β-catenin transcriptional output [54,55]. Mechanistic targeting of PAR-2 may therefore disrupt multiple metastasis-supporting programmes simultaneously.

### 4.6. The PAR-2 Immunity Paradox in Colon Cancer

A balanced reading of the literature requires explicit acknowledgement that PAR-2’s role in tumour immunity is not uniformly tumour-promoting. The Meyer et al. (2025) study of FXa–PAR-2 in MC38 colon cancer demonstrated that genetic PAR-2 deficiency reduced tumour growth and improved health status in immunocompetent mice [49], consistent with a broadly tumour-promoting role. However, in some immunocompetent CRC models, tumour-intrinsic PAR-2 may sustain CD8^+^ T-cell infiltration and effector function through inflammatory chemokine output, with PAR-2 deficiency paradoxically attenuating anti-PD-1 efficacy. The implication is that universal PAR-2 blockade may not be uniformly beneficial across CRC immune phenotypes; biomarker-guided modulation that distinguishes inflammation-dominant tumours (where PAR-2 inhibition is likely beneficial) from tumours in which PAR-2 helps maintain immune visibility (where blockade may be counterproductive) is likely to be required. This is the most important conceptual caveat in the field and should be foregrounded in clinical-translational planning.

## 5. PAR-2 in Pancreatic Ductal Adenocarcinoma

### 5.1. Why PDAC Is a Prototypic PAR-2 Disease

**Figure 6:**
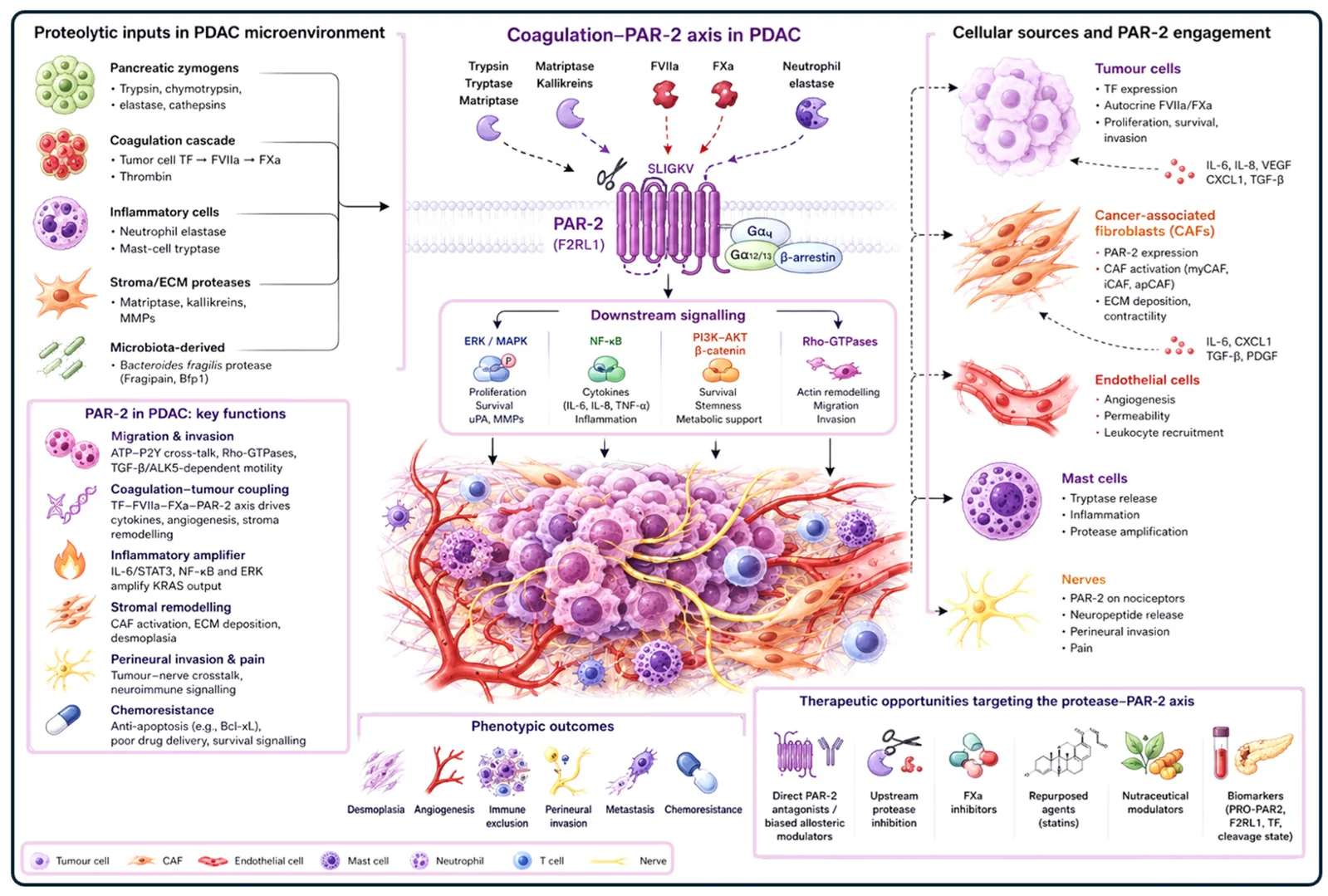
Coagulation–PAR-2 signalling in pancreatic ductal adenocarcinoma. The figure summarises the major proteolytic inputs, cellular sources, downstream signalling pathways and pathological consequences of protease-activated receptor-2 (PAR-2/*F2RL1*) activation in pancreatic ductal adenocarcinoma (PDAC). PDAC is depicted as a protease-rich, desmoplastic and coagulation-activated tumour microenvironment in which pancreatic zymogens, inflammatory-cell proteases, stromal matrix-remodelling enzymes, microbial proteases and the tissue factor–FVIIa–FXa axis converge on PAR-2 expressed by tumour cells, cancer-associated fibroblasts, endothelial cells, mast cells, infiltrating neutrophils and nerves. On the left, the principal proteolytic inputs in PDAC are shown. These include pancreatic zymogens and trypsin-like proteases, the coagulation cascade initiated by tumour-cell tissue factor (TF) with generation of FVIIa and FXa, mast-cell tryptase, neutrophil elastase, stromal/ECM proteases, including matriptase, kallikreins and matrix metalloproteinases, and microbiota-derived proteases such as *Bacteroides fragilis* protease. These inputs create a protease-saturated tumour niche in which PAR-2 can function as a high-gain signalling integrator. The central panel illustrates PAR-2 activation at the plasma membrane. Proteolytic cleavage of the extracellular N-terminus exposes the tethered ligand SLIGKV, enabling receptor coupling to Gαq, Gα12/13 and β-arrestin signalling modules. Downstream, PAR-2 activates ERK/MAPK, NF-κB, PI3K–AKT/β-catenin and Rho-GTPase pathways. These signalling branches support proliferation, survival, inflammatory cytokine production, uPA and matrix metalloproteinase expression, actin remodelling, migration, invasion, stemness-associated programmes and metabolic adaptation. The right panel highlights the major cellular compartments through which PAR-2 signalling may shape PDAC biology. In tumour cells, PAR-2 integrates TF–FVIIa/FXa and pancreatic protease signals to promote proliferation, invasion and survival. In cancer-associated fibroblasts (CAFs), PAR-2 may contribute to myCAF, iCAF and apCAF-associated phenotypes, extracellular-matrix deposition, contractility and secretion of cytokines such as IL-6, CXCL1, TGF-β and PDGF. In endothelial cells, PAR-2-linked signalling favours angiogenesis, permeability and leukocyte recruitment. Mast cells and neutrophils provide additional proteolytic amplification through tryptase, elastase and related serine proteases. In nerves and nociceptors, PAR-2 is positioned to contribute to neuropeptide release, perineural invasion and cancer-associated pain. The lower part of the figure integrates these signalling events into PDAC-relevant phenotypic outputs, including desmoplasia, angiogenesis, immune exclusion, perineural invasion, metastatic dissemination and chemoresistance. The therapeutic panel illustrates potential intervention points in the protease–PAR-2 axis, including direct PAR-2 antagonists or biased allosteric modulators, upstream protease inhibition, FXa inhibitors, repurposed agents such as statins, nutraceutical modulators and biomarker-guided strategies based on activated PAR-2 fragments, *F2RL1* expression, tissue factor status and receptor cleavage state. Overall, the figure positions PAR-2 as a central proteolytic signalling hub linking coagulation activation, stromal remodelling, neuroimmune cross-talk, invasion and treatment resistance in PDAC. Solid arrows indicate signalling or activation, dashed links indicate proposed intercellular crosstalk, and colours distinguish cellular compartments, protease sources and pathway modules. [Note: Created in BioRender. Banerjee, Y. (2026) https://BioRender.com/trscw25, accessed on 30 July 2026].

Pancreatic ductal adenocarcinoma is arguably the human tumour in which PAR-2 biology is most strongly anticipated on first principles (Figure 6). The pancreatic acinar compartment is the largest reservoir of trypsin-like serine proteases in the body, and ductal transformation by mutant *KRAS* occurs against a background of recurrent low-grade pancreatitis, persistent zymogen leakage, and tissue factor-driven thromboinflammation [60,61,62,63,64,65,66]. The desmoplastic stroma that is the histological hallmark of PDAC concentrates neutrophil elastase, mast-cell tryptase and CAF-derived matrix proteases at the tumour–stroma interface, while perineural invasion delivers tumour cells directly into a milieu rich in neuronal proteases and neuropeptide-driven proteolysis [67,68,69,70,71,72,73]. Tumour cells of PDAC characteristically express tissue factor, generating an autocrine and paracrine FVIIa/FXa signal that converges on PAR-2 in tumour cells, fibroblasts and endothelial cells alike [3,74,75,76,77,78]. Within this protease-saturated niche, PAR-2 is well placed to act as a high-gain integrator that translates pancreatic and coagulation proteolysis into the inflammatory, motile, fibrogenic and immune-exclusionary programmes that distinguish PDAC from most other epithelial cancers.

### 5.2. PAR-2-Driven Migration and ATP-Dependent Purinergic Cross-Talk

In a series of mechanistic experiments using PANC-1 and BxPC-3 pancreatic cancer cells, PAR-2 activation by trypsin or by the activating peptide SLIGRL-NH2 was shown to drive cell migration through a pathway that depended on extracellular ATP release and autocrine/paracrine engagement of P2Y purinergic receptors [48]. This observation places PAR-2 within a wider purinergic signalling architecture in which proteolytic and nucleotide cues cooperate to mobilise the actin cytoskeleton, raising the possibility that combined PAR-2 antagonism and purinergic blockade could synergistically restrain PDAC dissemination. The same study highlighted Gαq–PLC–IP3-mediated calcium signalling and Rho-GTPase remodelling as critical proximal events, both of which we and others have implicated in inflammation-driven PAR-2 biology in CRC [13,43], reinforcing the case for shared signalling machinery across gastrointestinal tumour types.

### 5.3. PAR-2 as a Permissive Co-Receptor for TGF-β-Driven Motility

Perhaps the most distinctive feature of PAR-2 biology in PDAC is its non-redundant role in TGF-β1-induced motility (Figure 6). Zeeh et al. demonstrated, using both PANC-1 and Colo357 cells, that PAR-2 silencing dramatically attenuated TGF-β1-induced migration and invasion through loss of expression of the type-I TGF-β receptor ALK5 [27]. Because ALK5 is the receptor that translates TGF-β1 binding into SMAD2/3 phosphorylation and downstream EMT transcription, PAR-2 functions in this setting as an upstream proteolytic gatekeeper of canonical TGF-β responsiveness, with implications for late-stage invasion and metastasis. Companion work has shown that the contribution of PAR-2 to TGF-β1/ALK5-driven migration is largely independent of Gαq–calcium output and is instead routed through Rho/Rac small GTPases and non-SMAD pathways [30,57], consistent with biased coupling of PAR-2 to G12/13 in motility-relevant cellular contexts. Witte and colleagues have argued that PAR-2 should therefore be considered a novel regulator of TGF-β signalling in pancreatic cancer rather than a parallel inflammatory input [29], an interpretation that materially expands the conceptual remit of PAR-2 in this disease.

### 5.4. The Tissue Factor–FVIIa–FXa–PAR-2 Axis in PDAC

Pancreatic adenocarcinoma is among the most thrombogenic of human malignancies, and tumour-cell tissue factor expression correlates with histological grade, invasion and prothrombotic complications (Figure 6) [3,4,79]. The TF–FVIIa binary complex generates an active proteolytic interface that, together with downstream FXa, cleaves and activates PAR-2 with high efficiency [16,76,80]. Coagulation–PAR-2 signalling drives expression of IL-8, VEGF and CXCL1 and contributes to angiogenesis, leukocyte recruitment and stromal remodelling in the tumour microenvironment [74,81,82]. A particularly informative line of evidence comes from breast cancer models showing that FVIIa/PAR-2 engagement triggers AKT activation and GSK-3β inhibition, with consequent β-catenin accumulation and tumour-promoting transcription [36]; analogous wiring is plausible in PDAC, where TF expression is more uniform than in most other epithelial tumours and FXa activity is essentially constitutive. Genetic and pharmacological dissection has shown that the cytoplasmic tail of tissue factor cooperates with PAR-2 to support tumour growth in vivo, with PAR-2-deficient hosts displaying delayed mammary carcinogenesis [18,83], a paradigm that frames the TF cytoplasmic domain–PAR-2 module as a druggable signalling unit. The translational relevance of this axis is evident in two recent observations: low-molecular-weight FXa inhibitors suppress PAR-2-dependent inflammatory cytokine release and tumour cell migration in colorectal models in vitro and reduce tumour growth in vivo [49,84], and PAR-2 silencing in pancreatic xenografts (Panc1) substantially reduces tumour growth [85], indicating that the coagulation–PAR-2 link is mechanistically actionable.

### 5.5. PAR-2 as an Inflammatory Amplifier of KRAS–ERK Output

Greater than 90% of PDACs harbour activating *KRAS* mutations, and the resulting MAPK addiction is a defining biological feature of the disease [86]. PAR-2 should not be conceptualised as an alternative driver of MAPK signalling in this context but as an inflammatory and stromal amplifier of pre-existing *KRAS*–ERK output. Mechanistically, PAR-2-driven IL-6 and IL-8 release potentiates JAK/STAT3 signalling, while PAR-2-coupled NF-κB activity sustains the cytokine milieu that supports tumour-cell plasticity [9,17]. In PDAC cells, PAR-2 activation contributes to ERK-dependent expression of urokinase plasminogen activator and matrix metalloproteinases, supporting a pro-invasive phenotype, and PAR-2 silencing reduces ERK phosphorylation and impairs Panc1 xenograft growth [85,87]. Conceptually, PAR-2 inhibition in *KRAS*-mutant PDAC may be viewed less as a strategy to suppress MAPK addiction outright than as a rational means of reducing the inflammatory pressure that sustains adaptive resistance to MEK and ERK inhibitors, a hypothesis that requires testing in genetically defined preclinical models such as KPC mice [88,89]. A mechanistic question of particular relevance to PDAC, in which activating *KRAS* mutations are present in more than 90% of tumours, is how constitutive *KRAS* signalling intersects with the lifecycle of PAR-2 itself. Although direct studies of *KRAS*-dependent PAR-2 trafficking in pancreatic cells remain limited (a relationship that is therefore best regarded as hypothesis-generating), several convergent lines of evidence indicate that oncogenic *KRAS* is positioned to prolong and amplify PAR-2 output rather than to terminate it. First, PAR-2 is an irreversibly cleaved receptor whose signal is normally extinguished by β-arrestin-dependent internalisation, ubiquitination and lysosomal degradation rather than by ligand dissociation; the cell-surface E3 ubiquitin ligases RNF43 and RNF152, which constrain PAR-2 and the related anti-apoptotic node Bcl-xL respectively, are frequently downregulated or functionally bypassed in *KRAS*-driven gastrointestinal epithelium, predicting reduced receptor turnover and sustained signalling. Second, constitutive *KRAS*–RAF–MEK–ERK flux maintains a phosphorylation environment that favours sustained ERK output downstream of PAR-2 and reinforces β-arrestin-scaffolded endosomal ERK signalling, effectively decoupling receptor internalisation from signal termination so that internalised PAR-2 continues to signal from the endosomal compartment [90]. Third, mutant *KRAS* upregulates tissue factor expression and tumour-cell procoagulant activity, increasing local generation of FVIIa and FXa and thereby sustaining the very proteolytic input that recleaves and reactivates newly trafficked PAR-2 at the surface. The net prediction is that, in *KRAS*-mutant PDAC, the balance between PAR-2 activation and inactivation is shifted towards persistent, internalisation-resistant signalling, providing a coherent rationale for why coagulation- and inflammation-driven PAR-2 output is so difficult to extinguish in this disease and reinforcing the argument that PAR-2 functions as an amplifier of pre-existing *KRAS*–ERK addiction rather than as an independent driver. Direct interrogation of *KRAS*-dependent PAR-2 internalisation kinetics using cleavage-state-specific and endosomal reporters in isogenic *KRAS*-mutant and *KRAS*-wild-type pancreatic models is an important priority [87].

#### *KRAS* and the Endocytic Itinerary of PAR-2: Internalisation Kinetics, Sorting and Receptor Replenishment

The preceding argument, that oncogenic *KRAS* is positioned to prolong PAR-2 output, requires a step-resolved account if it is to be tested rather than merely asserted. Because PAR-2 is activated by irreversible proteolysis, it cannot be switched off by ligand dissociation, and termination of signalling is therefore accomplished almost entirely by trafficking. The endocytic itinerary is, in effect, the receptor’s off-switch, which makes its kinetics a first-order determinant of signal duration and an unusually attractive point of oncogenic control [91,92]. We first set out the canonical itinerary because the *KRAS*-dependent predictions are interpretable only against it.

Within seconds to minutes of cleavage, G-protein-coupled receptor kinases phosphorylate the PAR-2 carboxy-terminus, inducing recruitment of β-arrestin-1 and β-arrestin-2, which sterically uncouple the receptor from heterotrimeric G proteins and desensitise Gαq-dependent signalling [93]. The β-arrestins then serve as adaptors for clathrin- and AP-2-mediated, dynamin-dependent endocytosis, with an internalisation half-time of the order of minutes [93]. Internalisation does not, however, terminate all signalling: β-arrestins co-internalise with the receptor and scaffold a Raf-1–MEK–ERK complex on the endosome, so that ERK1/2 activity is retained in a cytosolically sequestered pool decoupled from nuclear translocation [20]. This asymmetry is fundamental, since endocytosis terminates the calcium arm while perpetuating the MAPK arm. Activated PAR-2 is then multi-mono-ubiquitinated by the RING-domain E3 ligase c-Cbl, which is recruited to the receptor at the plasma membrane and on endosomes in a Src-dependent manner following its own tyrosine phosphorylation [94]. Ubiquitinated receptor engages the HRS–STAM complex, which is ESCRT-0, within clathrin-coated microdomains of the early endosome [93,95], and is handed to ESCRT-I, -II and -III for sorting into intraluminal vesicles, delivery to multivesicular bodies and late endosomes, and ultimately lysosomal degradation. Deubiquitination by the endosomal deubiquitinases AMSH and UBPY (USP8) between the early endosome and the lysosome is required for this sorting: catalytically inactive AMSH (D348A) or UBPY (C786S), or knockdown of either enzyme, increases steady-state receptor ubiquitination, traps PAR-2 in early endosomes and prevents lysosomal trafficking and degradation [93].

The decisive experiment for the present question was performed two decades ago and has not, to our knowledge, been revisited in an oncogenic context. A PAR-2 mutant lacking all intracellular lysine residues, PAR2Δ14K/R, and dominant-negative c-Cbl lacking the RING-finger domain both abolish receptor ubiquitination; the receptor still internalises normally but is retained in early endosomes, escapes lysosomal degradation, and, crucially, recycles to the surface and continues to signal, whereas wild-type PAR-2 is degraded and recovery of agonist responsiveness requires synthesis of new receptor [94]. The ubiquitin-dependent sorting step is therefore precisely what converts PAR-2 from a recycling receptor into a single-use one, and interrupting that single step is sufficient to create a persistently signalling, recycling receptor. Replenishment of wild-type receptor draws on two sources: de novo synthesis and mobilisation of a large pre-formed intracellular pool. Palmitoylation of PAR-2 at cysteine 361, occurring in the cis- to medial-Golgi, anchors the carboxy-terminal domain to the inner leaflet and is required both for efficient delivery from Golgi stores to the plasma membrane and for optimal signalling; palmitoylation-deficient receptor is approximately nine-fold less potent in response to trypsin, with a reduction of about a third in maximal signal, and interaction of palmitoylated PAR-2 with Rab11a promotes agonist-induced repopulation of the cell surface [96]. Three nodes are therefore tunable: the rate of internalisation, the efficiency of ubiquitin-dependent degradative sorting, and the rate of Golgi-pool replenishment.

Against this framework, oncogenic *KRAS* is predicted to act at several nodes, and the predictions are not all in the same direction, which is why the question must be settled empirically rather than by argument. At the ubiquitination node, the naive prediction is that elevated Src and focal-adhesion-kinase activity downstream of mutant *KRAS* would enhance the Src-dependent recruitment of c-Cbl and therefore accelerate degradation. We propose the opposite and we suggest a more probable prediction: c-Cbl is the shared E3 ligase for EGFR and PDGFR as well as PAR-2 [94], and *KRAS*-mutant PDAC sustains a high receptor-tyrosine-kinase load, so competitive sequestration of a limiting c-Cbl pool by hyperactive receptor tyrosine kinases would reduce PAR-2 ubiquitination and phenocopy the PAR2Δ14K/R state pharmacologically, producing early-endosomal retention, escape from lysosomal degradation, recycling and persistent signalling. This is a specific and readily falsifiable hypothesis. At the alternative-ligase node, RNF43, a Wnt-target E3 ligase that induces PAR-2 turnover in colon cancer [56], is inactivated in a proportion of pancreatic and colorectal tumours, and its loss predicts reduced receptor turnover. At the endosomal-signalling node, constitutive *KRAS*–RAF–MEK flux sustains the β-arrestin-scaffolded ERK module, so internalisation loses its terminating function [20]. At the replenishment node, the anabolic and secretory programmes driven by mutant *KRAS* predict accelerated Rab11a-dependent repopulation of the surface from the Golgi pool, and therefore faster functional resensitisation [96]. At the substrate-supply node, *KRAS*-driven upregulation of tissue factor sustains local FVIIa and FXa generation and therefore recleaves newly delivered receptor [87]; this is an input effect rather than a trafficking effect, and the distinction matters because it means persistent signalling can arise with an entirely normal itinerary. Against these, one node predicts the opposite: *KRAS*-mutant PDAC exhibits constitutive macropinocytosis and enhanced autophagic and lysosomal flux, which would increase bulk membrane turnover and lysosomal degradative capacity and could accelerate rather than retard PAR-2 clearance. The net effect is therefore an empirical question, and the balance of predictions, while favouring prolonged signalling, is explicitly labelled hypothesis-generating (Table 2).

The experimental programme required to resolve this is specific and feasible. An isogenic system is essential: *KRAS*-wild-type and *KRAS*-G12D-expressing pancreatic epithelial cells or patient-derived organoids, ideally complemented by a *KRAS*-G12C model in which the oncogene can be acutely inhibited with a covalent inhibitor to separate acute from adapted states. Internalisation kinetics should be measured by reversible surface biotinylation pulse-chase or by a surface-exposed HiBiT tag, reporting the internalisation half-time rather than a single endpoint. β-arrestin recruitment amplitude and decay should be measured by bioluminescence resonance energy transfer or NanoBiT complementation. Ubiquitination should be assessed by receptor immunoprecipitation and ubiquitin immunoblotting with proteasomal and lysosomal inhibitor controls, together with c-Cbl co-immunoprecipitation and Src inhibition as a mechanistic control, and with HRS or UBPY knockdown as positive controls for sorting arrest [93,95]. Post-endocytic itinerary should be tracked by colocalisation with Rab5, Rab7, Rab11 and LAMP1, with the prediction that *KRAS*-mutant cells show increased Rab11-positive recycling and delayed LAMP1 delivery. Degradation should be quantified by cycloheximide chase. Replenishment should be isolated using brefeldin A to block Golgi exit and 2-bromopalmitate to block palmitoylation [96], with recovery of trypsin-evoked calcium transients as the functional readout of resensitisation. Finally, because cleavage site determines trafficking fate, the entire panel should be run with trypsin, FXa and neutrophil elastase in parallel, given that the elastase-cleaved receptor is not ubiquitinated [93]. The composite prediction to be falsified is that *KRAS*-mutant cells will show an unchanged internalisation half-time but reduced receptor ubiquitination, increased recycling, delayed lysosomal delivery, prolonged endosomal ERK activity and faster functional resensitisation.

### 5.6. PAR-2, Cancer-Associated Fibroblasts and Desmoplasia

CAFs constitute the dominant cellular component of the PDAC stroma and exist as functionally heterogeneous populations of myofibroblastic (myCAF), inflammatory (iCAF) and antigen-presenting (apCAF) subtypes [97]. Pancreatic stellate cells, the principal CAF precursors, are activated by tumour-derived TGF-β and PDGF and respond to a range of serine-protease cues [98]. PAR-2 expression in stromal fibroblasts couples extracellular protease activity to fibroblast contractility, ECM deposition and pro-inflammatory cytokine secretion, suggesting that the receptor is well placed to drive iCAF-skewed phenotypes that produce IL-6 and CXCL1 and feed back onto tumour cells [99,100]. The therapeutic complexity of stromal targeting in PDAC, where wholesale stromal depletion has accelerated rather than restrained tumour progression in some genetic models [101], argues for selective modulation rather than ablation of stromal signalling; the PAR-2 axis is a plausible candidate node for such selective intervention.

### 5.7. PAR-2 in Perineural Invasion, Pain, and Neuroimmune Signalling

Perineural invasion is a near-universal feature of PDAC and is mechanistically linked both to debilitating pain and to local recurrence [73]. PAR-2 occupies a uniquely double-edged position in this domain: it is a well-characterised driver of nociceptor sensitisation, with primary afferent neurons responding to PAR-2 activation through TRPV1, TRPV4 and TRPA1 sensitisation and release of neurokinins [102,103], while at the same time, tumour cell PAR-2 contributes to invasion and stromal remodelling [104]. This duality raises the prospect that PAR-2-targeted therapeutics might simultaneously address tumour invasion, neural plasticity and cancer pain, three of the most clinically pressing problems in PDAC. The challenge for translation is that effective and selective PAR-2 antagonists with favourable pharmacokinetics are still in development [105,106]; in the interim, indirect approaches that suppress upstream protease activity (statins, FXa inhibitors, nutraceuticals) may offer a more immediately testable route.

### 5.8. PAR-2 and Chemoresistance in PDAC

Gemcitabine- and FOLFIRINOX-based therapy remains the cytotoxic backbone of PDAC management, and resistance arises through a constellation of mechanisms that include impaired drug delivery, stromal protection, anti-apoptotic buffering and metabolic adaptation [107,108]. Several of these mechanisms intersect with PAR-2 biology. Anti-apoptotic Bcl-xL stabilisation, demonstrated as a tumour-intrinsic PAR-2 mechanism in CRC [22], is a plausible analogue in PDAC, where Bcl-xL is widely upregulated and contributes to gemcitabine tolerance. PAR-2-coupled NF-κB signalling supports the stromal and inflammatory milieu that protects tumour cells from cytotoxic insult [109,110,111], while TF–FVIIa/FXa-PAR-2-driven angiogenesis and pericyte recruitment may compromise drug penetration through the desmoplastic barrier. Although direct evidence that PAR-2 antagonism sensitises PDAC to chemotherapy is still fragmentary, the integrative mechanistic case for testing combinations of PAR-2 modulators with gemcitabine and platinum-based regimens is strong, particularly in the high-stromal, high-tissue factor subset of patients. We note explicitly that a direct demonstration that PAR-2 antagonism sensitises PDAC to chemotherapy is not yet available; the proposition is therefore hypothesis-generating, resting on directly demonstrated CRC analogues and on indirect PDAC evidence [112].

## 6. Comparative Biology of PAR-2 Signalling in CRC and PDAC

Although CRC and PDAC are both gastrointestinal epithelial tumours that depend on *KRAS*- or *KRAS*-pathway-driven MAPK signalling for proliferation and survival, they engage PAR-2 through structurally and functionally distinct microenvironments. CRC arises within an epithelial compartment continuously exposed to microbial proteases and luminal inflammatory mediators, and PAR-2 in this setting integrates barrier dysfunction with mucosal immune activation [12,113]. PDAC, by contrast, evolves within a closed, fibrotic, coagulation-activated microenvironment in which tissue factor and pancreatic proteases dominate the proteolytic landscape [66]. The downstream consequences of PAR-2 activation diverge accordingly: in CRC, NF-κB- and Wnt/β-catenin-coupled inflammatory programmes and Bcl-xL-dependent therapy resistance predominate; in PDAC, TGF-β/ALK5-dependent motility, ATP-purinergic cross-talk and TF-coagulation-driven angiogenesis are the most prominent outputs [22,23,27,48,50,81]. The translational corollary is that PAR-2-directed strategies must be tailored to disease context: EGFR-therapy sensitisation and biomarker-stratified immunotherapy combinations are the most attractive avenues in CRC, whereas anticoagulant repurposing, stromal modulation and combined antitumour–anti-pain strategies are more compelling in PDAC (Table 3).

## 7. PAR-2 and the Gastrointestinal Tumour Microenvironment

### 7.1. Tumour Epithelial Cells

Within tumour epithelial cells, PAR-2 expression has been reported in the great majority of CRC and PDAC cell lines and primary tumours, with cell-surface receptor density modulated by inflammatory cytokines, hypoxia and TGF-β [5,48]. Tumour-cell-intrinsic PAR-2 activation drives ERK/MAPK, NF-κB, AKT, Wnt/β-catenin and Rho-GTPase signalling and supports proliferation, survival, EMT and migration [23,48,114]. Importantly, tumour-cell PAR-2 may also engage in homotypic cross-talk with stromal and immune compartments through release of cytokines (IL-6, IL-8), chemokines (CXCL1) and ATP, generating paracrine signals that propagate proteolytic information across the tumour ecosystem [48,81].

A further dimension of tumour-cell PAR-2 biology that has only recently come into focus is its intersection with extracellular-vesicle (EV) trafficking, including the large oncosomes shed by migratory carcinoma cells. This relationship is presently hypothesis-generating in the specific context of PAR-2, but the components are individually well established. Tumour-derived EVs and oncosomes are enriched in tissue factor and in active serine proteases (matriptase and trypsin-like activities), and can therefore act as mobile, membrane-bound platforms that deliver PAR-2-activating proteolytic activity to distant epithelial, stromal and endothelial cells, extending coagulation- and protease-driven PAR-2 signalling beyond the immediate cell of origin and potentially contributing to pre-metastatic niche conditioning. Conversely, PAR-2 activation engages RhoA–ROCK and Rac1-dependent actomyosin and membrane-blebbing programmes that drive amoeboid migration and large-oncosome biogenesis, so PAR-2 may both respond to and promote EV release. Integrating EV tissue factor and EV-associated protease activity into the protease–PAR-2 framework is therefore an attractive avenue, and EV cargo may itself constitute a liquid-biopsy readout of systemic protease–PAR-2 activity that complements the circulating activation-fragment assays discussed in Section 11 [115,116].

### 7.2. Cancer-Associated Fibroblasts and Stromal Remodelling

CAF-expressed PAR-2 contributes to fibroblast activation, ECM deposition and IL-6 secretion, and fibroblast-derived proteases such as MMP-9 in turn modulate tumour-cell PAR-2 cleavage [117,118,119]. The reciprocity of these interactions is particularly evident in PDAC, where pancreatic stellate-cell-derived CAFs build a dense desmoplastic compartment that both restrains drug delivery and enhances it [120,121,122]. Selective targeting of PAR-2 on CAFs rather than wholesale stromal depletion offers a path to constraining the fibroinflammatory loop without losing the protective stromal architecture that limits unrestricted invasion. The compartment-specific CAF PAR-2 effects described here are largely supported by indirect evidence, since most data derive from tumour-cell or mixed-population systems rather than from fibroblast-restricted genetic models [123].

#### 7.2.1. ECM Stiffness, Mechanotransduction and the Protease–PAR-2 Axis

The preceding discussion of CAF activation and ECM deposition addresses the biochemical arm of stromal remodelling but does not fully capture its biomechanical dimension, which is increasingly recognised as a determinant of invasion and therapy resistance in both CRC and, especially, PDAC [124,125]. The desmoplastic PDAC stroma is among the stiffest of human tumour matrices, and elevated matrix stiffness, solid stress and aligned collagen architecture are transduced into tumour and stromal cells through integrin-clustered focal adhesions, focal adhesion kinase (FAK) activation, Rho/ROCK-dependent actomyosin contractility and nuclear translocation of the mechanosensitive transcriptional coactivators YAP and TAZ [82,124,125,126]. There is a strong mechanistic rationale, although the specific intersection with PAR-2 remains largely hypothesis-generating, for reciprocal coupling between this mechanotransduction circuitry and protease–PAR-2 signalling. PAR-2 couples through Gα12/13 to RhoA–ROCK, the same contractility axis that CAFs use to stiffen and remodel the matrix, so PAR-2 activation is positioned to increase cell-intrinsic tension and matrix cross-linking, while the resulting stiffer matrix in turn promotes integrin–FAK–YAP/TAZ signalling that can upregulate the expression of PAR-2-activating proteases (matriptase, MMPs) and of tissue factor, closing a feed-forward mechanochemical loop [124,126]. YAP/TAZ activation additionally sustains a CAF-secretory phenotype and supports the iCAF cytokine output (IL-6, CXCL1) that feeds back onto tumour-cell PAR-2 [126]. Functionally, this convergence predicts that protease–PAR-2 signalling and matrix mechanics act synergistically to drive invasion at the tumour–stroma interface and to exclude both drugs and effector immune cells; it implies that a PAR-2-directed or upstream-protease-directed intervention might partially normalise stromal mechanics by interrupting the RhoA–ROCK–YAP/TAZ contractility loop [125,126]. This mechanobiological dimension is developed further, in the specific context of stromal drug resistance, in Section 9.5.

#### 7.2.2. Molecular Interfaces Between PAR-2 Signalling and Mechanotransduction in the Desmoplastic Stroma

The feed-forward scheme outlined above establishes that protease–PAR-2 signalling and matrix mechanics are coupled, but it does not specify where the two systems actually touch. Because the reviewer’s question turns on that specification, we identify four candidate molecular interfaces, each of which is composed of two arms that are individually established and a junction that is not, and each of which yields a falsifiable prediction.

The first interface is convergent calcium. Pancreatic stellate cells, the precursors of the pancreatic cancer-associated fibroblast, undergo durotaxis, migrating up stiffness gradients in a manner orchestrated by the mechanosensitive channel Piezo1 and amplified by TRPV4 and TRPC1, with optimal durotaxis requiring an intermediate level of channel activity [127]. All three channels are calcium permeable, and calcium is the second messenger through which a local mechanical event is converted into a global cellular response. PAR-2 signals through Gαq and phospholipase C to inositol trisphosphate-mediated release of endoplasmic reticulum calcium and store-operated calcium entry (Section 3.1). The two systems therefore converge on the same second messenger, in the same cell type, in the same tissue. The prediction that follows is specific and untested: PAR-2 activation should lower the mechanical threshold for Piezo1-dependent responses by pre-loading the cytosolic calcium signal, and conversely, stiffness-evoked calcium entry should summate with protease-evoked calcium release, so that protease-rich and mechanically stiff environments are not merely correlated but functionally synergistic. Both arms are additionally pH-sensitive, since Piezo1 function in pancreatic stellate cells is impaired by protonation and PAR-2 agonist potency varies with pH [128], so the acidosis of the desmoplastic microenvironment is a shared modifier rather than an independent variable.

The second interface is the Gα12/13–RhoA–ROCK actomyosin module, and it is the strongest of the four because it is not a point of convergence but a point of identity. PAR-2 couples through Gα12/13 to RhoGEFs, including p115-RhoGEF and LARG, activating RhoA and then ROCK, which increases myosin light-chain phosphorylation both directly and by inhibiting myosin light-chain phosphatase. This is the identical module through which cells generate the traction that stiffens and aligns a collagen matrix. PAR-2 is therefore not merely a receiver of mechanical information; it is a bona fide input to the machinery that manufactures it. The downstream consequences are correspondingly concrete: increased traction across integrin adhesions promotes talin unfolding and vinculin recruitment, reinforcing the adhesion and driving focal-adhesion-kinase autophosphorylation at tyrosine 397 and subsequent Src recruitment, while sustained contractility supports lysyl-oxidase-dependent collagen cross-linking and fibre alignment. Because RhoA requires geranylgeranylation for membrane localisation, this interface makes a direct and clinically tractable prediction that connects to Section 10.3: statin-mediated depletion of geranylgeranyl pyrophosphate should attenuate the mechanochemical arm of PAR-2 signalling preferentially, and geranylgeranyl pyrophosphate add-back should rescue it.

The third interface is YAP and TAZ. Nuclear translocation of these coactivators is the canonical readout of matrix stiffness, but they are also canonical effectors of Gα12/13–RhoA signalling, since receptors coupling to Gα12/13 activate YAP through RhoA-dependent inhibition of LATS1/2. PAR-2 is therefore positioned to activate YAP and TAZ independently of matrix stiffness by a purely biochemical route. This yields the most discriminating prediction available in this area: on a compliant substrate of fixed low stiffness, protease-driven PAR-2 activation should still drive nuclear YAP accumulation, and conversely, PAR-2 blockade should reduce nuclear YAP at fixed stiffness. Nuclear YAP and TAZ in turn sustain the inflammatory cancer-associated fibroblast secretory programme, including interleukin-6 and CXCL1, and upregulate matrix and protease genes, which closes the loop.

The fourth interface is the mechanosensitivity of protease supply itself. Stiffness- and YAP-driven transcription upregulates matriptase, matrix metalloproteinases and tissue factor, increasing the proteolytic input to PAR-2, while PAR-2-driven contractility increases the stiffness that drives that transcription. The resulting circuit has two chemical arms and two mechanical arms and is formally a positive feedback loop, which is consistent with the clinical observation that desmoplasia is progressive rather than self-limiting. Solid stress and raised interstitial fluid pressure compress vessels, producing hypoxia, impaired drug delivery and acidosis, and acidosis feeds back on both Piezo1 and PAR-2 pharmacology, adding a slower outer loop to the circuit.

These interfaces are testable with existing methods, and we specify the experiment because the value of the proposal lies in its falsifiability. Pancreatic stellate cells, cancer-associated fibroblasts and PDAC organoids should be cultured on polyacrylamide or polyethylene-glycol hydrogels tuned across a physiological to desmoplastic range of approximately 1 to 30 kilopascals, with atomic force microscopy confirmation. Readouts should include traction force microscopy, the nuclear to cytoplasmic ratio of YAP, phosphorylated myosin light chain, focal-adhesion-kinase phosphorylation at tyrosine 397 and live-cell calcium imaging. Interventions should include a PAR-2 negative allosteric modulator such as I-117 [128], the ROCK inhibitor Y-27632, the Gαq inhibitor YM-254890, Piezo1 blockade with GsMTx4 or genetic deletion, lysyl-oxidase inhibition, and a statin with geranylgeranyl pyrophosphate rescue. The principal falsifiable predictions are that PAR-2 antagonism will reduce nuclear YAP and traction force on stiff but not compliant substrates, and that Piezo1 loss will blunt stiffness-dependent but not protease-dependent PAR-2 output; if PAR-2 blockade fails to reduce traction at any stiffness, or if the two inputs prove strictly additive rather than synergistic, the model proposed here is wrong. The therapeutic rationale that would follow from confirmation is mechanical normalisation of the stroma without stromal ablation, which Section 9.5 argues is the only defensible stromal strategy in PDAC. Each arm of each interface is individually established; the junctions are hypothesis-generating.

### 7.3. Endothelial Activation and Angiogenesis

Endothelial PAR-2 contributes to vascular permeability, leukocyte adhesion, and angiogenic remodelling. Tissue factor–FVIIa-mediated PAR-2 activation drives expression of IL-8, CXCL1 and VEGF in endothelial cells [17,81,100,129], linking the coagulation–PAR-2 axis to neoangiogenesis and to the formation of the metastatic niche. In *Apc*^Min/+^ intestinal tumours, PAR-2 deficiency reduced microvessel density alongside attenuated NF-κB activity [50], consistent with a coupled inflammatory–angiogenic role for the receptor in CRC.

### 7.4. Mast Cells and the Tryptase–PAR-2 Axis

Mast-cell-derived tryptase is among the most potent physiological activators of PAR-2, and high mast-cell density correlates with poor prognosis in CRC [5,130]. Tryptase activation of tumour-cell PAR-2 drives MYO10-dependent migration in CRC cells, indicating a direct mast-cell–PAR-2 vector for invasion [58]. The tryptase–PAR-2 axis also contributes to angiogenesis, neurogenic inflammation, and fibroblast activation, and inhibitors of mast-cell degranulation or selective tryptase blockade therefore represent a complementary therapeutic strategy to direct PAR-2 antagonism.

### 7.5. Neutrophils, NETs and Proteolytic Inflammation

Tumour-associated neutrophils release elastase, cathepsin G and proteinase 3, and form neutrophil extracellular traps (NETs) in the metastatic niche [131,132]. Although the direct contribution of these enzymes to PAR-2 cleavage is less well defined than that of tryptase or coagulation proteases, all are biochemically capable of generating signalling-competent PAR-2 N-terminal fragments, and the high local concentrations achieved within NETs make this interaction physiologically plausible. PAR-2 activation in this setting is particularly relevant to liver metastasis from CRC and to inflammatory amplification at sites of vascular invasion in PDAC [34,133,134].

### 7.6. Macrophages and Immune Remodelling

Tumour-associated macrophages express PAR-2 and respond to receptor activation with shifts in IL-6, IL-10 and TGF-β output that contribute to M2-like polarisation in some contexts and to inflammatory M1-like behaviour in others [135,136,137]. The net immunomodulatory effect of PAR-2 in the tumour macrophage compartment depends on the protease source, the local cytokine milieu and the tumour stage, and is one of the principal reasons why PAR-2-directed therapy should be biomarker-guided rather than empirical. In evidence-strength terms, the tumour-promoting role of myeloid PAR-2 polarisation in gastrointestinal cancer is at present supported by indirect evidence extrapolated from adjacent inflammatory and non-gastrointestinal models rather than directly demonstrated in CRC or PDAC [138].

### 7.7. Platelets, Coagulation and Cancer-Associated Thrombosis

Platelet–tumour interactions are a fundamental component of metastatic dissemination, and the TF–FVIIa–FXa axis provides a direct mechanistic link between coagulation activation and PAR-2 signalling in both tumour and stromal compartments [81,100,139]. Cancer-associated venous thromboembolism is particularly common in PDAC and carries substantial morbidity [81,100,131,139]; mechanistically, the same coagulation–PAR-2 interface that drives thromboinflammation also amplifies tumour cell migration, angiogenesis and stromal activation. The cytoprotective protein C pathway, which generates activated protein C with biased PAR-1/PAR-3 signalling, exemplifies the broader principle that the coagulation–PAR network is biologically reversible and therefore therapeutically modulable [140]. Direct oral FXa inhibitors are the most clinically advanced agents capable of attenuating coagulation-driven PAR-2 signalling, and emerging preclinical evidence supports antitumour effects in colorectal models in vitro and in vivo [49,84].

## 8. The Microbiome–Barrier–PAR-2 Axis in Colorectal Carcinogenesis

### 8.1. Microbial Proteases as PAR-2 Activators

The gut microbiome is increasingly understood as an integral participant in colorectal carcinogenesis, with specific taxa enriched at successive stages of the adenoma–carcinoma sequence [141,142,143]. A subset of luminal commensals secretes serine and cysteine proteases that are biochemically competent to activate PAR-2 on host epithelium. The most rigorously characterised example is the *Bacteroides fragilis* protease fragipain (Bfp1), which Lakemeyer and colleagues recently showed to activate host PAR-2 to induce intestinal pain and inflammation in vivo [12]. Multi-omic analyses of the ulcerative colitis microbiome have similarly linked *Bacteroides vulgatus* proteases to disease severity [6], while luminal cysteine-protease activity has been mechanistically connected to tight-junction degradation and visceral hyperalgesia in irritable bowel syndrome and to colonic barrier dysfunction more generally [144,145]. Together, these observations frame microbial protease–PAR-2 signalling as a tractable axis in inflammation-driven CRC and identify the activated PAR-2 N-terminal fragment as a candidate biomarker of luminal proteolytic burden.

### 8.2. PAR-2, Epithelial Permeability and Chronic Mucosal Injury

PAR-2 activation modulates the integrity of intestinal tight junctions through Rho-GTPase- and ERK-dependent reorganisation of zonula occludens proteins, with consequent increases in paracellular permeability and luminal-to-mucosal antigen translocation [146,147,148]. In the context of chronic mucosal injury, this barrier disruption establishes a self-sustaining cycle of microbial translocation, innate immune activation and epithelial regeneration that is permissive for adenomatous transformation. Animal models in which PAR-2 has been genetically deleted display attenuated intestinal tumour formation and reduced inflammatory cytokine output, providing in vivo evidence that the microbiome–barrier–PAR-2 axis is causally implicated in colorectal carcinogenesis [50,149].

### 8.3. The Microbiome–PAR-2–Immune Axis

Beyond barrier function, microbial protease signalling through PAR-2 shapes mucosal immunity. PAR-2 activation on dendritic cells and macrophages modulates IL-23 and IL-17 axis activity, which has been implicated in colitis-associated CRC [150,151,152]. Comparative analyses of duodenal microbial communities have shown that microbial protease activity can either potentiate or suppress mucosal immunogenicity depending on the dominant taxa [153], supporting the view that the microbiome–PAR-2 interface is highly context-dependent and amenable to therapeutic intervention through selective modulation of the proteolytic component of the microbial output.

### 8.4. Translational Opportunities

The microbiome–barrier–PAR-2 axis suggests several immediately testable translational strategies. Faecal protease activity can be quantified in routine clinical samples and could serve as a stratification tool for inflammation-driven CRC; activated PAR-2 fragments measured in serum (PRO-PAR2) provide a complementary systemic readout [154,155]. Probiotic, postbiotic and dietary interventions that suppress the proteolytic component of the microbiome may attenuate PAR-2-mediated barrier injury without the systemic consequences of receptor blockade [156], and selective protease inhibitors (e.g., matriptase- or tryptase-specific) could in principle be deployed in inflammation-prone or IBD-associated CRC. Nutraceutical agents such as oleocanthal and curcumin, which we have shown to modulate PAR-2-driven inflammatory output in CRC cell models [25,26], may exert part of their effect through dampening this microbiome-barrier-receptor axis—an interpretation that warrants direct testing in microbiota-defined preclinical systems.

## 9. PAR-2 and Therapy Resistance

### 9.1. Cytotoxic Chemotherapy Resistance

PAR-2 contributes to cytotoxic chemotherapy resistance through multiple, partially overlapping mechanisms. This is supported by doxorubicin-focused evidence in HT-29 colon adenocarcinoma cells showing that PAR-2 activation by trypsin or 2-furoyl-LIGRLO-NH_2_ reduces doxorubicin-induced cell death, reactive oxygen species generation and caspase-3/7 activation through MEK1/2–ERK1/2-dependent upregulation of the anti-apoptotic proteins MCL-1 and Bcl-xL, whereas PAR-2 deletion or pharmacological antagonism restores doxorubicin sensitivity [157]. PAR-2-coupled AKT and ERK activation supports anti-apoptotic signalling and the maintenance of mitochondrial integrity under cytotoxic stress, while NF-κB-driven cytokine output remodels the tumour microenvironment to a chemoprotective state [9,158,159]. In CRC cell lines, PAR-2 inhibition with ENMD-547 enhanced 5-fluorouracil efficacy and reduced EMT markers [53], and our recent work has shown that statins, through selective downregulation of PAR-2 expression and PAR-2-driven calcium and TNF-α responses, can convert inflammatory survival signalling into apoptosis in CRC cells [24]. The convergence of these observations supports the view that PAR-2 modulation should be tested as an adjunct to standard cytotoxic regimens, particularly in inflammation-rich tumour subtypes.

### 9.2. EGFR-Targeted Therapy Resistance in CRC: The Bcl-xL/RNF152 Axis

The most mechanistically defined link between PAR-2 and targeted-therapy resistance comes from CRC. Li et al. demonstrated that PAR-2 activation phosphorylates Bcl-xL at Ser-145, displaces the E3 ligase RNF152 from the Bcl-xL/RNF152 complex and thereby stabilises Bcl-xL, conferring resistance to multiple EGFR-targeted therapies in CRC patient-derived xenografts; PAR-2 inhibition restored RNF152-dependent Bcl-xL ubiquitination and re-sensitised the tumours to cetuximab [22]. This finding has several important implications. First, it implies that PAR-2 inhibition is most likely to be clinically useful in RAS/RAF-wild-type CRC, where downstream pathway rewiring is not the dominant resistance mechanism. Second, it justifies the combination of PAR-2 antagonists or PAR-2-suppressive agents (such as statins) with anti-EGFR antibodies in defined biomarker-positive populations. Third, it nominates phosphorylated Bcl-xL and tumoural PAR-2 expression as candidate companion biomarkers.

### 9.3. MAPK-Pathway-Inhibitor Resistance

Adaptive resistance to MEK and ERK inhibitors is a major obstacle in *KRAS* and *BRAF*-driven gastrointestinal tumours. PAR-2-coupled inflammatory transcription and EGFR transactivation may participate in such resistance by sustaining low-level ERK signalling and by promoting compensatory cytokine release [19,21,49]. A rational hypothesis is that combined inhibition of PAR-2 (or its upstream proteolytic activators) with MEK or ERK inhibitors will reduce the inflammatory pressure that drives adaptive pathway reactivation, an approach that warrants direct testing in *BRAF*-mutant CRC models and in *KRAS*-mutant PDAC. This strategy is clinically plausible because several MEK1/2 inhibitors are already approved in oncology or MAPK-driven disease settings, including trametinib, cobimetinib, binimetinib and selumetinib; by contrast, direct ERK1/2 inhibitors such as ulixertinib/BVD-523 remain investigational and are being evaluated through clinical trials or expanded-access programmes rather than routine approval [160].

### 9.4. Immunotherapy Resistance and the PAR-2 Paradox

The most striking nuance in PAR-2 oncology is the recent recognition that tumour-intrinsic PAR-2 may be required for productive antitumour immunity in certain settings. As outlined above, evidence from inflammation-prone colon cancer models suggests that loss of tumour-intrinsic PAR-2 can attenuate CD8^+^ T-cell function and reduce checkpoint-inhibitor efficacy [8,26], while PAR-2-coupled inflammatory transcription and stromal remodelling clearly support tumour progression in other contexts. The translational corollary is that PAR-2 modulation in immunotherapy-eligible patients, particularly those with mismatch-repair-deficient or microsatellite-instability-high CRC, must be considered with caution, ideally after immune-context profiling. In immune-cold, stroma-rich tumours such as the bulk of PDAC, by contrast, PAR-2 inhibition is more likely to act as an immunological sensitiser by reducing fibroinflammatory immune exclusion [161,162]. A case in point is the TF–FVIIa–FXa-rich PDAC microenvironment, in which PAR-2 signalling links coagulation activation to CAF activation, angiogenic cytokine release, TGF-β-driven invasion and exclusionary stromal architecture; interrupting this axis could therefore convert PAR-2 blockade from a purely anti-invasive strategy into a microenvironment-normalising adjunct to chemotherapy and immunotherapy [27,29,163].

### 9.5. Stromal Resistance in PDAC

In PDAC, therapy resistance is driven as much by the desmoplastic stroma as by tumour-cell-intrinsic alterations. The stroma generates physical barriers to drug penetration, hypoxic adaptation and CAF-mediated cytokine support that together blunt the impact of cytotoxic and targeted regimens [86,99,120,121,164,165,166]. The PAR-2 axis is implicated at multiple points; TF–FXa–PAR-2 signalling drives angiogenic and inflammatory cytokine output that supports drug exclusion [81,86,99,120,121,132,164,165,166]; CAF PAR-2 sustains the iCAF phenotype that secretes IL-6 and CXCL1 [57,58]; and tumour-cell PAR-2 contributes to TGF-β-dependent motility that underlies invasion at the stromal interface [9,10,31]. Selective PAR-2 modulation, layered onto standard chemotherapy, is therefore a rational strategy that avoids the pitfalls of wholesale stromal depletion.

#### Three Mechanistically Distinct Arms of Stromal Resistance

To make the therapeutic logic explicit, it is useful to separate the desmoplastic contribution to PDAC therapy resistance into three mechanistically distinct but interacting arms, only the last of which is directly PAR-2-dependent. The first is a physical transport barrier: dense, cross-linked extracellular matrix and the resulting elevated interstitial fluid pressure collapse intratumoural vasculature and impede the convective and diffusive delivery of gemcitabine, nab-paclitaxel and FOLFIRINOX components to tumour cells (directly demonstrated in multiple PDAC models) [167,168]. The second is a mechanobiological signalling arm: a stiff, aligned matrix and CAF-generated contractility drive integrin–FAK, RhoA–ROCK and YAP/TAZ signalling that promotes a survival-competent, quiescent-to-invasive cell state and reinforces stromal secretion, independently of drug concentration (supported by indirect evidence in PDAC and strong direct evidence in other stiff-matrix tumours). The third is a protease-dependent, PAR-2-activated arm: tissue factor–FVIIa–FXa, mast-cell tryptase, neutrophil elastase and CAF-derived proteases converge on PAR-2 to sustain NF-κB- and ERK/AKT-driven survival signalling, IL-6/CXCL1 cytokine support and TGF-β/ALK5-dependent motility at the invasive front (directly demonstrated for individual proteases; the integrated PDAC-specific circuit is supported by indirect evidence) [169,170]. Crucially, these arms are not additive but reinforcing: PAR-2-driven RhoA–ROCK contractility feeds the mechanobiological arm, mechanotransduction upregulates proteases and tissue factor that feed the protease arm, and matrix deposition that feeds the physical-barrier arm is itself a CAF output amplified by PAR-2. This framing clarifies why wholesale stromal depletion has paradoxically accelerated tumour progression in some genetic models, since it indiscriminately removes the protective physical-barrier arm, and why selective modulation of the protease–PAR-2 node is mechanistically preferable: it can attenuate the signalling and mechanobiological arms that promote resistance while leaving the architectural stroma that restrains unrestricted invasion comparatively intact [171].

## 10. Therapeutic Targeting of the Protease–PAR-2 Axis

### 10.1. Direct PAR-2 Antagonists and Negative Allosteric Modulators

**Figure 7:**
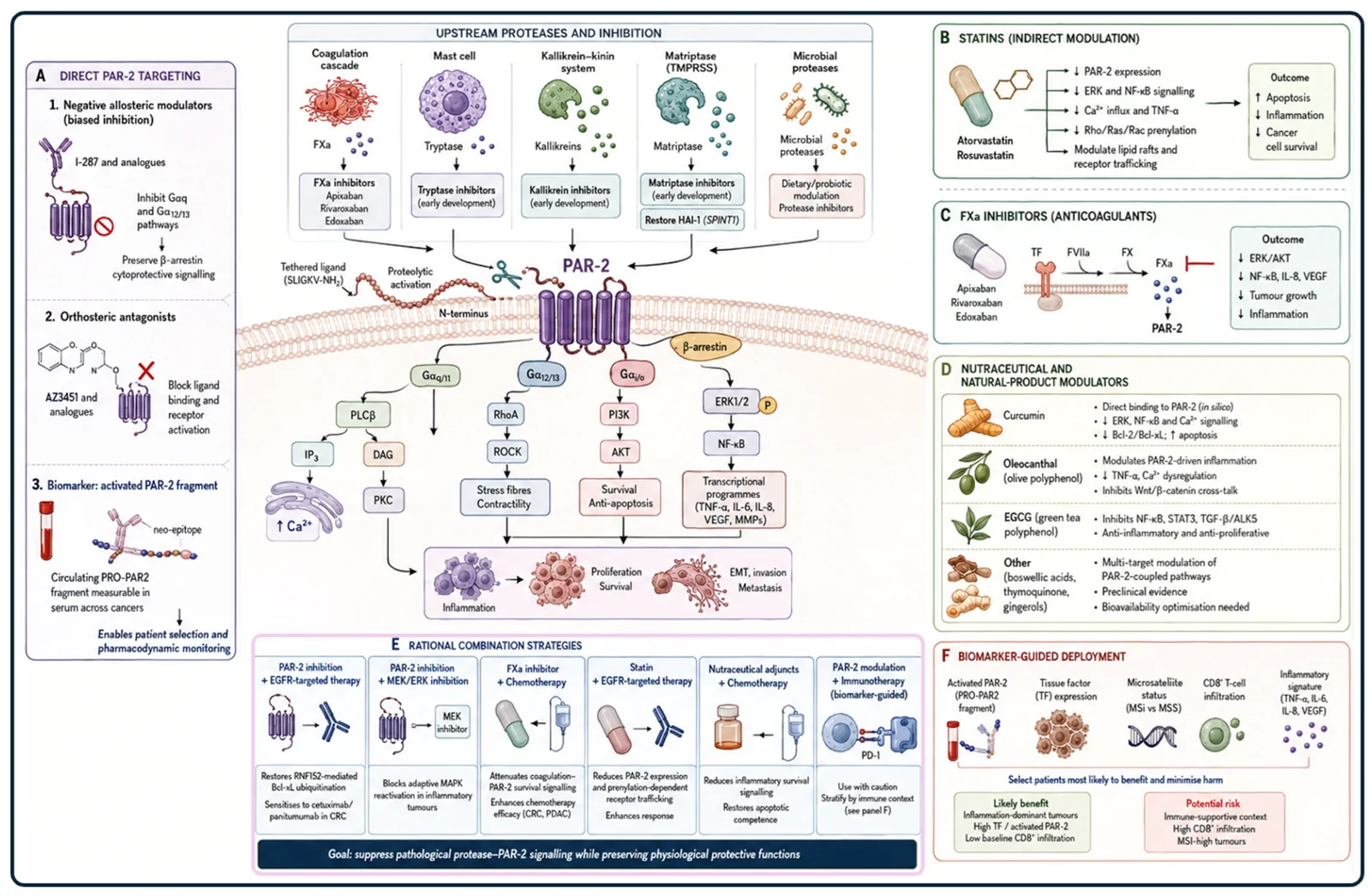
Therapeutic targeting of the protease–PAR-2 axis in colorectal and pancreatic cancer. The figure summarises therapeutic strategies for modulating the protease–protease-activated receptor-2 (PAR-2/*F2RL1*) signalling axis in colorectal cancer (CRC) and pancreatic ductal adenocarcinoma (PDAC). PAR-2 is depicted as a central seven-transmembrane GPCR activated by proteolytic cleavage of its extracellular N-terminus, exposing the tethered ligand sequence SLIGKV-NH_2_ and enabling downstream signalling through Gαq/11, Gα12/13, Gαi/o and β-arrestin-dependent pathways. These pathways regulate calcium mobilisation, RhoA–ROCK signalling, PI3K–AKT survival signalling, ERK1/2 and NF-κB activation, ultimately promoting inflammation, proliferation, survival, EMT, invasion and metastatic progression. (**A**) Direct PAR-2 targeting. Direct receptor-directed strategies include biased negative allosteric modulators, exemplified by I-287 and related analogues, which selectively inhibit Gαq and Gα12/13 signalling while preserving β-arrestin-associated cytoprotective signalling. Orthosteric or allosteric antagonistic chemotypes, including AZ3451-related compounds, interfere with ligand engagement and receptor activation. Activated PAR-2 fragment detection, such as circulating PRO-PAR2 neo-epitope assays, is shown as a potential biomarker strategy for patient selection and pharmacodynamic monitoring. (**B**) Statins as indirect PAR-2-axis modulators. Atorvastatin and rosuvastatin are shown as clinically deployable repurposed agents that indirectly attenuate pathological PAR-2 signalling. Through HMG-CoA reductase inhibition, statins reduce mevalonate-pathway-dependent prenylation of small GTPases such as Rho, Ras and Rac, alter lipid-raft-dependent receptor trafficking, suppress PAR-2 expression and reduce downstream ERK/NF-κB signalling, calcium influx and TNF-α release. These effects collectively favour apoptosis and reduce inflammatory cancer-cell survival. (**C**) Direct oral FXa inhibitors. Apixaban, rivaroxaban and edoxaban are presented as pharmacologically mature agents capable of interrupting the coagulation arm of the PAR-2 axis. By inhibiting FXa, these drugs reduce TF–FVIIa–FXa-driven PAR-2 activation and downstream ERK/AKT, NF-κB, IL-8 and VEGF signalling. This strategy is particularly relevant to TF-high, coagulation-activated tumour microenvironments, including PDAC and metastatic CRC, although clinical deployment must account for bleeding risk. (**D**) Nutraceutical and natural-product modulators. Curcumin, oleocanthal, EGCG and related bioactives are illustrated as multi-target modulators of PAR-2-coupled inflammatory and survival pathways. Curcumin is proposed to suppress PAR-2/ERK/NF-κB signalling and promote apoptotic reactivation; oleocanthal attenuates PAR-2-driven inflammatory calcium and TNF-α responses; EGCG and related polyphenols may modulate NF-κB, STAT3 and TGF-β/ALK5-linked programmes. These agents are attractive adjuncts for chemoprevention or combination therapy, although bioavailability, formulation and standardisation remain major translational limitations. (**E**) Rational combination strategies. The figure outlines combination approaches based on pathway logic. PAR-2 inhibition may restore RNF152-mediated Bcl-xL ubiquitination, thereby sensitising CRC to EGFR-targeted antibodies such as cetuximab or panitumumab. PAR-2 inhibition combined with MEK/ERK blockade may suppress adaptive MAPK reactivation in inflammatory tumours. FXa inhibitors may attenuate coagulation-driven PAR-2 survival signalling and enhance chemotherapy efficacy, particularly in PDAC. Statins may be combined with EGFR-targeted therapy to reduce PAR-2 expression, receptor trafficking and prenylation-dependent signalling, while nutraceutical adjuncts may reduce inflammatory survival programmes and restore apoptotic competence. PAR-2 modulation in immunotherapy-treated patients should be guided by immune context, given the possibility that PAR-2 may either promote inflammation-driven tumour growth or, in selected settings, sustain immune visibility. (**F**) Biomarker-guided deployment. The proposed therapeutic framework requires patient stratification using biomarkers that reflect both PAR-2 pathway activity and tumour immune context. Candidate biomarkers include activated PAR-2 fragments such as PRO-PAR2, tissue-factor expression, FXa/coagulation activation, microsatellite instability status, CD8^+^ T-cell infiltration and inflammatory cytokine signatures such as TNF-α, IL-6, IL-8 and VEGF. Patients with inflammation-dominant, TF-high and activated-PAR-2-high tumours may be most likely to benefit from PAR-2-axis suppression, whereas immune-supportive contexts with high CD8^+^ T-cell infiltration or MSI-high biology may require caution to avoid impairing antitumour immunity. Arrows indicate pathway activation or therapeutic effects, blunt-ended lines indicate inhibition, dashed links indicate indirect or proposed relationships, and colours distinguish intervention classes and biological contexts. [Note: Created in BioRender. Banerjee, Y. (2026) https://BioRender.com/97szftq, accessed on 30 July 2026].

The pharmacological landscape of direct PAR-2 inhibition has matured substantially in the past decade (Figure 7). Crystallographic and cryo-EM studies of PAR-2 in complex with the orthosteric antagonist AZ3451 and with biased-agonism-relevant small molecules have provided structural templates for rational drug design [13,33]. The negative allosteric modulator I-287 selectively inhibits Gαq and Gα12/13 PAR-2 outputs without disrupting β-arrestin recruitment, generating a biased pharmacology profile that retains potential cytoprotective signalling while blocking inflammatory output [14]. A circulating PAR-2 activation fragment has been demonstrated as a feasible biomarker, and recent serum profiling has identified elevated activated PAR-2 across multiple cancer types [59], supporting biomarker-stratified deployment of PAR-2-directed therapies. Comprehensive medicinal chemistry reviews provide an integrated view of orthosteric and allosteric chemotypes and the pharmacokinetic challenges that have so far limited clinical translation [10,35].

### 10.2. Targeting Upstream Proteases

Indirect modulation of the PAR-2 axis through inhibition of upstream proteases offers a complementary therapeutic strategy with a more mature clinical evidence base (Figure 7). FVIIa- and tissue-factor-pathway-directed inhibitors, mast-cell tryptase inhibitors, kallikrein blockade and matriptase-/TMPRSS-family-directed agents are at varying stages of preclinical and early clinical development. Microbial protease inhibition, achievable through dietary, probiotic and pharmacological means, represents a particularly attractive route for inflammation-driven CRC [6,12,144,145]. HAI-1 (*Spint1*)–matriptase signalling is genetically linked to PAR-2-dependent intestinal carcinogenesis in *Apc*^Min/+^ mice [15,16], and matriptase inhibition therefore deserves systematic exploration as a PAR-2-axis-modulating intervention.

### 10.3. Statins as PAR-2-Axis Modulators

Our recent work has positioned statins as one of the most clinically actionable indirect modulators of PAR-2-driven inflammation in CRC (Figure 7). In LPS-stimulated HT-29 and Caco-2 cells, atorvastatin and rosuvastatin selectively suppressed PAR-2, but not PAR-1, expression and abrogated the corresponding TNF-α release and calcium influx, converting inflammatory survival signalling into apoptosis [24,43]. The mechanistic basis is multifactorial: HMG-CoA reductase inhibition reduces prenylation-dependent activation of small GTPases (Rho, Ras, Rac), modulates lipid-raft-resident receptor trafficking and lowers cholesterol-dependent PAR-2 signalling efficiency [44]. Statin chemoprevention has been extensively studied in CRC at the population level [172,173,174]; our work provides a precise molecular rationale for revisiting these data in inflammation-defined subsets and for combining statins with EGFR-targeted therapies, with cytotoxic chemotherapy and with nutraceutical interventions.

An important mechanistic question raised by these observations is why statins should preferentially attenuate PAR-2 rather than PAR-1, given that the two receptors share a common tethered-ligand activation logic. Although a single definitive determinant has not been established and the proposed model is therefore best regarded as supported by indirect evidence, several non-mutually exclusive mechanisms provide a coherent molecular rationale for the observed selectivity. First, the two receptors differ in their dependence on the mevalonate–isoprenoid pathway that statins inhibit. PAR-2-driven motility and inflammatory output are routed disproportionately through Gα12/13–RhoA and Rac1, small GTPases whose membrane localisation and activity require geranyl-geranylation, whereas PAR-1 signalling in epithelial cells is comparatively more reliant on Gαq– and Gαi-coupled outputs that are less prenylation-sensitive [44]. By depleting the geranylgeranyl-pyrophosphate pool, statins therefore disable a branch on which PAR-2 is preferentially dependent. Second, PAR-2 partitions strongly into cholesterol-rich lipid-raft and caveolar microdomains for efficient cleavage, tethered-ligand presentation and β-arrestin-coupled endosomal signalling [175]; statin-mediated reduction of membrane cholesterol disrupts raft integrity and thereby lowers the efficiency of PAR-2 activation and trafficking to a greater degree than that of PAR-1, which is less raft-dependent in these cells. Third, at the transcriptional level, our data show that statins reduce *F2RL1* expression and the associated DUSP6–ERK module without a corresponding fall in *F2R* (PAR-1), indicating a degree of promoter- or stability-level selectivity that may reflect differential dependence on sterol-regulated and NF-κB-linked transcriptional inputs. The convergence of a prenylation-dependent effector bias, a lipid-raft-dependent activation bias and a transcriptional bias offers a parsimonious explanation for the selective suppression of PAR-2 over PAR-1 by atorvastatin and rosuvastatin and predicts that the effect should be most pronounced for lipophilic statins with the greatest intracellular access. Formal confirmation will require side-by-side prenylation-rescue experiments (with geranyl-geranyl-pyrophosphate supplementation) and raft-disruption controls in PAR-1- and PAR-2-matched isogenic systems.

#### Molecular Basis of the PAR-2 Versus PAR-1 Selectivity of Atorvastatin and Rosuvastatin

The three mechanisms proposed above provide a coherent account, but the question deserves a fuller treatment, both because the selectivity is the most surprising feature of our data and because a fourth mechanism, arising from the divergent lifecycles of the two receptors, has not previously been considered. We set out four non-mutually exclusive determinants together with the experiments that would discriminate between them.

The first determinant is differential dependence on protein prenylation, and it can be stated with precision. Inhibition of HMG-CoA reductase depletes mevalonate and then both farnesyl pyrophosphate and geranylgeranyl pyrophosphate [175]. RhoA, Rac1 and Cdc42 are geranylgeranylated by geranylgeranyltransferase-I at their carboxy-terminal CaaX motifs, whereas Ras is farnesylated; depletion of geranylgeranyl pyrophosphate leaves RhoA cytosolically sequestered by RhoGDI and unable to engage its membrane effectors. Because PAR-2 routes its motility and inflammatory output disproportionately through Gα12/13–RhoA and Rac1 (Section 3.6), whereas epithelial PAR-1 output leans more heavily on Gαq- and Gαi-coupled pathways that are less prenylation-dependent, statins remove a limb on which PAR-2 is preferentially reliant. The discriminating experiment is an add-back: geranylgeranyl pyrophosphate supplementation should rescue statin-mediated PAR-2 suppression, whereas farnesyl pyrophosphate should not, and mevalonate should rescue both. This experiment distinguishes on-target mevalonate-pathway action from off-target effects, and it has not been performed for the PAR-2 versus PAR-1 comparison.

The second determinant is differential dependence on cholesterol-rich membrane microdomains, and here, a specific molecular anchor is available that was not previously invoked. PAR-2 is palmitoylated at cysteine 361 within the cis- to medial-Golgi, and S-palmitoylation is the principal post-translational determinant of GPCR partitioning into lipid rafts; in PAR-2, it additionally anchors the carboxy-terminal domain to the inner leaflet, probably forming the eighth helix required for effective signal transduction. Its functional weight is large: palmitoylation-deficient PAR-2 is approximately nine-fold less potent in response to trypsin, with a reduction of about a third in maximal signal, and shows impaired cell-surface expression [96]. Statin-induced perturbation of cellular lipid homeostasis and membrane cholesterol is therefore predicted to impinge on both the raft residence and the palmitoylation-dependent trafficking of PAR-2. The corollary is worth stating explicitly because it may be the deepest reason for the selectivity: since PAR-2 is destroyed rather than reused following cleavage, its surface density depends on continuous resupply from the palmitoylation-dependent Golgi–Rab11a route [176], so any intervention that perturbs that resupply route will lower PAR-2 surface density progressively. Whether PAR-1 depends on the same route to the same degree has not been established, and a differential requirement is therefore plausible but must be demonstrated head-to-head rather than assumed.

The third determinant, which we advance here as a new hypothesis, is that the two receptors are downregulated by mechanistically distinct machinery. PAR-2 is ubiquitinated by c-Cbl and requires ubiquitin, HRS and the ESCRT machinery for lysosomal sorting [93,94,95]. PAR-1, by contrast, is rapidly deubiquitinated following activation; mutation of all its cytoplasmic lysine residues does not impair its downregulation, its degradation is unaffected by depletion of HRS or Tsg101, and it instead requires sorting nexin-1. The two receptors therefore differ not only in their transducer preferences but in the identity of the machinery that disposes of them, and that machinery operates in different membrane environments with different lipid dependencies. Any intervention acting on membrane lipid order, cholesterol content or prenylation-dependent trafficking consequently has an a priori reason to affect the two receptors unequally. This proposal is hypothesis-generating and is offered because it is testable: the sorting machinery is separable by knockdown, and a statin effect that tracks the ESCRT-dependent route but not the sorting-nexin-dependent route would support it.

The fourth determinant is transcriptional, and it is the one for which we have direct evidence in our own models: statins reduce *F2RL1* expression and the associated DUSP6–ERK module without a corresponding fall in *F2R* [24,43]. A plausible molecular basis is differential dependence of the two promoters on sterol-regulated and NF-κB-linked transcriptional inputs, given that statins reduce nuclear p65 abundance and NF-κB-dependent transcription [44]. This is directly testable by SREBP-2 knockdown, by chromatin immunoprecipitation for p65 at the *F2RL1* and *F2R* promoters, and by determining whether mevalonate add-back restores *F2RL1* transcript levels.

Two pharmacological corollaries deserve comment, one of which qualifies our own interpretation. If intracellular access was the dominant variable, the lipophilic atorvastatin should outperform the hydrophilic rosuvastatin, whose cellular entry in extrahepatic tissue depends substantially on OATP-family transporters, yet both suppressed PAR-2 in colonic epithelial models [24,43], which argues that the effect is not a simple function of passive lipophilicity and raises the possibility of transporter-mediated uptake in colonocytes. Second, and more consequentially, much in vitro statin pharmacology, including our own, employs micromolar concentrations that exceed by orders of magnitude the free plasma concentrations achieved at standard clinical doses (Section 12.4), so the selectivity claim requires demonstration at clinically achievable exposures before it can support a repurposing argument. The definitive experimental set is therefore: isogenic cells expressing PAR-1 or PAR-2 alone; parallel concentration–response curves for trypsin at PAR-2 and thrombin at PAR-1; geranylgeranyl pyrophosphate, farnesyl pyrophosphate and mevalonate add-back; cholesterol depletion with methyl-β-cyclodextrin and repletion; palmitoylation blockade with 2-bromopalmitate; detergent-resistant membrane fractionation; and *F2RL1* and *F2R* transcript quantification, all performed across a concentration range that brackets clinically achievable exposure. On the present evidence, the transcriptional selectivity is directly demonstrated in our models [24,43], whereas the prenylation, raft and sorting explanations remain supported by indirect evidence.

### 10.4. Direct Oral FXa Inhibitors as Antitumour Agents

Direct oral anticoagulants targeting FXa offer an unusually well-developed pharmacological route to attenuate the coagulation arm of the PAR-2 axis (Figure 7). Edoxaban significantly suppressed tumour growth in colon26-inoculated BALB/c mice, with histological evidence of reduced inflammatory infiltrate [84], and a recent in vitro and in vivo study of colon cancer cells demonstrated that FXa drives ERK and AKT activation through PAR-2, with concomitant EGFR transactivation and IL-8 release; FXa inhibition attenuated each of these outputs [49]. The clinical population most likely to benefit from this approach is dual-indication patients with high tissue factor expression and elevated thromboembolic risk, particularly in PDAC and metastatic CRC, and biomarker-stratified prospective trials are warranted. The risk–benefit calculus must, of course, accommodate the bleeding risk inherent to anticoagulation, and the integration of activated PAR-2 fragment measurement into trial design would help define the population in whom the antitumour benefit outweighs the haemorrhagic risk.

### 10.5. Nutraceutical and Natural-Product Modulators

Nutraceutical agents represent a translationally pragmatic complement to direct receptor antagonism (Figure 7). We have shown that oleocanthal selectively modulates PAR-2-driven inflammatory pathways in CRC, attenuating TNF-α and calcium dysregulation [26,177], and that curcumin inhibits PAR-2-induced ERK phosphorylation, calcium mobilisation and anti-apoptotic signalling in inflammation-driven CRC, with computational evidence for direct curcumin–PAR-2 transmembrane-domain interaction [25]. Vitamin D and curcumin combinations have shown anti-inflammatory and anti-progressive effects in chondrocyte and gastrointestinal cell systems [178], supporting a broader anti-inflammatory paradigm in which nutraceuticals constrain pathological PAR-2 output without abolishing its physiological roles. EGCG, thymoquinone, boswellic acids and gingerol-/shogaol-derived bioactives are additional candidates whose mechanistic profiles intersect with PAR-2-coupled inflammatory and TGF-β signalling. Critical limitations remain in bioavailability and standardisation; addressing these constraints should be a near-term priority, particularly for translation in CRC chemoprevention and PDAC adjunctive therapy.

### 10.6. Combination Logic and Biomarker-Guided Deployment

The mechanistic and translational evidence reviewed above supports a series of combination strategies that should be tested in biomarker-defined patient populations. PAR-2 inhibition combined with EGFR-targeted antibodies addresses the Bcl-xL/RNF152-dependent resistance mechanism in CRC [22]; PAR-2 inhibition combined with MEK or ERK inhibitors targets adaptive MAPK reactivation in inflammatory tumours; FXa inhibition combined with cytotoxic chemotherapy attenuates the coagulation–PAR-2-driven survival programme, particularly in PDAC; statin-based chemoprevention combined with EGFR-targeted therapy addresses prenylation-dependent receptor trafficking and PAR-2 expression simultaneously; nutraceutical adjuncts (curcumin, oleocanthal) layered onto cytotoxic regimens may reduce inflammatory survival signalling and restore apoptotic competence; and PAR-2 modulation must be approached cautiously in immunotherapy-eligible patients, where biomarker selection (microsatellite status, CD8 infiltration, activated PAR-2 fragment level) should govern decision-making (Table 4).

## 11. Biomarker Strategy for the Protease–PAR-2 Axis

### 11.1. Why F2RL1 mRNA Is Necessary but Not Sufficient

A recurrent methodological pitfall in PAR-2 oncology is the conflation of *F2RL1* mRNA expression with PAR-2 activity. Because PAR-2 is activated by irreversible proteolytic cleavage of its N-terminus rather than by reversible ligand binding, transcript-level data cannot distinguish a quiescent receptor from one that has been cleaved, signalled and internalised. This is more than a semantic distinction: cleaved-and-internalised PAR-2 may continue to signal from endosomes through β-arrestin-coupled mechanisms [59,179], and surface receptor density can be transiently depleted in tumours where activation is highest. A biomarker strategy fit for clinical translation must therefore integrate transcriptional, protein-level, surface-density and activation-fragment readouts.

### 11.2. Activated PAR-2 Fragments as Circulating Biomarkers

Recent assay development has demonstrated that the N-terminal activation fragment of PAR-2 can be measured in serum. The PRO-PAR2 neoepitope ELISA captures fragments generated by serine-protease activity and has been used to demonstrate elevated systemic PAR-2 activation in rheumatoid arthritis, with a reduction in response to anti-IL-6 receptor therapy [59]. Extension of this assay to gastrointestinal cancer cohorts is straightforward in principle and would provide a non-invasive readout of systemic PAR-2 axis activation, with potential utility both for patient stratification and for pharmacodynamic monitoring of PAR-2-axis-modulating therapies.

The question of how viable the PRO-PAR2 serum assay is as a specific biomarker for gastrointestinal cancer deserves a candid appraisal. The assay is analytically validated and has demonstrated clinical responsiveness in inflammatory disease, where circulating activation fragments fall in response to anti-IL-6-receptor therapy, establishing proof of principle that a neoepitope generated by serine-protease cleavage of PAR-2 can be measured reproducibly in serum and tracked pharmacodynamically. Its principal limitation for oncology is specificity: because PAR-2 is cleaved by proteases active in many inflammatory, infective and thrombotic states, a raised PRO-PAR2 signal reports systemic PAR-2 activation rather than a tumour-specific event, and cancer-specific diagnostic thresholds have not yet been established. The realistic near-term role of PRO-PAR2 in CRC and PDAC is therefore not as a stand-alone diagnostic but as a pharmacodynamic and stratification biomarker deployed within a composite panel: combined with tumour *F2RL1* expression, tissue-factor and FXa activity, faecal protease activity in CRC, and immune-contexture markers, a longitudinal fall in PRO-PAR2 could serve as an accessible, non-invasive readout of target engagement during PAR-2-axis-directed or anticoagulant-repurposing therapy. Prospective evaluation in biomarker-defined CRC and PDAC cohorts, benchmarked against tissue activation-state readouts, is required before any tumour-specific claim can be sustained [59].

#### Stage Dependence, Benign Inflammatory Confounding and the Discriminant Problem

A question that follows directly from this appraisal, and one that must be answered before PRO-PAR2 can be advanced into a gastrointestinal oncology setting, is how the serum signal behaves across tumour stage and whether it can be distinguished from the signal generated by benign inflammatory disease. We state the position plainly; no stage-resolved PRO-PAR2 dataset has been published in CRC or PDAC, and no study has compared PRO-PAR2 in gastrointestinal cancer against an actively inflamed benign comparator group. The analysis that follows is therefore offered as a mechanistic framework and an assay-design specification rather than as a synthesis of established data, and it is labelled hypothesis-generating throughout.

The behaviour of the analyte follows from what it physically is. The PRO-PAR2 competitive ELISA quantifies the 36-residue N-terminal peptide liberated when a serine protease cleaves PAR-2 at the canonical R36↓S37 site, and it was validated against recombinant receptor cleavage and against matriptase-treated human synovial explants, with intra- and inter-assay coefficients of variation of 6.4% and 5.8% respectively [59]. The assay therefore reports the whole-body integral of canonical PAR-2-directed proteolysis. It is not a tumour assay, and it was never designed as one; its reference populations comprised 22 healthy individuals, 23 patients with osteoarthritis and 15 with rheumatoid arthritis, in whom the signal was elevated in rheumatoid arthritis and fell following tocilizumab treatment [59]. That result is the clearest possible demonstration that the analyte tracks systemic inflammatory protease burden and responds to its suppression. For an oncology application, this inverts the usual framing: benign inflammation is not a nuisance variable to be controlled for; it is a co-primary determinant of the measurand.

The first oncological data have now appeared and are instructive without being decisive. Serum-activated PAR-2 has been quantified in healthy donors and in patients across several solid tumour types, and plasma PRO-PAR2 above the cohort median was associated with inferior overall survival in patients with non-small-cell lung cancer receiving pembrolizumab with platinum-based chemotherapy [128]. Two conclusions follow. First, the analyte is measurable in cancer and carries outcome information in at least one immunotherapy-treated population, which establishes feasibility. Second, the cohorts were small, no gastrointestinal stage stratification was performed, and no benign inflammatory comparator was included, so the question posed here remains formally unanswered.

On mechanistic grounds, a clean monotonic stage gradient should not be expected from the current single-analyte format because the serum fragment pool is the sum of anatomically and biologically distinct proteolytic sources whose relative contributions change with stage. In early disease, spanning the adenoma to carcinoma transition and stage I to II, the dominant PAR-2-directed proteases are mucosal and pericellular: mast-cell tryptase, matriptase released from the constraint of its inhibitor HAI-1/*SPINT1*, and microbial proteases at a disrupted barrier. These act within a small tissue volume and their contribution to a systemic fragment pool is correspondingly small, so the tumour-derived increment is likely to be submerged beneath the constitutive inflammatory background. In locally advanced disease, rising tissue-factor expression, mast-cell density and neutrophil infiltration increase the flux. In advanced and metastatic disease, and particularly in tissue-factor-high PDAC, systemic coagulation activation through TF–FVIIa–FXa becomes the dominant contributor [180]. The predicted relationship is therefore shallow or absent across early stages and steeper in advanced disease, which is precisely the profile least useful for early detection and most vulnerable to confounding, since the advanced-stage increment is generated by the same coagulation biology that accompanies venous thromboembolism, sepsis and recent surgery.

The discriminant problem can now be stated exactly. A patient with active ulcerative colitis, chronic pancreatitis or seropositive rheumatoid arthritis may generate a PRO-PAR2 concentration equal to or greater than that of a patient with stage II colorectal cancer, not because the assay is failing but because it is succeeding; it is reporting protease activity, and protease activity is what both conditions share. Any receiver-operating-characteristic analysis benchmarked against healthy donors will therefore overstate discrimination, sometimes grossly, and should not be accepted as evidence of cancer specificity.

Five design strategies follow from this analysis, and we set them out because they are actionable rather than aspirational. First, comparator selection: evaluation must include actively inflamed benign arms, at minimum, active inflammatory bowel disease and chronic pancreatitis for CRC and PDAC respectively, rather than healthy donors alone. Second, normalisation: indexing PRO-PAR2 to a generic inflammatory denominator such as C-reactive protein, interleukin-6 or faecal calprotectin may subtract the non-neoplastic component, and a PRO-PAR2 to CRP ratio may carry more tumour-specific information than either analyte alone. Third, and in our view, the decisive innovation, cleavage-site-resolved neoepitope panels: because different proteases cleave PAR-2 at distinct positions, generating distinct fragments, with trypsin and tryptase acting at the canonical R36↓S37 site, cathepsin S at E56↓T57 and neutrophil elastase at S67↓V68 [13,34], a panel of cleavage-site-specific assays would fingerprint the dominant protease rather than report aggregate activity, converting a non-specific inflammation marker into a protease-provenance readout in which a coagulation-dominant fingerprint could be distinguished from a neutrophil-dominant one. Fourth, compartment anchoring: pairing serum measurement with cleaved-PAR-2 neoepitope immunohistochemistry in matched tissue, and with faecal protease activity in CRC, tests whether the circulating signal reflects the tumour at all. Fifth, longitudinal intra-patient design: using change from an individual baseline during PAR-2-axis-directed or anticoagulant therapy makes each patient their own control and eliminates the between-patient inflammatory offset entirely, which is why pharmacodynamic monitoring, rather than diagnosis or staging, remains the highest-value near-term application of this assay. The determinants of the signal, their expected behaviour across stages and in benign disease, and the corresponding design responses are summarised in Table 5.

### 11.3. Tissue Panels and Multi-Omic Integration

A composite tissue biomarker panel for PAR-2-directed gastrointestinal oncology should capture (i) receptor abundance and distribution (*F2RL1* in situ hybridisation; PAR-2 IHC), (ii) activation state (cleaved-PAR-2 neoepitope IHC; activation fragments in matched serum) (Table 6), (iii) the proteolytic input (tissue factor IHC; mast-cell tryptase density; neutrophil elastase; matriptase), (iv) the canonical downstream nodes (phospho-ERK, phospho-p65, phospho-AKT, nuclear β-catenin, Bcl-xL/phospho-Bcl-xL Ser-145), (v) the immune contexture (CD8, FoxP3, CD68/CD163, microsatellite status) and (vi) the stromal context (α-SMA, FAP, collagen architecture). Spatial transcriptomic and multiplexed proteomic platforms are particularly well-suited to capture these layered readouts in a single specimen, and the integration of such panels with standard pathological and molecular reporting is feasible in an academic-cancer-centre setting.

### 11.4. Survival Evidence Associated with the Proposed Protease Signatures

Any biomarker signature must ultimately be judged by whether it carries prognostic information independent of established clinicopathological variables, and we address this question directly rather than by implication. To our knowledge, no published study has reported a Kaplan–Meier or multivariable survival analysis of the composite protease–PAR-2 signature proposed in this review, in either CRC or PDAC. The signature as formulated here, comprising receptor abundance, receptor activation state, dominant proteolytic input, downstream node activity and immune contexture, has not been assembled, scored or tested against outcome in any cohort. This is a genuine evidentiary gap, and it is the single most important obstacle between the mechanistic framework developed in this review and its clinical deployment.

What does exist is survival evidence for individual components, and it is both fragmentary and, importantly, internally contradictory (Table 7). In colorectal cancer, a 115-patient series reported that a high density of tryptase-positive mast cells, related to PAR-2 expression, constituted a poor prognostic factor [5]. In hepatitis-B-related hepatocellular carcinoma, a 202-patient resection series with tissue-microarray immunohistochemistry found that high PAR-2 expression was associated with reduced overall survival and disease-free survival and retained independent prognostic significance on multivariable analysis [176]. In non-small-cell lung cancer, plasma PRO-PAR2 above the median identified patients with inferior overall survival on pembrolizumab with platinum chemotherapy, and *F2RL1* expression has been reported as a negative biomarker of immune-checkpoint-inhibitor responsiveness [128]. Against these, a recent colorectal study found the opposite: high tumour-cell PAR-2 expression was associated with a favourable prognosis and correlated with CD8+ T-cell infiltration [181]. Tissue factor, the apex of the coagulation arm of the axis, correlates with histological grade, invasion and prothrombotic complications in pancreatic cancer [1,2,182].

These findings do not agree, and the disagreement is analytically informative rather than merely inconvenient. At least five factors reconcile them. First, the analytes are not equivalent: tissue receptor abundance and circulating activation fragment measure different quantities, and neither is a measure of receptor activation state in situ (Section 11.1). Second, the compartments differ: the favourable association was observed where PAR-2 was confined largely to tumour cells and tracked CD8+ infiltration [181], whereas the adverse association in CRC was driven by a stromal, mast-cell-dominated axis [5]; as Section 11.6.5 sets out, these compartments exert opposing effects on tumour immunity. Third, the immune contexts differ: in T-cell-inflamed tumours, the tumour-intrinsic chemokine arm of PAR-2 dominates and its loss is costly, whereas in inflammation-dominant, myeloid-rich tumours, the NF-κB arm dominates and its loss is beneficial. Fourth, the clinical settings differ: untreated resected cohorts and checkpoint-inhibitor-treated cohorts are not interchangeable, since PAR-2 modifies the very immune response that immunotherapy exploits. Fifth, none of these analyses adjusted for the others. The unavoidable implication is that any composite signature that fails to specify compartment, activation state and immune contexture will average opposing effects towards the null. The inconsistency of the univariate literature is therefore not an argument against a signature; it is the strongest available argument for one.

We therefore specify what an adequate study would require, since this aspect exposes a gap that the field must close rather than a deficiency that further narrative can repair. The design should be a retrospective–prospective biomarker study with an independent external validation cohort on resected stage I to III colorectal cancer and resected pancreatic ductal adenocarcinoma, powered on events rather than patients and sized to provide an order of fifteen events per candidate covariate. The score should be pre-specified and locked before any outcome analysis, with component weights estimated by penalised Cox regression, and it should be modelled as a continuous variable rather than dichotomised at a data-derived optimal cut-point, which is the single most common source of irreproducible biomarker claims. Overall survival should be the primary endpoint, with disease-free survival being secondary, analysed in a competing-risk framework where non-cancer mortality is material. Multivariable models must adjust at minimum for stage, grade, mismatch-repair or microsatellite status, consensus molecular subtype, *KRAS* and *BRAF* status, tumour sidedness, adjuvant therapy and age. Reporting should follow REMARK and should include the hazard ratio with confidence interval, the concordance index, calibration and time-dependent area under the curve, together with the incremental discrimination gained over a model containing stage alone, which is the only comparison that establishes clinical value. Until such a study is performed, the protease–PAR-2 signature should be described as biologically motivated but prognostically untested, and we describe it as such throughout this review.

### 11.5. PAR-2 in Precision Oncology: Stratification Biomarkers and Actionable Subgroups

**Figure 8:**
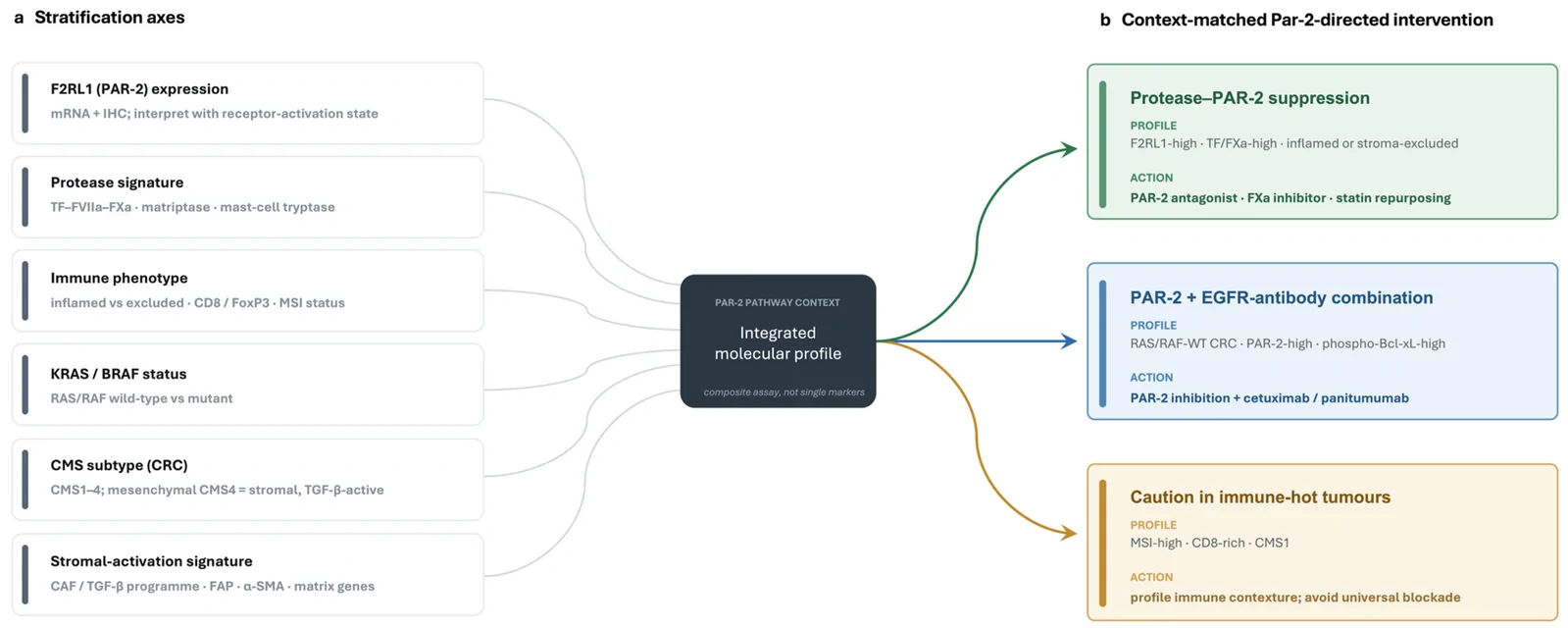
Biomarker-guided patient selection for PAR-2-directed therapy in gastrointestinal cancer. Conceptual framework linking molecular stratification to context-matched intervention across colorectal cancer (CRC) and pancreatic ductal adenocarcinoma (PDAC). (**a**) Six stratification axes serve as inputs to a composite molecular profile: *F2RL1* (PAR-2) expression, assessed by mRNA and immunohistochemistry and interpreted alongside receptor-activation state; the dominant protease signature (tissue factor–FVIIa–FXa, matriptase and mast-cell tryptase); immune phenotype (inflamed versus excluded contexture, CD8 and FoxP3 densities, and microsatellite-instability status); *KRAS*/*BRAF* mutational status; the consensus molecular subtype (CMS) in CRC, with the mesenchymal CMS4 subgroup denoting stromal, TGF-β-active, immunosuppressed biology; and a transcriptomic stromal-activation signature (cancer-associated-fibroblast and TGF-β programmes, FAP, α-SMA and matrix-remodelling gene sets). The axes are read in combination rather than individually, converging on an integrated PAR-2 pathway profile. (**b**) The integrated profile is mapped onto three context-matched decision nodes, colour-coded by therapeutic direction. Green (protease–PAR-2 suppression): *F2RL1*-high, tissue-factor/FXa-high, inflamed or stroma-excluded tumours, in which direct PAR-2 antagonism, FXa inhibition or statin repurposing is predicted to be most beneficial. Blue (PAR-2 plus EGFR-antibody combination): RAS/RAF-wild-type CRC with high PAR-2 and phospho-Bcl-xL, in which PAR-2 inhibition combined with cetuximab or panitumumab may forestall or reverse EGFR-targeted-therapy resistance. Amber (caution in immune-hot tumours): microsatellite-instability-high, CD8-rich, CMS1 tumours, in which tumour-intrinsic PAR-2 may sustain productive antitumour immunity and universal blockade should be avoided pending immune-contexture profiling. Arrows denote the flow from measurement to context-matched decision; the tumour-context-to-decision mapping is detailed in Table 8, Table 9 and Table 10. The framework is presented as a hypothesis-generating scaffold to discipline trial design rather than a validated clinical algorithm, and the stratification axes should be evaluated prospectively using composite assays rather than single markers. Abbreviations: CRC, colorectal cancer; PDAC, pancreatic ductal adenocarcinoma; PAR-2, protease-activated receptor-2; *F2RL1*, the gene encoding PAR-2; TF, tissue factor; FVIIa/FXa, activated coagulation factors VII and X; CMS, consensus molecular subtype; TGF-β, transforming growth factor-β; FAP, fibroblast activation protein; α-SMA, α-smooth muscle actin; EGFR, epidermal growth factor receptor; Bcl-xL, B-cell lymphoma-extra large. [Note: Created in Microsoft PowerPoint by the authors].

The context-dependence that is the central theme of this review carries a direct clinical corollary. If PAR-2 output is calibrated by the identity and source of the activating protease, by oncogenic background, and by stromal and immune context, then PAR-2-directed intervention cannot rationally be deployed as one-size-fits-all receptor blockade but must be matched to molecularly defined subgroups. This aligns the protease–PAR-2 axis with the prevailing logic of precision oncology, in which molecular stratification rather than histology increasingly governs treatment selection. Section 11 addressed how PAR-2 pathway activity can be measured; this section sets out how those measurements, integrated with tumour genomics and microenvironmental phenotyping, could be used to identify clinically actionable subgroups. Six stratification axes are particularly tractable (Figure 8).

(i) *F2RL1* expression

Tumour *F2RL1* (PAR-2) expression, assessed at transcript level and by immunohistochemistry and interpreted alongside activation-state readouts, identifies tumours that possess the receptor substrate for protease–PAR-2 signalling. In colorectal cancer, PAR-2 immunoreactivity increases from normal mucosa through adenoma to carcinoma, is highest at the invasive front, and associates with advanced stage, poor differentiation and unfavourable prognosis [5]. Critically, PAR-2 expression is not merely prognostic but potentially actionable: in patient-derived colorectal xenografts, PAR-2 stabilises Bcl-xL through Ser-145 phosphorylation and displacement of the E3 ligase RNF152, and PAR-2 silencing or inhibition restores sensitivity to EGFR-targeted antibodies [22]. *F2RL1*-high tumours therefore define the population in which the axis is most likely to be operative and druggable, provided expression is read together with markers of receptor activation rather than in isolation (Section 11.1).

(ii) Protease signatures (tissue factor–FVIIa–FXa, matriptase, tryptase)

Because PAR-2 is an irreversibly cleaved receptor whose output is dictated by its activating protease, the dominant proteolytic input is itself a stratifier that indicates which upstream-directed therapy is mechanistically appropriate. A tissue factor–FVIIa–FXa-dominant signature, characteristic of the procoagulant desmoplastic PDAC microenvironment, nominates direct oral FXa inhibitors and TF-pathway-directed agents [tissue factor–FVIIa–FXa–PAR-2 axis in PDAC (Section 5.4)]; a matriptase-dominant signature, reflecting loss of the HAI-1 (*SPINT1*) brake on epithelial matriptase, nominates matriptase/TMPRSS-directed inhibition and is supported by genetic evidence that *Spint1* loss accelerates *Apc*-driven intestinal tumourigenesis [50]; and a mast-cell-tryptase-rich signature, which co-localises with PAR-2 and adverse prognosis in colorectal cancer, nominates tryptase inhibition [5]. Practically, this axis can be captured by tissue-factor immunohistochemistry, chromogenic FXa activity, matriptase and tryptase staining, and, in colorectal cancer, faecal protease activity.

(iii) Immune phenotype

The immune contexture governs whether PAR-2 blockade is likely to relieve immune exclusion or, conversely, to compromise protective tumour-intrinsic PAR-2-supported immunity, and it is now a validated stratifier in colorectal cancer. The type, density and location of tumour-infiltrating lymphocytes predict outcome independently of stage [183], a principle formalised and internationally validated as the Immunoscore [184]. Mismatch-repair status is decisive: mismatch-repair-deficient/microsatellite-instability-high tumours are hypermutated, CD8-rich and highly responsive to PD-1 blockade [185], and in such immune-hot tumours, tumour-intrinsic PAR-2 may be sustaining rather than opposing antitumour immunity, so universal PAR-2 blockade should be approached with caution. Inflamed-versus-excluded scoring together with CD8, FoxP3 and CD68/CD163 densities and MSI testing therefore stratifies patients into those likely to benefit from PAR-2 suppression and those in whom it may be counterproductive.

(iv) *KRAS and BRAF mutational status*

*KRAS* and *BRAF* status determines both MAPK-pathway wiring and, in colorectal cancer, eligibility for EGFR-targeted therapy. Anti-EGFR antibody benefit is confined to RAS/RAF-wild-type disease [186], which is precisely the population in which the PAR-2–Bcl-xL–RNF152 sensitisation strategy is expected to add value, since combining PAR-2 inhibition with cetuximab or panitumumab could forestall or reverse acquired resistance [22]. In *KRAS*-mutant PDAC, by contrast, constitutive MAPK signalling reframes PAR-2 as an amplifier of pre-existing *KRAS*–ERK addiction rather than an independent driver (Section 5.5), positioning PAR-2 or upstream-protease modulation as a means of lowering oncogenic signalling tone rather than as a substitute for direct *KRAS*-directed therapy. Targeted next-generation sequencing for *KRAS*/*BRAF* is thus a prerequisite for rational deployment.

(v) Consensus molecular subtypes (CMS) of colorectal cancer

The consensus molecular subtype classification provides a ready-made framework onto which protease–PAR-2 biology can be mapped [187]. The mesenchymal subtype CMS4 is defined by stromal infiltration, TGF-β pathway activation, angiogenesis and an immunosuppressed, poor-prognosis phenotype—the same biology through which PAR-2 acts in the stroma—whereas CMS1 is microsatellite-unstable and immune-activated. The prognostic power of these classifiers derives substantially from stromal rather than epithelial gene expression [188], and immune/stromal CMS-linked classifications are relevant for precision immunotherapy [189,190]. CMS4 is therefore predicted to be the colorectal subgroup in which protease–PAR-2 and stromal-directed modulation is most influential, while CMS1/MSI tumours fall into the immune-caution category defined above.

(vi) Transcriptomic signatures of stromal activation

Independent of formal subtyping, transcriptomic signatures of stromal activation—myofibroblastic and inflammatory CAF programmes, FAP, α-SMA, TGF-β-response and matrix-remodelling gene sets—flag the desmoplastic, mechanically stiff tumours in which the protease–PAR-2–mechanotransduction loop developed in Section 7.2.1 and Section Three Mechanistically Distinct Arms of Stromal Resistance is most active. High stromal-gene expression defines poor-prognosis colorectal cancer driven by cancer-associated fibroblast and TGF-β programmes [191], and TGF-β-driven stromal signalling enforces immune exclusion and metastatic competence that can be reversed by TGF-β blockade in preclinical models [192]. A high stromal-activation signature therefore identifies tumours in which selective protease–PAR-2 modulation, rather than indiscriminate stromal ablation, which can be counterproductive (Section Three Mechanistically Distinct Arms of Stromal Resistance), is the mechanistically preferred strategy, potentially in combination with TGF-β-axis or FXa-directed agents.

**Table 8:** Candidate stratification biomarkers for PAR-2-directed therapy in colorectal and pancreatic cancer.

Stratification Axis	Readout/Assay	Biological Meaning	Therapeutic Implication
*F2RL1* expression	RNA-seq/qPCR; PAR-2 IHC; cell-surface flow cytometry	Receptor substrate available for protease activation; transcript necessary but not sufficient	High expression flags candidates for PAR-2-axis suppression; interpret with activation-state markers
Protease signature	Tissue factor IHC; chromogenic FXa; tryptase density; matriptase; faecal protease (CRC)	Identifies the dominant activating input to PAR-2	Directs choice of upstream-protease-targeted therapy (FXa inhibitor, tryptase/matriptase blockade)
Immune phenotype	CD8, FoxP3, CD68/CD163; inflamed-vs-excluded scoring; MSI status	Determines whether PAR-2 relieves or supports antitumour immunity	Inflamed/excluded → likely benefit from blockade; immune-hot/MSI-high → caution
*KRAS*/*BRAF* status	Targeted NGS	MAPK wiring; EGFR-therapy eligibility in CRC	RAS/RAF-WT CRC → PAR-2 + EGFR-antibody combination; *KRAS*-mutant PDAC → PAR-2 as MAPK-pressure modifier
CMS subtype (CRC)	Transcriptomic CMS classifier	CMS4 mesenchymal/stromal subgroup is protease-rich and TGF-β-active	CMS4 predicted most responsive to protease–PAR-2 and stromal-directed modulation
Stromal-activation signature	myCAF/iCAF, FAP, α-SMA, TGF-β-response gene sets; collagen architecture	Marks desmoplastic, mechanically stiff tumours (PAR-2–mechanotransduction loop active)	High stromal activation → selective PAR-2/protease modulation rather than stromal ablation

#### 11.5.1. Biological Heterogeneity and the Dual Role of PAR-2

Much of the literature, and necessarily much of this review, emphasises the pro-tumorigenic functions of PAR-2, and it is important to state explicitly that this emphasis reflects the weight of available evidence rather than a settled or uniform biology. A balanced reading requires that contradictory findings, tumour-stage-specific effects and cell-type-specific functions be given due prominence (Table 9). The clearest illustration is the PAR-2 immunity paradox: in the FXa–PAR-2 MC38 colon-cancer model, genetic PAR-2 deficiency reduces tumour growth, consistent with a tumour-promoting role, yet in other immunocompetent settings, tumour-intrinsic PAR-2 appears to sustain chemokine output and CD8 T-cell infiltration such that its loss may attenuate checkpoint-inhibitor efficacy. These outcomes are not necessarily contradictory once compartment and context are specified. PAR-2 signalling in the epithelial tumour cell, the cancer-associated fibroblast, the endothelial cell and the infiltrating immune cell is mechanistically distinct: epithelial PAR-2 favours survival, β-catenin amplification and EGFR-therapy resistance; stromal/CAF PAR-2 drives a fibroinflammatory, immune-excluding phenotype; endothelial PAR-2 supports angiogenesis and permeability; and immune-cell PAR-2 can be either immunosuppressive (myeloid) or supportive of effector recruitment depending on the cytokine milieu. Tumour stage adds a further axis, with early protease–PAR-2 inflammation promoting initiation while late-stage signalling shapes invasion, stromal architecture and immune exclusion. The implication for therapy is that the relevant question is not whether PAR-2 is pro- or anti-tumorigenic in the abstract, but which compartment dominates PAR-2 output in a given tumour and at a given stage. Table 9 summarises representative evidence on each side of this balance, organised by cellular compartment, to help readers calibrate the strength and direction of the available data.

**Table 9:** Compartment- and context-dependent effects of PAR-2 in gastrointestinal cancer: representative pro-tumour versus potentially protective evidence.

Compartment/Context	Pro-Tumour Evidence	Potentially Protective/Opposing Evidence	Evidence Strength
Tumour epithelial cell	Survival (Bcl-xL/RNF152), β-catenin/periostin, EGFR-therapy and 5-FU resistance in CRC; TGF-β/ALK5 motility in PDAC	Tumour-intrinsic PAR-2 may sustain chemokine output and CD8 infiltration in some immunocompetent models	Pro-tumour: directly demonstrated. Protective: supported by indirect evidence
Cancer-associated fibroblast	iCAF skewing, IL-6/CXCL1 secretion, ECM deposition and contractility supporting exclusion	Architectural stroma can restrain invasion; stromal ablation accelerates some PDAC models	Pro-tumour: supported by indirect evidence. Restraining role: directly demonstrated for ablation
Endothelial cell	TF–FVIIa-driven IL-8/CXCL1/VEGF, angiogenesis, permeability, metastatic niche	Context-limited; no consistent protective role reported	Pro-tumour: supported by indirect evidence
Immune cell (myeloid/T)	Myeloid PAR-2 → M2-like polarisation, immunosuppression; NF-κB cytokine milieu	Effector-supportive chemokine output; PAR-2 loss may impair anti-PD-1 efficacy	Both directions: hypothesis-generating/supported by indirect evidence
Tumour stage	Early protease–PAR-2 inflammation promotes initiation (*Apc*^Min/+^ genetic evidence)	Stage-specific reversal of net effect plausible but not formally mapped	Initiation: directly demonstrated. Stage reversal: hypothesis-generating

#### 11.5.2. A Decision Framework for Context-Matched PAR-2 Intervention

Used in combination rather than in isolation, these six axes define a tractable framework for biomarker-guided patient selection (summarised in Table 8). The framework implies a tiered decision logic (Figure 8). Tumours that are *F2RL1*-high, tissue-factor-high and activated-PAR-2-high, with an inflammation-dominant or stroma-excluded immune phenotype, constitute the population in whom protease–PAR-2 suppression (direct antagonism, FXa inhibition, or statin repurposing) is most likely to yield benefit. RAS/RAF-wild-type colorectal cancer with high PAR-2 and phospho-Bcl-xL is the natural population for PAR-2-plus EGFR antibody combination. Conversely, mismatch-repair-deficient, CD8-rich, immune-hot colorectal cancer warrants caution because tumour-intrinsic PAR-2 may therefore be sustaining productive antitumour immunity. This logic should be tested prospectively with composite assays rather than single markers and is best regarded, at present, as a hypothesis-generating but mechanistically grounded scaffold for trial design. The corresponding tumour-context-to-decision mapping is given in Table 10.

**Table 10:** Decision framework linking tumour context, expected PAR-2 role, biomarker readouts and therapeutic implication.

Tumour Context	Expected PAR-2 Role	Likely Biomarker Readout	Therapeutic Implication
Inflammation-dominant CRC (IBD-associated, mast-cell-rich)	Pro-tumour (NF-κB/cytokine amplification)	*F2RL1*-high, tryptase-high, faecal protease-high, raised PRO-PAR2	PAR-2-axis suppression; tryptase/matriptase or statin/nutraceutical modulation
RAS/RAF-WT metastatic CRC	Pro-tumour (Bcl-xL/RNF152 resistance)	PAR-2-high, phospho-Bcl-xL(Ser-145)-high	PAR-2 antagonist or statin + EGFR-targeted antibody combination
MSI-high/immune-hot CRC	Possibly protective (supports CD8 immunity)	MSI-high, CD8-rich, inflamed phenotype	Caution: avoid universal blockade; profile immune contexture before intervention
TF-high, desmoplastic PDAC	Pro-tumour (coagulation/stromal/TGF-β)	TF-high, FXa-active, stromal-activation signature high	FXa inhibitor repurposing; selective protease–PAR-2 modulation; chemo-sensitisation
Perineural-invasion-dominant PDAC	Pro-tumour + pro-nociceptive	PAR-2-high at nerves; clinical pain burden	PAR-2 antagonism as combined anti-invasive/anti-pain strategy (where agents are available)

### 11.6. Biological Heterogeneity and the Context-Dependent Duality of PAR-2

Much of this review, in common with much of the primary literature, emphasises the pro-tumorigenic functions of PAR-2. It is important to state explicitly that this emphasis reflects the current weight of evidence rather than a settled or uniform biology, and that a balanced reading must give due prominence to contradictory findings, to stage-specific effects, and to the distinct and sometimes opposing functions of PAR-2 in different cellular compartments.

#### 11.6.1. Contradictory Findings and the Immunity Paradox

The clearest contradiction is the PAR-2 immunity paradox. In the FXa–PAR-2 MC38 colon-cancer model, genetic PAR-2 deficiency reduces tumour growth in immunocompetent mice, consistent with a net tumour-promoting role [49]. Yet, in other immunocompetent settings, tumour-intrinsic PAR-2 appears to sustain inflammatory chemokine output, CD8^+^ T-cell infiltration and effector function, such that its loss may paradoxically attenuate anti-PD-1 efficacy [8,26]. These observations are not necessarily irreconcilable: their apparent conflict dissolves once the dominant compartment, the activating protease and the disease stage are specified. This is why the same molecular intervention (PAR-2 blockade) is predicted to be beneficial in one immune context and counterproductive in another, and why the central translational caveat in the field is that PAR-2 modulation must be biomarker-guided rather than universal.

#### 11.6.2. Cell-Type-Specific Functions and Compartmental Differences

PAR-2 signalling in the tumour epithelial cell, the cancer-associated fibroblast, the endothelial cell and the infiltrating immune cell is mechanistically distinct and cannot be treated as a single phenotype. In the epithelial compartment, PAR-2 drives survival through Bcl-xL stabilisation and interference with RNF152-dependent degradation, amplifies β-catenin and ERK/AKT/NF-κB output, and confers EGFR-targeted-therapy resistance [22]. In the stromal compartment, CAF PAR-2 intersects with TGF-β/ALK5/SMAD and RhoA/ROCK signalling to drive a fibroinflammatory, matrix-stiffening and immune-excluding phenotype [27,29,163]. In the endothelial compartment, TF–FVIIa/FXa–PAR-2 signalling induces IL-8, CXCL1 and VEGF, increasing permeability and supporting the angiogenic and metastatic niche [27]. In the immune compartment, the direction of effect is itself context-dependent: myeloid PAR-2 favours an immunosuppressive, M2-skewed phenotype, whereas tumour-intrinsic and some immune-cell PAR-2 activity may support effector recruitment. A further and often-overlooked layer of complexity arises at the neutrophil interface, where neutrophil elastase, cathepsin G and proteinase-3 cleave PAR-2 downstream of the canonical tethered-ligand site, disarming Gq/calcium signalling while, in the case of elastase, redirecting the receptor towards β-arrestin/MAPK-biased output [34]. Peptide-agonist (SLIGKV) and trypsin data therefore cannot be assumed to represent faithfully the signalling that predominates in protease-rich, neutrophil-infiltrated tumours.

#### 11.6.3. Stage-Specific Effects

The net effect of PAR-2 also depends on disease stage. Early in colorectal carcinogenesis, deregulated protease–PAR-2 activity promotes initiation and adenoma expansion, as demonstrated by acceleration of *Apc*^Min/+^ tumorigenesis on loss of the matriptase inhibitor HAI-1/*SPINT1*, a phenotype rescued by *F2rl1* co-deletion [50]. In barrier-disrupted, microbiome-rich mucosa, microbial proteases activate epithelial PAR-2 and amplify inflammation in a stage-dependent manner [146]. In established and advanced disease, PAR-2 output is dominated by invasion, stromal remodelling, coagulation-linked angiogenesis and immune exclusion. The relevant question, therefore, is not whether PAR-2 is pro- or anti-tumorigenic in the abstract, but which compartment and which protease dominate its output at a given stage.

#### 11.6.4. Synthesis

Taken together, the balance of evidence supports a net pro-tumorigenic role for PAR-2 in most established colorectal and pancreatic cancers, but this generalisation conceals genuine heterogeneity: potentially protective tumour-intrinsic immune functions in MSI-high, CD8-rich tumours; ambiguous, protease-edited signalling at the neutrophil interface; and stage-dependent reversals in inflammation- and microbiome-associated carcinogenesis. Table 11 catalogues representative pro-tumour, ambiguous and potentially protective evidence across these compartments and stages, so that readers can calibrate both the direction and the interpretive confidence of the available data and avoid oversimplification. Where the evidence for a given effect is extrapolated rather than directly demonstrated in colorectal or pancreatic cancer, this is indicated in the main text using the directly demonstrated/supported-by-indirect-evidence/hypothesis-generating convention adopted throughout the review.

#### 11.6.5. Molecular Determinants of the Pro-Tumour Versus Immune-Supportive Output of PAR-2

Section 11.6.1, Section 11.6.2, Section 11.6.3 and Section 11.6.4 establish that PAR-2 output is context-dependent and that the immunity paradox is real rather than apparent. A more demanding question, and the one that determines whether the axis is druggable, is what, at a molecular level, decides which way the switch is thrown. We propose that the direction of PAR-2 output in tumour immunity is set by four nested determinants: the cellular compartment in which the receptor is engaged; the identity of the activating protease and hence the cleavage site; the transducer branch that is preferentially recruited and the character of the secretome it generates; and the baseline immune contexture on which that secretome acts. Each is now supported by direct experimental evidence, and together, they convert a paradox into a tractable and testable model (Table 12).

The first and most powerful determinant is compartment. Recent evidence establishes that tumour-intrinsic PAR-2 in colorectal cancer is immune-supportive: PAR-2 expression is confined largely to tumour cells, correlates with CD8+ T-cell infiltration and associates with favourable prognosis, while tumour-intrinsic PAR-2 deficiency reduces CD8+ infiltration and effector function, promotes tumour growth and attenuates the efficacy of anti-PD-1 therapy [181]. The same work shows that PAR-2 is required for dendritic-cell activation and differentiation towards the conventional type-1 subset, and that dendritic-cell PAR-2 deficiency markedly impairs CD8+ priming and tumour control [181]. The myeloid compartment behaves in the opposite direction: PAR-2 activation drives an alternatively activated, immunosuppressive macrophage phenotype [135], PAR-2 activity in myeloid cells is associated with impaired antigen-presentation programmes, and both global *F2rl1* deletion and pharmacological inhibition with the insurmountable negative allosteric modulator I-117 reduce immunosuppressive myeloid populations, increase CD8+ activation in tumours and draining lymph nodes, shift the cytokine milieu towards a T-helper-1 profile and potentiate anti-PD-1 therapy [128]. A further layer of complexity is that PAR-2 deficiency has itself been reported to enhance myeloid-mediated immunosuppression and promote colitis-associated tumorigenesis [138]. The resolution is that a whole-body knockout or a systemically administered antagonist reports the algebraic sum of opposing compartmental effects, weighted by the relative mass and activity of each compartment in that particular model. This, rather than irreproducibility, is the most parsimonious explanation for the apparently contradictory genetic literature, and it carries a direct methodological consequence: only conditional, compartment-restricted genetics, using *F2rl1*-floxed animals crossed to Vil1-Cre, LysM-Cre, *Itgax*-Cre and Cdh5-Cre drivers, can settle the question, and until such experiments are performed, the field will continue to generate contradictory whole-body results indefinitely.

The second determinant is the activating protease and the cleavage site it selects, which dictates not only which transducer is engaged but how long the receptor survives to engage it. Trypsin and mast-cell tryptase cleave at the canonical R36↓S37 position, exposing the SLIGKV tethered ligand and engaging the full Gαq, Gα12/13 and β-arrestin repertoire; cathepsin S cleaves at E56↓T57; and neutrophil elastase cleaves at S67↓V68, immediately adjacent to the first transmembrane helix, activating the receptor through a mechanism that does not depend on a conventional tethered ligand, disabling Gαq-coupled calcium signalling while preserving β-arrestin-dependent MAPK output [13,34]. Cathepsin G and proteinase-3 disarm the receptor for canonical signalling. Critically, cleavage site also determines post-endocytic fate: synthetic activating peptide and trypsin induce PAR-2 ubiquitination, lysosomal trafficking and degradation, whereas neutrophil elastase does not [93]. The elastase-cleaved receptor therefore escapes the ubiquitin-dependent downregulation that normally terminates signalling, so its biased, calcium-independent, MAPK-directed output is not only qualitatively distinct but longer-lived. In a neutrophil-rich tumour, the receptor population is consequently locked into a persistent biased state that has no counterpart in any peptide-agonist experiment, a point developed quantitatively in Section 12.5. Receptor pharmacology is additionally pH-sensitive, with the potency of trypsin at PAR-2 differing several-fold between pH 6.5 and pH 7.4 [128], so the acidic tumour microenvironment is itself a determinant of activation efficiency.

The third determinant, and in our view, the mechanistic crux, is which transducer branch dominates and therefore what kind of secretome the receptor generates. Two branches diverge from the same receptor with opposite immunological consequences. The Gαq–phospholipase C–calcium–protein kinase C–NF-κB branch generates an inflammatory secretome of interleukin-6, interleukin-8, CXCL1, tumour necrosis factor-α, cyclo-oxygenase-2 and VEGF, which recruits and polarises myeloid cells, supports angiogenesis and enforces immune exclusion; this is the pro-tumour arm. The PI3K–AKT–mTOR branch, by contrast, drives production of CXCL9 and CXCL10, the CXCR3 ligands that recruit effector CD8+ T cells, and it is this branch that accounts for the immune-supportive phenotype of tumour-intrinsic PAR-2 [181]. The paradox is therefore not a contradiction between two biologies but a competition between two branches of one biology, and its direction is a quantitative question about branch dominance. Two consequences follow. First, an agent that biases the receptor away from Gαq–NF-κB while sparing PI3K–AKT–mTOR–CXCL9/CXCL10 output would be mechanistically superior to indiscriminate orthosteric blockade, which removes both arms and whose net effect must then depend on the baseline contexture. Second, and immediately testable, the ratio of CXCL9 and CXCL10 to interleukin-8 within a tumour is a candidate readout of branch dominance and therefore a candidate stratification biomarker of who should and should not receive PAR-2 blockade. β-arrestin-scaffolded endosomal ERK signalling [20] adds an orthogonal duration axis to this scheme rather than a third direction.

The fourth determinant is the baseline immune contexture upon which the secretome acts, which decides whether the chemokine arm has anything to recruit. In mismatch-repair-deficient, CD8-rich, T-cell-inflamed tumours, there is an effector pool available, the CXCL9/CXCL10 arm is productive, and removal of PAR-2 is correspondingly costly. In immune-desert, myeloid-rich, inflammation-dominant tumours, the NF-κB arm dominates, there is no substantial effector pool for chemokine output to mobilise, and PAR-2 suppression is predicted to be beneficial. This determinant converts the paradox from an obstacle into a stratification variable, and it is the biological justification for the decision framework given in Table 10.

Taken together, these determinants specify a model in which the direction of PAR-2 output is the product of compartment, cleavage-site-determined transducer bias and branch dominance, conditioned by baseline immunogenicity. The model is falsifiable and we state the experiments that would falsify it. Compartment-restricted *F2rl1* deletion should reverse the direction of effect between epithelial and myeloid drivers in the same tumour model. Activating PAR-2 in vivo with a tryptase-rich versus an elastase-rich proteolytic input should produce divergent chemokine and cytokine profiles despite identical receptor occupancy. Branch-selective pharmacology, using a Gαq inhibitor such as YM-254890 against a PI3K inhibitor, should move the CXCL9/CXCL10 to interleukin-8 ratio in opposite directions and should correspondingly reverse the effect of PAR-2 engagement on CD8+ recruitment. If compartment-restricted deletion fails to reverse the direction of effect, or if the chemokine to cytokine ratio proves insensitive to branch-selective inhibition, the model is wrong. The compartmental and chemokine components of this scheme are directly demonstrated [128,181]; the branch-competition model and the integrated switch are hypothesis-generating.

## 12. Experimental Models and Methodological Considerations

### 12.1. CRC Models

Robust dissection of PAR-2 biology in CRC requires a coordinated panel of cellular, organoid and animal models. Among CRC cell lines, HT-29, Caco-2 and HCT-116 capture the principal phenotypic spectrum and respond reproducibly to PAR-2 activation; SW480/SW620 and DLD-1 add *KRAS*- and *APC*-mutant context, while T84 has been useful for studies of PAR-2-driven wound-healing-like behaviour [194]. Patient-derived organoids provide an essential bridge between cell-line pharmacology and human disease biology and allow direct testing of nutraceutical and statin combinations in genetically defined backgrounds [194]. In vivo, the *Apc*^Min/+^ mouse and *Spint1*-deficient extensions of this model are particularly informative for PAR-2-driven inflammatory tumourigenesis [50,51], and orthotopic and liver-metastasis models extend the analysis to colonisation and dissemination phenotypes.

### 12.2. PDAC Models

PDAC modelling for PAR-2 studies should encompass PANC-1, AsPC-1, CAPAN-1, MiaPaCa-2 and BxPC-3 cell lines, the KPC genetically engineered mouse model, patient-derived organoids and organotypic CAF co-cultures [19,27,29,48,86,99,164]. Orthotopic implantation into immunocompetent backgrounds is essential to capture the desmoplastic and immune-exclusionary features that distinguish PDAC, while three-dimensional CAF-tumour co-cultures enable mechanistic dissection of the stromal arm of the PAR-2 axis under controlled conditions [19,27,29,48,86,99,164].

### 12.3. Recommended Assays for Rigorous PAR-2 Phenotyping

A rigorous PAR-2 study minimally requires (i) *F2RL1* qPCR and total/surface receptor quantification, (ii) cleaved-PAR-2 neoepitope detection, (iii) calcium-flux imaging in real time, (iv) ERK/NF-κB/AKT/p38/JNK phosphorylation by Western blot or phospho-array, (v) β-arrestin recruitment and receptor internalisation assays, (vi) cytokine release by ELISA or multiplex, (vii) EMT marker quantification, (viii) migration and invasion assays in 2D and 3D systems, (ix) protease activity profiling (FXa/FVIIa/tryptase as appropriate) and (x) co-culture systems with CAFs, macrophages, mast cells, neutrophils and endothelial cells. PAR-1/PAR-2 selectivity should be demonstrated explicitly using receptor-specific antagonists and agonists.

### 12.4. Common Methodological Pitfalls

Several methodological pitfalls recur in the PAR-2 literature and deserve explicit attention. Synthetic activating peptides (SLIGRL-NH2, 2-furoyl-LIGRLO-NH2) do not necessarily replicate the biased pharmacology of physiological proteases; *F2RL1* mRNA does not equate to receptor activation; tumour cell lines lack the immune and stromal complexity that determines PAR-2 output in vivo; PAR-2 effects observed in immunodeficient hosts may not generalise to immunocompetent settings; protease identity (TF–FVIIa/FXa vs. tryptase vs. microbial proteases) materially shapes downstream signalling; species-specific differences in PAR-2 pharmacology require cautious extrapolation between mouse and human; and high-concentration in vitro pharmacology can produce artefactual responses that disappear at clinically relevant exposures. Reviewer-facing methodological transparency on each of these points materially improves the credibility of any PAR-2 study.

### 12.5. Activator Provenance in the Reviewed Literature: An Audit of Synthetic Peptides Versus Native Proteases

The methodological caveat noted above invites a quantitative question: what proportion of the evidence base underpinning this review rests on synthetic activating peptides rather than on native proteases? Because this determines how much of the field’s mechanistic knowledge is capable of reporting biased signalling at all, we audited the primary literature cited here. The scope was defined as primary experimental studies in which PAR-2 was activated in a cellular or in vivo system; narrative and systematic reviews, structural and biophysical studies, and purely clinical, epidemiological or expression-only studies without an activation step were excluded. Studies in scope were classified by the activator reported in their methods into four categories: synthetic activating peptide alone (SLIGRL-NH2, SLIGKV-NH2, 2-furoyl-LIGRLO-NH2 and congeners); native or recombinant protease alone (trypsin, tryptase, matriptase, FVIIa, FXa, neutrophil elastase, cathepsin G, microbial proteases); both; and genetic or pharmacological manipulation without exogenous activation. Of approximately fifty-five studies in the scope, approximately 30% relied exclusively on synthetic peptide agonists, approximately 40% used native or recombinant proteases alone, approximately 20% used both, and approximately 10% employed genetic or pharmacological manipulation without exogenous activation. Approximately half of the corpus therefore contains peptide-derived evidence, and close to one third of the mechanistic claims available in this field rest on peptide agonism alone. A minority of studies could not be classified unambiguously from the reported methods and were assigned to the category of the activator used for the principal experiment (Table 13).

How this alters signalling bias is not a matter of degree but of category, and six distinct consequences follow. First, peptides bypass the cleavage step entirely. They engage the orthosteric pocket directly and therefore carry no information about the rate-limiting biological event, which is proteolysis; the identity of the protease, its local concentration, the tone of its endogenous inhibitors such as HAI-1 and the serpins, and the spatial confinement of its activity are all lost. In vivo, PAR-2 is a protease sensor, whereas under peptide agonism, it becomes a conventional ligand-activated receptor, which is a different object. Second, and most consequentially, peptides cannot report cleavage-site selection. The tethered ligand that is generated depends on where the receptor is cut: trypsin and tryptase act at R36↓S37 to expose SLIGKV, cathepsin S acts at E56↓T57, and neutrophil elastase acts at S67↓V68 adjacent to the first transmembrane helix, activating the receptor by a mechanism that does not require a conventional tethered ligand [13]. A canonical peptide can only ever mimic the canonical product. The entire class of disarmed and non-canonically biased signalling, which is precisely what predominates in the neutrophil-rich and protease-rich tumours this review is concerned with, is therefore categorically invisible to peptide-based experiments rather than merely under-represented in them.

Third, activator identity determines the receptor’s post-activation lifecycle and therefore the duration of signalling. Synthetic activating peptide and trypsin induce PAR-2 ubiquitination, lysosomal trafficking and degradation, whereas neutrophil elastase does not [93]. Peptide-based experiments consequently model the self-limiting arm of PAR-2 biology and systematically under-report the persistent arm, which is the arm most likely to be operative in chronically inflamed tissue. Fourth, concentration and kinetics differ by orders of magnitude. Peptides are typically applied at tens to hundreds of micromolar, whereas proteases act at nanomolar concentrations; peptide agonism is synchronous, supramaximal and spatially uniform, whereas physiological proteolysis is pericellular, graded and sustained. The predictable consequence is that peptide studies over-represent Gαq-coupled calcium transients, which are concentration-driven and rapidly desensitising, and under-represent sustained β-arrestin-dependent MAPK output; at high concentrations, peptide selectivity across the PAR family also degrades. Fifth, peptides cannot reconstitute receptor-complex context. The TF–FVIIa–FXa ternary complex assembles on the membrane with defined stoichiometry and colocalisation with tissue factor [16,17,100], and matriptase acts in a membrane-anchored, HAI-1-regulated fashion; neither arrangement can be reproduced by a soluble peptide, and the coagulation biology that dominates PDAC is therefore inaccessible to peptide pharmacology in principle. Sixth, agonist potency at PAR-2 is pH-sensitive [94], and no systematic comparison of peptide and protease agonism has been performed at the acidic pH characteristic of the tumour microenvironment.

The practical consequence for reading this review is that its mechanistic claims are not uniformly supported, and we have signposted this through the evidence-strength convention used throughout. Claims concerning canonical Gαq–calcium–NF-κB output rest on a broad base that includes both peptide and protease data and are correspondingly secure. Claims concerning biased, non-canonical and disarmed signalling rest almost exclusively on the small number of studies that used native proteases [93]. Claims concerning coagulation-driven PAR-2 output rest on protease-based studies. Readers should weight these classes of claim differently. We therefore propose a reporting standard for the field: the activator, its source, its concentration and its measured activity should be stated explicitly; where a synthetic peptide is used, at least one native protease relevant to the disease context should be included as a comparator; catalytic dependence should be confirmed with protease inhibitor controls; cleavage-site provenance should be reported where cleavage-state-specific reagents permit; concentrations should be titrated into a physiologically plausible range; and a bias conclusion derived from peptide agonism should never be generalised to a protease-rich tissue without protease-based confirmation. Adoption of this standard would materially improve the interpretability of the PAR-2 literature, and the audit reported here indicates why it is needed.

## 13. Future Directions

The most consequential research priorities now confronting the PAR-2 oncology field can be organised under five headings. First, the field needs biomarker-stratified clinical trials of statins, FXa inhibitors and, when available, direct PAR-2 modulators in CRC and PDAC, designed around composite biomarker panels that combine *F2RL1* expression, activated PAR-2 serum fragments, tissue factor activity and immune contexture. Second, mechanistic dissection of biased PAR-2 pharmacology, including the differential consequences of orthosteric blockade versus negative allosteric modulation versus β-arrestin-biased ligands, is essential to rationalise drug design and to avoid abolishing cytoprotective signalling. Third, the microbiome-barrier-PAR-2 axis represents a largely untapped therapeutic landscape in CRC, and the integration of microbial protease profiling, faecal protease activity assays and probiotic/postbiotic interventions deserves systematic exploration. Fourth, the PAR-2 immunity paradox demands resolution; tumour-intrinsic versus stromal versus immune-cell PAR-2 contributions must be dissected with conditional and lineage-specific genetic models, and the patient subgroups in whom PAR-2 inhibition risks compromising antitumour immunity must be defined. Fifth, the nutraceutical arm of the PAR-2 axis, including curcumin, oleocanthal, EGCG, and vitamin D, deserves rigorous evaluation in well-powered chemopreventive and adjunctive clinical studies, with bioavailability and standardisation problems addressed up-front. Across all five priorities, the unifying methodological principle is that PAR-2-directed strategies must be biomarker-guided rather than empirical, with deployment matched to the proteolytic and immunological context of the individual tumour.

## 14. Conclusions

Protease-activated receptor-2 is best understood not as a single inflammatory GPCR but as a proteolytic decision node, a context-dependent integrator that translates the identity, source and intensity of extracellular proteolysis into specific intracellular oncogenic, inflammatory, fibrogenic and immunological programmes. In colorectal cancer, this integrative function links epithelial barrier dysfunction, microbial proteases, mast-cell tryptase, coagulation FXa, and cytokine-driven inflammation to NF-κB, ERK, Wnt/β-catenin and Bcl-xL/RNF152-dependent therapy resistance, with a paradoxical and biomarker-relevant contribution to antitumour immunity in selected immune-hot contexts. In pancreatic ductal adenocarcinoma, the same conceptual framework links tissue factor and pancreatic protease activation to TGF-β/ALK5-dependent motility, ATP-dependent migration, fibroinflammatory CAF skewing, perineural invasion and chemoresistance. The most translationally mature path forward is therefore not universal PAR-2 blockade but biomarker-guided modulation of the protease–PAR-2 axis through a tiered set of strategies: direct PAR-2 antagonists and biased negative allosteric modulators where available; FXa inhibitors in coagulation-rich tumours, particularly PDAC and metastatic CRC; statin chemoprevention and adjunctive use in inflammation-driven CRC; nutraceutical agents (curcumin, oleocanthal, EGCG, vitamin D) as bioavailable, low-toxicity modulators of inflammatory PAR-2 output; and rational combinations with EGFR-targeted, MAPK-pathway and biomarker-stratified immunotherapeutic regimens. Realising this programme will require the parallel development of validated biomarker pipelines that capture not only *F2RL1* expression but also activation fragments, downstream phosphorylation events, proteolytic input and immune contexture. Pursued with mechanistic discipline and translational humility, the PAR-2 axis is poised to deliver a genuinely new class of biomarker-guided interventions in two of the most challenging cancers of the gastrointestinal tract.

## 15. Limitations

This review has several limitations that should be acknowledged.

Firstly, the article was designed as a focused mechanistic narrative review rather than a systematic review or meta-analysis. Although the literature was searched using PubMed and Google Scholar and only English-language articles were included, the heterogeneity of available studies precluded formal quantitative synthesis. The studies discussed differ substantially in tumour type, experimental model, PAR-2 activator, receptor readout, signalling endpoint, pharmacological inhibitor, and functional assay. Consequently, the conclusions should be interpreted as a structured mechanistic synthesis rather than as a pooled estimate of effect size. Secondly, the evidence base for PAR-2 in gastrointestinal cancer is uneven across disease contexts. Colorectal cancer and pancreatic ductal adenocarcinoma provide strong conceptual models for PAR-2 biology, but the depth of direct experimental evidence differs between individual pathways. Some mechanisms, such as PAR-2-linked inflammatory signalling, calcium mobilisation, ERK/MAPK activation, NF-κB signalling, EGFR-resistance biology, and tissue factor–coagulation signalling, are supported by multiple experimental studies. In contrast, other themes, including PAR-2-dependent immune remodelling, microbiome-derived protease activation, stromal compartment-specific effects, perineural invasion, and chemoresistance in PDAC, remain less comprehensively validated in disease-specific systems. In these areas, the review necessarily integrates evidence from adjacent inflammatory, coagulation, epithelial, and non-gastrointestinal cancer models. Thirdly, many studies in the PAR-2 field rely on synthetic activating peptides such as SLIGKV-NH_2_, SLIGRL-NH_2_, or 2-furoyl-LIGRLO-NH_2_ to model receptor activation. While these ligands are useful pharmacological tools, they do not fully reproduce physiological protease-mediated receptor cleavage. Native proteases differ in cleavage site, spatial localisation, duration of receptor engagement, biased signalling output, receptor internalisation, and ability to generate canonical versus non-canonical PAR-2 activation states. Therefore, findings obtained using synthetic peptide agonists should not be assumed to represent the full biological complexity of trypsin-, tryptase-, matriptase-, kallikrein-, tissue factor–FVIIa, factor Xa-, neutrophil protease-, or microbiome-derived PAR-2 activation in the tumour microenvironment. Fourthly, a recurring limitation in the available literature is the incomplete distinction between PAR-2 expression and PAR-2 activation. Many studies report *F2RL1* mRNA expression, total PAR-2 immunoreactivity, or bulk receptor protein abundance, but these measures do not necessarily indicate functional receptor activation. PAR-2 is activated through irreversible N-terminal cleavage, and its biological output depends on the proportion of receptor that is cell-surface accessible, cleaved, internalised, recycled, or engaged in β-arrestin-dependent endosomal signalling. Future studies should therefore combine transcriptomic analysis, total and cell-surface receptor quantification, cleavage-state-specific assays, downstream signalling readouts, and where possible, neo-epitope or activated-fragment biomarkers such as PRO-PAR2. Fifthly, the translational interpretation of PAR-2 biology is complicated by its context-dependent role in tumour immunity. In many inflammatory and stromal settings, PAR-2 appears to promote tumour progression by amplifying NF-κB signalling, cytokine production, angiogenesis, invasion, and therapy resistance. However, some immunocompetent colorectal cancer models suggest that tumour-intrinsic PAR-2 may also sustain chemokine production, CD8^+^ T-cell infiltration, or responsiveness to immune checkpoint blockade. This apparent PAR-2 immunity paradox means that universal PAR-2 inhibition may not be uniformly beneficial across all gastrointestinal tumours. Biomarker-guided patient selection will likely be required to distinguish tumours in which PAR-2 primarily drives pathological inflammation and immune exclusion from tumours in which PAR-2 contributes to immune visibility. Sixthly, the therapeutic sections of the review are necessarily hypothesis-generating. Direct PAR-2 antagonists, biased allosteric modulators, pepducins, upstream protease inhibitors, factor Xa inhibitors, statins, curcumin, oleocanthal, and other nutraceutical or repurposed agents are mechanistically attractive, but few have been tested specifically in biomarker-selected CRC or PDAC models. In particular, the extrapolation of anticoagulant effects from factor Xa inhibition to antitumour PAR-2 modulation requires caution, because coagulation blockade, tumour-cell signalling, stromal remodelling, angiogenesis, and bleeding-risk biology may not align in a simple linear manner. Similarly, statins and nutraceutical agents may influence multiple pathways beyond PAR-2, and their apparent PAR-2-modulatory effects require further validation using receptor-specific genetic and pharmacological controls. Seventhly, pancreatic ductal adenocarcinoma presents a special limitation because of its unusually complex tumour microenvironment. PDAC contains tumour epithelial cells, cancer-associated fibroblasts, endothelial cells, immune cells, nerves, coagulation proteins, pancreatic proteases, and dense extracellular matrix. Current experimental models do not fully recapitulate this multicellular, protease-rich, hypoxic, immune-excluded, and mechanically stiff environment. Conventional two-dimensional cell-culture systems are useful for pathway dissection but may underestimate the contribution of stromal proteases, tissue factor-driven coagulation, CAF heterogeneity, matrix stiffness, perineural invasion, and immune suppression. More physiologically faithful platforms, including patient-derived organoids, organoid–CAF co-cultures, microfluidic systems, orthotopic xenografts, humanised models, and genetically engineered mouse models, will be required to define the therapeutic relevance of PAR-2 in PDAC more precisely. Eighthly, the review gives substantial attention to CRC and PDAC, but other gastrointestinal malignancies, including gastric cancer, oesophageal cancer, hepatobiliary cancers, and cholangiocarcinoma, are not discussed in comparable depth. This narrower focus was intentional, because CRC and PDAC offer complementary models of microbiome/inflammation-driven and coagulation/stroma-driven PAR-2 biology. Nevertheless, this scope limits the generalisability of the conclusions across the full spectrum of gastrointestinal cancers. Ninthly, the available literature remains limited by the lack of standardised biomarkers for PAR-2 pathway activity. At present, studies variably measure *F2RL1* mRNA, total PAR-2 protein, receptor cleavage, calcium flux, ERK phosphorylation, cytokine release, tissue factor expression, factor Xa activity, inflammatory protease abundance, or downstream pathway activation. Without harmonised biomarker panels, it remains difficult to compare studies or identify which patients are most likely to benefit from PAR-2-directed or protease-directed interventions. Future translational work should integrate PAR-2 activation markers with protease-source profiling, tumour immune contexture, stromal phenotype, coagulation status, and therapeutic-response data. Finally, because this review synthesises the rapidly evolving mechanistic and translational literature, some conclusions may require revision as newer structural, pharmacological, immunological, and clinical data emerge. In particular, the development of more selective PAR-2 antagonists, biased modulators, activated-receptor assays, spatial proteomics, and single-cell analyses may substantially refine the current model of PAR-2 as a proteolytic rheostat in gastrointestinal cancer.

## 16. Literature Search Methodology

Consistent with its design as a focused mechanistic narrative review rather than a systematic review or meta-analysis, the evidence base was assembled through a structured but non-systematic search of PubMed and Google Scholar. The structured search was conducted between January 2024 and May 2026 to ensure currency of the recent literature and was supplemented, irrespective of publication date, by foundational and landmark references identified through hand-searching of pivotal reference lists and prior domain knowledge; only English-language publications were considered. Search terms combined receptor nomenclature (protease-activated receptor-2, PAR-2, PAR2, *F2RL1*) with disease descriptors (colorectal cancer, colon cancer, pancreatic cancer, pancreatic ductal adenocarcinoma, PDAC) and mechanistic or therapeutic qualifiers (tissue factor, FVIIa, FXa, trypsin, tryptase, matriptase, kallikrein, ERK, NF-κB, PI3K–AKT, Wnt/β-catenin, TGF-β, cancer-associated fibroblasts, immune evasion, chemoresistance, statins, FXa inhibitors, nutraceuticals and activated PAR-2 biomarkers). Original mechanistic, preclinical, translational and biomarker studies, together with relevant reviews, were eligible for inclusion, whereas conference abstracts, editorials and non-peer-reviewed material were excluded unless they supplied essential context unavailable elsewhere. Titles and abstracts were screened for relevance, potentially eligible articles were retrieved and assessed in full text, duplicate records identified across the two databases were removed manually, and the reference lists of pivotal studies were hand-searched for additional sources. Because heterogeneity in tumour type, model system, activating protease, receptor readout and functional endpoint precluded formal quantitative synthesis, no meta-analytic pooling or formal risk-of-bias scoring was undertaken; instead, evidence was appraised and weighted qualitatively. Greater interpretive weight was given to findings that were genetically validated (for example, through receptor knockout or *F2RL1* silencing), reproduced across independent models or corroborated by orthogonal experimental approaches, and demonstrated directly in colorectal or pancreatic systems, and correspondingly less weight was given to single-model observations and to data extrapolated from adjacent inflammatory, coagulation or non-gastrointestinal cancer settings. Reflecting this appraisal, the strength of each mechanistic claim is signposted throughout the main text as directly demonstrated, supported by indirect evidence, or hypothesis-generating.

## Figures and Tables

**Table 1:** Protease-specific cleavage of PAR-2/*F2RL1* and biased signalling consequences. The downward arrow (↓) indicates the proteolytic cleavage site. Abbreviations: PAR-2, protease-activated receptor-2; *F2RL1*, *F2R* like trypsin receptor 1. Additional abbreviations used in the table: PLCβ, phospholipase C beta; IP3, inositol 1,4,5-trisphosphate; ERK, extracellular signal-regulated kinase; MAPK, mitogen-activated protein kinase; NF-κB, nuclear factor kappa B; TF, tissue factor; FVIIa, activated factor VII; FXa, factor Xa; PI3K, phosphoinositide 3-kinase; AKT, protein kinase B; CRC, colorectal cancer; PDAC, pancreatic ductal adenocarcinoma; IBD, inflammatory bowel disease; HAI-1, hepatocyte growth factor activator inhibitor type 1; KLK, kallikrein-related peptidase; GI, gastrointestinal.

	Principal PAR-2 Cleavage Site or Activation Mode	Tethered Ligand/Signalling Mode	Dominant Signalling Outputs	Biological Relevance in Cancer/Inflammation	GI Cancer Relevance	Clinical or Translational Implication
Trypsin/trypsin-like serine proteases	Canonical cleavage at Arg36↓Ser37 in human PAR-2	Exposes canonical human tethered ligand SLIGKV; rodent equivalent SLIGRL	Broad PAR-2 activation: Gαq/11–PLCβ–IP3–Ca^2+^, Gα12/13–RhoA, ERK/MAPK, NF-κB, β-arrestin recruitment	Prototype PAR-2 activation mechanism; relevant to epithelial inflammation, pancreatic protease biology, tumour-cell survival, migration and cytokine production	Highly relevant to PDAC, where pancreatic trypsin-like protease biology intersects with ductal tumour-cell PAR-2 activation; also relevant to CRC epithelial PAR-2 models used to define inflammatory and survival signalling	Useful positive control in cellular assays, but over-reliance on trypsin may oversimplify physiological protease diversity
Mast-cell tryptase	Generally considered canonical PAR-2 activation at or near Arg36↓Ser37	Canonical tethered-ligand-dependent activation	Ca^2+^ mobilisation, ERK/MAPK, NF-κB, inflammatory cytokine release, barrier dysfunction	Links mast-cell infiltration to epithelial PAR-2 activation, inflammation, pain, barrier disruption and tumour–stromal communication	Particularly relevant to CRC, IBD-associated carcinogenesis, inflamed mucosa, barrier disruption and tumour-associated mast-cell infiltration; also relevant to PDAC stromal inflammation	Particularly relevant in CRC/IBD-associated carcinogenesis and inflammatory tumour microenvironments; mast-cell/tryptase blockade may reduce PAR-2-driven inflammation
Kallikrein-related peptidases, especially KLK4, KLK5, KLK14	Trypsin-like canonical cleavage, typically converging on Arg36↓Ser37 or canonical tethered-ligand exposure	Canonical PAR-2 activation	Ca^2+^ mobilisation, NF-κB, ERK/MAPK, inflammatory transcription	Important in epithelial tissues and cancer-associated protease networks; may amplify tumour-cell inflammatory signalling	Potentially relevant to epithelial GI cancers as part of tumour-associated serine-protease networks, especially where epithelial protease dysregulation amplifies PAR-2-dependent invasion, inflammation and survival	Potential upstream protease targets; KLK expression may help stratify epithelial tumours with PAR-2-active protease states
Matriptase/MT-SP1	Canonical or canonical-like activation of PAR-2; can function downstream of tissue factor–dependent protease cascades	Tethered-ligand-dependent PAR-2 activation	ERK/MAPK, NF-κB, epithelial invasion, inflammatory cytokine output	Links epithelial membrane-anchored serine proteases to PAR-2 activation; may connect tissue factor signalling to epithelial invasion and tumour progression	Relevant to CRC and PDAC epithelial tumour biology, especially invasion, epithelial–stromal communication and PAR-2 activation within membrane-proximal protease complexes	Matriptase/HAI-1 axis is a candidate therapeutic and biomarker node in epithelial cancers, including GI malignancies
TF–FVIIa complex	PAR-2 activation at canonical/canonical-like extracellular N-terminal site; often facilitated by tissue factor on tumour cells	Tethered-ligand-dependent activation, often spatially organised at the tumour-cell surface	ERK/MAPK, PI3K–AKT, GSK-3β inhibition, β-catenin stabilisation, IL-8/VEGF induction	Couples tumour-cell tissue factor expression to oncogenic, angiogenic and inflammatory PAR-2 signalling	Especially relevant to PDAC, a tissue-factor-rich malignancy with coagulation activation, desmoplasia, angiogenesis and invasive behaviour; also potentially relevant to advanced CRC with coagulation/inflammatory activation	Particularly relevant to PDAC and TF-high malignancies; suggests a rationale for targeting TF–FVIIa–PAR-2 signalling rather than coagulation alone
Factor Xa/FXa	Direct PAR-2 activation and/or activation through TF–FVIIa–FXa ternary signalling complexes	Canonical/canonical-like PAR-2 activation; context-dependent signalling intensity	ERK/MAPK, AKT, NF-κB, cytokine production, angiogenic signalling	Bridges coagulation activation with tumour inflammation, stromal activation, angiogenesis and invasive behaviour	Highly relevant to PDAC, where coagulation activation and tumour-cell TF expression may sustain PAR-2 signalling; relevant to CRC where inflammation-associated coagulation may reinforce PAR-2-dependent cytokine and survival pathways	Direct oral FXa inhibitors such as apixaban, rivaroxaban and edoxaban are mechanistically relevant tools; therapeutic effect may extend beyond anticoagulation in selected PAR-2/TF/FXa-high contexts
Neutrophil elastase	Non-canonical cleavage downstream of the trypsin site; reported at Ser68↓Val69	Removes or bypasses canonical tethered-ligand signalling; acts as a biased PAR-2 agonist	Weak or absent canonical Ca^2+^ signalling; biased β-arrestin/MAPK-associated signalling; altered receptor trafficking	Converts neutrophil-rich inflammation into non-classical PAR-2 outputs; relevant to chronic inflammation, tumour-associated neutrophils and inflammatory remodelling	Relevant to CRC inflammatory niches, IBD-associated tumorigenesis, tumour-associated neutrophils and barrier injury; also relevant to PDAC neutrophil-rich, immunosuppressive stromal compartments	Important caveat: neutrophil protease-rich tumours may show PAR-2 signalling patterns not predicted by trypsin or SLIGKV peptide assays
Cathepsin G	Non-canonical cleavage downstream of canonical site; reported around Phe65↓Ser66	Often described as disarming PAR-2 by removing the canonical tethered-ligand region; biased signalling remains less certain	Loss of canonical Ca^2+^ signalling; may silence or remodel PAR-2 responsiveness rather than fully activate canonical pathways	Neutrophil-associated proteolysis may desensitise or reprogram PAR-2 rather than simply activate it	Relevant to neutrophil-enriched CRC and PDAC microenvironments, where PAR-2 signalling may be edited, desensitised or redirected rather than simply activated	Supports the idea that neutrophil-rich inflammation can create PAR-2 “signal editing”; therapeutic blockade must consider whether the protease is activating or disarming PAR-2
Proteinase 3	Non-canonical cleavage downstream of canonical site; reported around Val62↓Asp63	Generally considered non-canonical/disarming for PAR-2	Limited canonical Ca^2+^ mobilisation; potential alteration of receptor responsiveness	May modulate PAR-2 in neutrophil-rich inflammatory niches; functional relevance in cancer remains incompletely defined	Potentially relevant to GI tumours with strong neutrophil infiltration, particularly inflammation-associated CRC and aggressive PDAC, although direct GI-cancer evidence should be framed cautiously	Should be discussed cautiously; may be more relevant as a regulator of PAR-2 availability than as a classical agonist
Cathepsin S	Non-canonical cleavage reported in PAR-2 biased signalling literature	Generates non-classical PAR-2 activation profile	Biased signalling, including pain/inflammation-associated pathways; less canonical Gαq-dominant output	Relevant to inflammatory and immune-cell-rich microenvironments; may contribute to PAR-2-mediated pain, inflammation and immune remodelling	Potentially relevant to PDAC pain, perineural inflammation and immune remodelling, and to CRC inflammatory microenvironments; GI-cancer-specific mechanistic evidence remains less mature	Possible target in inflammatory disease and cancer-associated inflammation, but GI-cancer-specific evidence should be presented cautiously
Synthetic activating peptides: SLIGKV-NH_2_, SLIGRL-NH_2_, 2-furoyl-LIGRLO-NH_2_	Do not cleave PAR-2; directly mimic the exposed tethered ligand	Pharmacological orthosteric activation of PAR-2	Often strong canonical Gαq/Ca^2+^ and ERK responses, depending on peptide and cell type	Useful experimental tools, but they do not reproduce protease-specific cleavage, receptor truncation, spatial restriction or biased signalling	Commonly used in CRC and pancreatic cancer cell models to probe PAR-2 responsiveness, but they may not reproduce the protease-specific biology of microbiome-, mast-cell-, neutrophil-, TF–FVIIa- or FXa-driven activation	Major methodological caveat: peptide agonist data should not be equated with physiological protease activation in tumour microenvironments

**Table 2:** Step-resolved model of the PAR-2 endocytic itinerary, predicted effects of oncogenic *KRAS*, and the experimental readouts required to test them. The downward arrow (↓) indicates the proteolytic cleavage site.

Step	Molecular Machinery	Predicted Effect of Oncogenic *KRAS*	Net Effect on PAR-2 Output	Experimental Readout	Evidence Strength
Cleavage and activation	Serine protease acting at R36↓S37; TF–FVIIa–FXa in PDAC	Upregulated tissue factor sustains FVIIa and FXa generation and recleaves newly delivered receptor	Increased and prolonged	Cleavage-state-specific antibody; chromogenic FXa activity	Supported by indirect evidence.
Desensitisation	GRK phosphorylation; β-arrestin-1/2 uncoupling	No direct effect predicted	Neutral	NanoBiT or BRET β-arrestin recruitment kinetics	Mechanism directly demonstrated.
Internalisation	β-arrestin, clathrin, AP-2, dynamin	Unchanged, or modestly accelerated by macropinocytic membrane flux	Neutral to reduced	Reversible surface biotinylation pulse-chase; surface HiBiT	Mechanism directly demonstrated.
Endosomal signalling	β-arrestin-scaffolded Raf-1–MEK–ERK complex	Constitutive RAF–MEK flux sustains endosomal ERK, decoupling internalisation from termination	Increased	Compartment-restricted ERK activity reporter	Supported by indirect evidence.
Ubiquitination	c-Cbl, recruited Src-dependently	Reduced, through competition for a limiting c-Cbl pool by hyperactive receptor tyrosine kinases	Increased, through escape from degradation	Receptor ubiquitination immunoprecipitation; c-Cbl co-immunoprecipitation; Src inhibition control	Hypothesis-generating.
Alternative ligase input	RNF43 (Wnt target); RNF152	Reduced turnover where RNF43 is inactivated	Increased	RNF43 mutational status; receptor turnover assay	Supported by indirect evidence
ESCRT-dependent sorting	HRS–STAM (ESCRT-0); ESCRT-I, -II, -III	Unchanged unless upstream ubiquitination becomes limiting	Increased if ubiquitination falls	Rab5 and Rab7 colocalisation; HRS knockdown as positive control	Mechanism directly demonstrated.
Deubiquitination	AMSH and UBPY (USP8)	Not established	Unknown	AMSH (D348A) and UBPY (C786S) catalytically inactive mutants	Mechanism directly demonstrated.
Lysosomal degradation	Multivesicular body to lysosome; MiT/TFE-driven lysosomal biogenesis	Increased lysosomal and autophagic capacity in *KRAS*-mutant PDAC; a counter-prediction	Reduced	LAMP1 colocalisation; cycloheximide chase	Hypothesis-generating
Recycling (normally minimal)	Rab11a; suppressed by receptor ubiquitination	Increased if ubiquitination falls, phenocopying PAR2Δ14K/R	Increased	Rab11 colocalisation; surface re-appearance after agonist washout	Directly demonstrated in ubiquitination-deficient mutants.
Replenishment from the Golgi pool	Palmitoylation at cysteine 361; Rab11a-dependent delivery	Accelerated by anabolic and secretory flux	Increased, through faster resensitisation	Brefeldin A; 2-bromopalmitate; recovery of trypsin-evoked calcium transients	Supported by indirect evidence.

**Table 3:** Comparative biology of PAR-2 signalling in colorectal cancer and pancreatic ductal adenocarcinoma.

Feature	Colorectal Cancer (CRC)	Pancreatic Ductal Adenocarcinoma (PDAC)
Dominant disease context	Epithelial barrier dysfunction; microbiome–epithelium interface; chronic inflammation; adenoma–carcinoma sequence; immune editing.	Desmoplasia; coagulation activation; protease-rich pancreas; perineural invasion; immune exclusion; cachexia.
Key oncogenic background	*APC*/Wnt loss, *KRAS*/*BRAF* mutation, MSI-high vs. *CIN*, *frequent TP53 mutation in advanced disease.*	Near-universal mutant *KRAS*, frequent TP53, SMAD4 and CDKN2A loss; very high stromal content.
Principal PAR-2-activating proteases	Microbial proteases (e.g., *Bacteroides fragilis* fragipain), trypsin-like enzymes, mast-cell tryptase, neutrophil proteases, coagulation FXa/FVIIa.	Pancreatic trypsin-like enzymes, tissue factor–FVIIa/FXa, neutrophil elastase, mast-cell tryptase, CAF-derived proteases.
Dominant PAR-2 outputs	NF-κB-driven inflammation, Wnt/β-catenin amplification, ERK/MAPK survival signalling, Bcl-xL stabilisation, EMT and barrier remodelling.	TGF-β/ALK5-dependent motility, ATP-dependent migration, Rho-GTPase remodelling, ERK/AKT amplification, fibroinflammatory CAF skewing.
Highest-yield translational opportunity	EGFR-therapy sensitisation; statin and nutraceutical repurposing; biomarker-guided immunotherapy combinations.	Anticoagulant repurposing (FXa inhibitors); stromal modulation; combined antitumour and anti-pain strategies; chemo-sensitisation.
Principal cautions	Tumour-intrinsic PAR-2 may sustain CD8^+^ antitumour immunity; universal blockade may be deleterious in immune-hot tumours.	Stromal depletion can accelerate tumour progression; selective rather than blanket PAR-2 modulation is required.

**Table 4:** Drug-repurposing and direct-targeting candidates for the protease–PAR-2 axis in colorectal and pancreatic cancer.

Agent/Class	Mechanism vs. PAR-2 Axis	Pathway Affected	Evidence in CRC	Evidence in PDAC	Clinical Feasibility/Caveats
I-287 and analogues (PAR-2 negative allosteric modulators)	Selective inhibition of Gαq and Gα12/13 PAR-2 signalling without disrupting β-arrestin recruitment.	Calcium, RhoA, ERK/NF-κB.	Proof-of-concept anti-inflammatory effects in vitro.	Mechanistic rationale strong; direct CAF/PDAC data limited.	Pre-clinical; biased pharmacology preserves cytoprotective signalling.
AZ3451 (orthosteric crystallographic ligand)	Allosteric blockade demonstrated by structural studies.	Gαq, Gα12/13 outputs.	In vitro efficacy in inflammatory and cancer models.	Useful as chemical tool; in vivo data limited.	Tool compound; not yet a clinical candidate.
Atorvastatin/rosuvastatin	HMG-CoA reductase inhibition; selective downregulation of PAR-2 expression and PAR-2-driven calcium and TNF-α responses.	PAR-2/ERK; Rho-GTPase prenylation; NF-κB.	Selective PAR-2 attenuation in HT-29 and Caco-2 cells; population-level chemopreventive signal.	Mechanistic plausibility; direct PAR-2 data in PDAC pending.	Clinically deployable; risk-benefit favourable; lipophilic statins likely most active.
Apixaban/rivaroxaban/edoxaban (direct FXa inhibitors)	Suppress upstream coagulation-protease activation of PAR-2.	FXa–PAR-2 → ERK, NF-κB, IL-8/VEGF.	Edoxaban suppresses CRC colon26 tumour growth in BALB/c mice [25]; FXa inhibition blocks PAR-2-driven CRC growth in vitro and in vivo.	Strong mechanistic rationale via TF–FXa–PAR-2; clinical anticoagulation already standard in PDAC.	Bleeding risk; biomarker-stratified trials warranted.
Curcumin	Direct binding to PAR-2 (computational docking); suppression of PAR-2/ERK/TNF-α and re-activation of apoptosis.	PAR-2/ERK/NF-κB; calcium; Bcl-2 family.	Inhibits PAR-2-induced ERK phosphorylation, calcium mobilisation and anti-apoptotic signalling in CRC cells.	Plausible mechanism; clinical data heterogeneous.	Bioavailability remains the principal limitation; nano-formulations under study.
Oleocanthal	Selective modulation of PAR-2-driven inflammatory output.	PAR-2/NF-κB; Wnt/β-catenin; calcium; TNF-α.	Attenuates TNF-α and calcium dysregulation in CRC cell models.	Mechanistic extension plausible; direct PDAC data limited.	Dietary-bioactive; suitable for chemoprevention strategies.
EGCG and other polyphenols	Multi-pathway modulation including PAR-2-coupled inflammatory and TGF-β signalling.	PAR-2; NF-κB; STAT3; TGF-β/ALK5.	Anti-inflammatory and anti-proliferative effects in CRC cell systems.	Less direct PDAC evidence specific to PAR-2.	Bioavailability and dose–response require optimisation.
PAR-2 + EGFR-targeted therapy combination	Restoration of RNF152-mediated Bcl-xL ubiquitination via PAR-2 inhibition; sensitisation to cetuximab/panitumumab.	PAR-2/Bcl-xL/RNF152 axis; EGFR–RAS–RAF.	PAR-2 inhibition resensitises CRC PDX models to multiple EGFR-targeted therapies.	Hypothesis-generating extension to *KRAS*-WT PDAC.	Most compelling near-term combination strategy in RAS/RAF-WT CRC.

**Table 5:** Determinants of the serum PRO-PAR2 signal, their predicted behaviour across tumour stages and in benign inflammatory disease, and design strategies to improve discrimination. The downward arrow (↓) identifies the proteolytic cleavage site in each source description.

Proteolytic Source	Predicted Behaviour with CRC or PDAC Stage	Behaviour in Benign Inflammatory Disease	Consequence for Discrimination	Design Response
Mast-cell tryptase (canonical R36↓S37)	Rises modestly from adenoma to carcinoma; highest at the invasive front	Markedly raised in inflammatory bowel disease, rheumatoid arthritis, asthma and allergic disease	Poor discrimination at early stage; overlapping distributions	Include an active inflammatory bowel disease comparator arm; pair with tissue tryptase-positive mast-cell density
Matriptase, following loss of the HAI-1/*SPINT1* brake	Increases early, at the adenoma to carcinoma transition	Raised in chronic mucosal injury and psoriasis	Early-stage signal overlaps benign mucosal disease	Report a matriptase to HAI-1 ratio; anchor to tissue immunohistochemistry
TF–FVIIa–FXa coagulation axis	Rises steeply in advanced and metastatic disease, especially tissue-factor-high PDAC	Raised in sepsis, venous thromboembolism, and postoperative and acute inflammatory states	Drives the advanced-stage signal but overlaps thrombotic and septic states	Record and exclude intercurrent thromboembolism, sepsis and recent surgery; co-measure D-dimer and prothrombin fragment 1 + 2
Neutrophil elastase, cathepsin G, proteinase-3 (non-canonical, S67↓V68)	Rises in neutrophil-rich tumours	Markedly raised in acute infection, acute respiratory distress and active colitis	Large non-specific component; fragment kinetics also differ because the elastase-cleaved receptor is not ubiquitinated	Deploy cleavage-site-specific neoepitope assays to resolve protease provenance
Microbial proteases at a disrupted barrier	Contributes chiefly in colitis-associated CRC	High in dysbiosis, active colitis and infective enteritis	Confounds CRC specifically	Pair serum measurement with faecal protease activity
Systemic inflammatory tone (interleukin-6 driven)	Rises with tumour burden and cachexia	Definitionally raised	Dominant confounder across all stages	Index to C-reactive protein or interleukin-6; prefer longitudinal intra-patient change
Fragment clearance (renal and hepatic)	Varies with performance status and organ dysfunction	Variable	Alters fragment half-life independently of production	Record estimated glomerular filtration rate and liver function; consider clearance-adjusted normalisation

**Table 6:** Biomarker pipeline for the protease–PAR-2 axis in gastrointestinal cancer.

Biomarker	Sample Type	Assay	What It Measures	Strengths and Limitations
*F2RL1* mRNA	Tumour tissue/RNA-seq	qPCR; bulk and single-cell transcriptomics; spatial transcriptomics.	PAR-2 transcriptional output; cellular distribution.	Widely available; does not capture cleavage or activation state.
Total PAR-2 protein	Tumour tissue; cell lysates	Western blot; immunohistochemistry.	Receptor abundance.	Antibody specificity remains a critical caveat; activation status not captured.
Cell-surface PAR-2	Disaggregated tumour; cell lines; PBMCs	Flow cytometry with N-terminal-recognising antibodies.	Surface receptor available for cleavage.	Sensitive to internalisation kinetics; sample-handling-dependent.
Cleaved/activated PAR-2 (PRO-PAR2)	Serum/plasma	Neoepitope ELISA targeting the activation fragment.	Systemic PAR-2 activation by serine proteases.	Validated in inflammatory disease; cancer-specific clinical thresholds remain to be established; not tumour-specific.
Phospho-ERK/phospho-NF-κB/phospho-AKT	Tumour tissue; circulating tumour cells	IHC; phosphoproteomic mass spectrometry.	Downstream pathway activation.	Multifactorial inputs; PAR-2-attribution requires perturbation studies.
Tissue factor/FVIIa/FXa activity	Tumour tissue; plasma	IHC for TF; chromogenic FXa assays; circulating microparticle TF activity.	Coagulation arm of the PAR-2 axis.	Strong rationale in PDAC; available in clinical chemistry laboratories.
Faecal protease activity	Stool	Trypsin-like activity, gelatin/azocasein zymography, microbial protease metagenomics.	Microbiome-derived proteolytic burden.	Pre-analytical variability; promising stratifier in CRC and IBD-CRC.
Bcl-xL/phospho-Bcl-xL (Ser-145)	Tumour tissue	IHC; phosphoproteomics.	PAR-2-dependent anti-apoptotic node in CRC.	Companion biomarker for PAR-2 + EGFR-therapy combinations.
Mast-cell tryptase density	Tumour tissue	Tryptase IHC.	Local proteolytic input to PAR-2.	Prognostic in CRC; complementary to *F2RL1* signature.

**Table 7:** Existing survival evidence for individual components of the proposed protease–PAR-2 signature, and the untested status of the composite signature.

Component and Analyte	Setting and Cohort	Endpoint and Method	Direction of Association	Independent in Multivariable Analysis
PAR-2 immunohistochemistry with tryptase-positive mast-cell density	Colorectal cancer; 115 patients	Overall survival; Kaplan–Meier	High density is adverse	Reported as a poor prognostic factor
PAR-2 protein (tissue microarray immunohistochemistry)	Hepatitis-B-related hepatocellular carcinoma; 202 resections	Overall and disease-free survival; Kaplan–Meier with Cox regression	High expression is adverse	Yes; independent for both endpoints
*F2RL1*/PAR-2 expression, tumour-cell restricted	Colorectal cancer; human cohort with public dataset support	Prognosis and CD8+ infiltration	High expression is favourable; opposite in direction to the above	Not reported as an adjusted composite
Plasma PRO-PAR2 activation fragment	Non-small-cell lung cancer; pembrolizumab with platinum	Overall survival; Kaplan–Meier by median split	Above median is adverse	Not reported
*F2RL1* expression as a predictive marker	Solid tumours treated with immune checkpoint inhibitors	Response to checkpoint blockade	High expression predicts non-response	Not reported
Tissue factor (immunohistochemistry)	Pancreatic ductal adenocarcinoma	Grade, invasion, thromboembolism; survival in selected series	High expression is adverse	Variable across series
Composite protease–PAR-2 signature as proposed in this review	Not yet assembled in any cohort	Not tested	Not tested	Not tested

**Table 11:** Pro-tumour versus potentially protective and context-editing effects of PAR-2 across biological compartments and disease stages in gastrointestinal cancer. Evidence direction is colour-coded: green = potentially protective; amber = ambiguous, paradoxical or stage-dependent; black = pro-tumour.

Biological Context	Dominant PAR-2 Compartment	Evidence Direction	Stage/ Setting	Mechanistic Interpretation	Net Biological Consequence	Therapeutic Implication
Inflammation-driven CRC: HAI-1/*SPINT1* loss, *Apc*^Min/+^ background	Tumour epithelium and inflamed mucosa	Pro-tumour	Adenoma initiation and early tumour expansion	Loss of HAI-1 permits deregulated trypsin-like protease activity, leading to PAR-2 activation, NF-κB signalling, cytokine induction, VEGF expression and angiogenesis. *F2rl1* co-deletion rescues the tumour-promoting phenotype [49,50].	Accelerated intestinal tumorigenesis, angiogenesis and inflammatory progression	Strong rationale for PAR-2/protease-axis inhibition in inflammation-dominant CRC
Advanced CRC with high epithelial PAR-2 expression	Tumour epithelial cells	Pro-tumour	Adenoma–carcinoma progression, invasive front	PAR-2 activates ERK/MAPK, AKT, NF-κB, Wnt/β-catenin and Rho-GTPase outputs, supporting survival, EMT, migration and invasion [193].	Tumour growth, invasion, metastatic competence	Direct PAR-2 antagonism or downstream ERK/AKT/NF-κB targeting may be useful in PAR-2-high tumours
CRC resistance to EGFR-targeted therapy	Tumour epithelial cells	Pro-tumour	Treatment-resistant metastatic CRC	PAR-2 signalling stabilises Bcl-xL and interferes with ubiquitin-mediated degradation pathways, thereby reducing apoptotic response to EGFR inhibition [22].	Cetuximab/panitumumab resistance	PAR-2 inhibition may resensitise RAS/RAF-wild-type CRC to EGFR antibodies
Mast-cell-rich CRC microenvironment	Mast cells → tumour epithelium; stromal interface	Pro-tumour	Inflamed mucosa, invasive tumour edge	Mast-cell tryptase activates PAR-2, promoting calcium signalling, ERK/MAPK, NF-κB activation, MYO10-dependent motility, angiogenesis and stromal cross-talk [5].	Invasion, angiogenesis, barrier disruption and inflammatory amplification	Tryptase blockade or mast-cell modulation may complement PAR-2 inhibition
Neutrophil-rich CRC or PDAC	Neutrophils, tumour cells, stromal cells	Ambiguous/context-editing	Chronic inflammation, aggressive neutrophil-enriched tumours	Neutrophil elastase, cathepsin G and proteinase 3 may cleave PAR-2 non-canonically, either disarming canonical signalling or redirecting β-arrestin/MAPK-biased outputs [34].	PAR-2 signal editing rather than simple activation; may promote inflammation, desensitisation or altered immune signalling depending on protease dominance	Avoid interpreting SLIGKV/trypsin peptide data as fully representative of neutrophil-rich tumours
MSI-high/immune-hot CRC	Tumour epithelial cells and immune interface	Potentially protective	Immunocompetent CRC; checkpoint-responsive setting	Tumour-intrinsic PAR-2 may sustain chemokine output, CD8^+^ T-cell infiltration and effector function. PAR-2 loss may reduce immune visibility and impair anti-PD-1 efficacy [8,26].	Maintains antitumour immune surveillance in selected contexts	Universal PAR-2 blockade may be harmful; immune contexture must guide intervention
Inflammation-dominant, immune-suppressed CRC	Tumour epithelium, myeloid cells, stromal cells	Pro-tumour	IBD-associated or cytokine-rich CRC	FXa-, tryptase-, microbial- or epithelial-protease-driven PAR-2 activation amplifies NF-κB, IL-6, IL-8, TNF-α, VEGF and myeloid recruitment [49].	Pathological inflammation, angiogenesis and immunosuppressive remodelling	PAR-2-axis suppression is most rational where inflammation is tumour-promoting rather than immune-protective
PDAC with tissue-factor/coagulation activation	Tumour epithelial cells, endothelial cells, CAFs	Pro-tumour	Established and advanced PDAC	Tumour-cell tissue factor drives TF–FVIIa–FXa signalling, activating PAR-2 and engaging ERK, AKT, NF-κB, IL-8, VEGF and survival pathways [27,29,163].	Angiogenesis, invasion, stromal activation, chemoresistance	FXa inhibitors or TF–FVIIa–PAR-2 modulation may be rational in TF-high PDAC
Desmoplastic PDAC stroma	CAFs, tumour cells, endothelial cells	Pro-tumour but stromally complex	Established PDAC; immune-excluded tumours	PAR-2 intersects with TGF-β/ALK5/SMAD, RhoA/ROCK, ECM remodelling and CAF activation [27,29,163].	Matrix stiffness, immune exclusion, invasion and drug resistance	Selective modulation is preferable to blanket stromal depletion
PDAC endothelial compartment	Endothelial cells	Pro-tumour	Angiogenic and metastatic niche	TF–FVIIa/PAR-2 signalling induces IL-8, CXCL1 and VEGF, increasing vascular permeability, leukocyte adhesion and angiogenic remodelling [27,29,163].	Neoangiogenesis and metastatic niche formation	Endothelial PAR-2 activity may serve as a vascular biomarker of coagulation-linked tumour activation
Barrier-disrupted, microbiome-rich CRC	Intestinal epithelium, microbial protease interface	Mostly pro-tumour, but disease-stage dependent	Chronic colitis, dysbiosis, early carcinogenesis	Microbial proteases may activate epithelial PAR-2, disrupting barrier integrity and amplifying mucosal inflammation [146].	Barrier dysfunction, inflammation-driven carcinogenesis	Microbial protease targeting may be useful in colitis-associated CRC prevention
Immunotherapy-treated CRC	Tumour epithelium and immune cells	Paradoxical/biomarker-dependent	MSI-high, CD8-rich or checkpoint-treated disease	PAR-2 may either promote inflammatory tumour growth or maintain chemokine-mediated immune visibility [8,26].	Either immune exclusion or enhanced immune surveillance depending on context	PAR-2 targeting should be combined with MSI status, CD8 density, cytokine profile and activated-PAR-2 biomarkers

**Table 12:** Molecular determinants of the direction of PAR-2 output in tumour immunity, with the configurations favouring pro-tumour and immune-supportive phenotypes. The downward arrow (↓) indicates the proteolytic cleavage site.

Determinant	Configuration Favouring Pro-Tumour Output	Configuration Favouring Immune-Supportive Output	Mechanistic Basis	Evidence Strength
Cellular compartment	Myeloid cells: macrophages and myeloid-derived suppressor cells	Tumour epithelium and conventional type-1 dendritic cells	Myeloid PAR-2 impairs antigen-presentation programmes and sustains immunosuppressive populations; epithelial PAR-2 drives CXCL9/CXCL10; dendritic-cell PAR-2 is required for cDC1 differentiation and CD8+ priming	Directly demonstrated
Activating protease and cleavage site	Neutrophil elastase (S67↓V68), cathepsin G, proteinase-3	Trypsin and mast-cell tryptase (canonical R36↓S37)	Non-canonical cleavage disables Gαq-coupled calcium signalling and biases output to β-arrestin/MAPK; canonical cleavage engages the full transducer repertoire	Directly demonstrated
Post-endocytic fate of the cleaved receptor	Elastase-cleaved receptor escapes ubiquitination, giving persistent biased signalling	Trypsin-cleaved receptor is ubiquitinated by c-Cbl and degraded, giving self-limiting signalling	c-Cbl-dependent, ESCRT-mediated lysosomal sorting terminates signalling; the elastase-cleaved receptor is not ubiquitinated	Directly demonstrated
Transducer branch dominance	Gαq–phospholipase C–calcium–NF-κB, generating interleukin-6, interleukin-8, CXCL1 and VEGF	PI3K–AKT–mTOR, generating CXCL9 and CXCL10	An inflammatory secretome recruits and polarises myeloid cells; a CXCR3-ligand secretome recruits effector CD8+ T cells	Directly demonstrated for the chemokine arm; branch competition is hypothesis-generating
Baseline immune contexture	Immune-desert, myeloid-rich, microsatellite-stable tumours	T-cell-inflamed, microsatellite-instability-high, CD8-rich tumours	Availability of an effector pool determines whether CXCL9/CXCL10 output is productive	Supported by indirect evidence
Microenvironmental pH	Acidic microenvironment alters agonist potency and protease activity	Physiological pH preserves canonical activation efficiency	PAR-2 activation is pH-sensitive, with agonist potency differing several-fold between pH 6.5 and 7.4	Supported by indirect evidence
Disease stage	Advanced, coagulation-activated disease	Early, immune-surveilled disease	The dominant proteolytic input shifts from mucosal and mast-cell derived to coagulation dominated as disease advances	Hypothesis-generating

**Table 13:** Audit of PAR-2 activation strategies in the primary studies cited in this review, and the consequences of each for the interpretation of signalling bias.

Activator Class	Approximate Share of the Audited Corpus	What It Models Faithfully	What It Cannot Model	Consequence for Inference
Synthetic activating peptide alone (SLIGRL-NH2, SLIGKV-NH2, 2-furoyl-LIGRLO-NH2)	Approximately 30%	Canonical orthosteric engagement; Gαq-coupled calcium signalling; receptor-proximal pharmacology; rank-order potency	Proteolysis; cleavage-site selection; protease identity; inhibitor tone; spatial confinement; non-canonical bias; receptor disarming; ubiquitination-dependent trafficking	Biases the literature systematically towards canonical Gαq output and is categorically blind to elastase- and cathepsin-G-driven biology
Native or recombinant protease alone (trypsin, tryptase, matriptase, FVIIa, FXa, elastase)	Approximately 40%	Physiological activation including cleavage-site selection, biased output and post-endocytic fate	Preparations vary in purity and specific activity; bovine trypsin is a supraphysiological surrogate for human tryptases	The most informative class, but activity standardisation across studies is inconsistent and rarely reported
Both peptide and protease	Approximately 20%	Direct attribution of an output to proteolysis as distinct from orthosteric engagement	No categorical limitation; limited by the quality of the protease preparation	The methodological reference standard; should become the norm for mechanistic work
Genetic or pharmacological manipulation without exogenous activation (*F2rl1* deletion, silencing, antagonist or negative allosteric modulator)	Approximately 10%	Necessity of the receptor within an intact system, including immune and stromal context	Sufficiency; which protease is responsible; which cleavage site is used; which transducer is engaged	Complementary and indispensable in vivo, but cannot resolve signalling bias

## Data Availability

No new data were created or analyzed in this study. Data sharing is not applicable to this article.

## Abbreviations

ALK5: type-I TGF-β receptor (activin receptor-like kinase 5); AMSH: associated molecule with the SH3 domain of STAM; apCAF: antigen-presenting cancer-associated fibroblast; Bcl-xL: B-cell lymphoma-extra large; Bfp1: *Bacteroides fragilis* serine protease 1; BRET: bioluminescence resonance energy transfer; CaMKII: calcium/calmodulin-dependent protein kinase II; CAF: cancer-associated fibroblast; CRC: colorectal cancer; CSC: cancer stem cell; CXCL1: C-X-C motif chemokine ligand 1; DUSP6: dual specificity phosphatase 6; ECL2: extracellular loop 2; ECM: extracellular matrix; EGCG: epigallocatechin gallate; EMT: epithelial–mesenchymal transition; ESCRT: endosomal sorting complex required for transport; EZH2: enhancer of zeste homologue 2; FAK: focal adhesion kinase; *F2RL1*: coagulation factor II receptor-like 1; FXa: factor Xa; GPCR: G-protein-coupled receptor; GRK: G-protein-coupled receptor kinase; GSK-3β: glycogen synthase kinase 3 beta; HAI-1: hepatocyte growth factor activator inhibitor type 1; HMG-CoA: 3-hydroxy-3-methylglutaryl-coenzyme A; HRS: hepatocyte growth factor-regulated tyrosine kinase substrate; IBD: inflammatory bowel disease; iCAF: inflammatory cancer-associated fibroblast; IL: interleukin; IP3: inositol trisphosphate; *KRAS*: Kirsten rat sarcoma viral oncogene homologue; LAMP1: lysosomal-associated membrane protein 1; LATS1/2: large tumour suppressor kinase 1/2; LPS: lipopolysaccharide; MAPK: mitogen-activated protein kinase; MCP-1: monocyte chemoattractant protein-1; MCL-1: induced myeloid cell differentiation protein MCL-1; MEK: mitogen-activated protein kinase kinase; MMP: matrix metalloproteinase; MSI: microsatellite instability; myCAF: myofibroblastic cancer-associated fibroblast; MYO10: myosin X; NHERF1: ${\mathrm{Na}}^{+}/H^{+}$ exchange regulatory cofactor 1; NF-κB: nuclear factor kappa-light-chain-enhancer of activated B cells; NFAT: nuclear factor of activated T cells; OATP: organic anion-transporting polypeptide; PAR: protease-activated receptor; PANC-1: pancreatic cancer cell line 1; PDAC: pancreatic ductal adenocarcinoma; PDGF: platelet-derived growth factor; PI3K: phosphoinositide 3-kinase; PIP2: phosphatidylinositol 4,5-bisphosphate; PLC: phospholipase C; Rab: Ras-related in brain protein; RAF: rapidly accelerated fibrosarcoma; RNF: ring finger protein; ROCK: Rho-associated protein kinase; SMAD: small mothers against decapentaplegic; *Spint1*: serine peptidase inhibitor, Kunitz type 1; Src: sarcoma proto-oncogene tyrosine-protein kinase; STAM: signal transducing adaptor molecule; TAZ: transcriptional coactivator with PDZ-binding motif; TCF/LEF: T-cell factor/lymphoid enhancer-binding factor; TF: tissue factor; TGF-$\beta$: transforming growth factor beta; TNM: tumour, node, metastasis staging system; TRP: transient receptor potential channel; UBPY: ubiquitin-specific-processing protease Y; uPA: urokinase plasminogen activator; USP8: ubiquitin specific peptidase 8; VEGF: vascular endothelial growth factor; YAP: yes-associated protein.
